# In a randomized trial, the live attenuated tetravalent dengue vaccine TV003 is well-tolerated and highly immunogenic in subjects with flavivirus exposure prior to vaccination

**DOI:** 10.1371/journal.pntd.0005584

**Published:** 2017-05-08

**Authors:** Stephen S. Whitehead, Anna P. Durbin, Kristen K. Pierce, Dan Elwood, Benjamin D. McElvany, Ellen A. Fraser, Marya P. Carmolli, Cecilia M. Tibery, Noreen A. Hynes, Matthew Jo, Janece M. Lovchik, Catherine J. Larsson, Elena A. Doty, Dorothy M. Dickson, Catherine J. Luke, Kanta Subbarao, Sean A. Diehl, Beth D. Kirkpatrick

**Affiliations:** 1 Laboratory of Infectious Diseases, National Institutes of Allergy and Infectious Diseases, National Institutes of Health, Bethesda, Maryland, United States of America; 2 Center for Immunization Research, Johns Hopkins School of Public Health, Baltimore, Maryland; United States of America; 3 Vaccine Testing Center, Department of Medicine, University of Vermont Larner College of Medicine, Burlington, Vermont, United States of America; Oregon Health and Science University, UNITED STATES

## Abstract

Infection caused by the four serotypes of dengue virus (DENV-1-4) is a leading cause of mosquito-borne disease. Clinically-severe dengue disease is more common when secondary dengue infection occurs following prior infection with a heterologous dengue serotype. Other flaviviruses such as yellow fever virus, Japanese encephalitis virus, and Zika virus, can also elicit antibodies which are cross-reactive to DENV. As candidate dengue vaccines become available in endemic settings and for individuals who have received other flavivirus vaccines, it is important to examine vaccine safety and immunogenicity in these flavivirus-experienced populations. We performed a randomized, controlled trial of the National Institutes of Health live attenuated tetravalent dengue vaccine candidate (TV003) in fifty-eight individuals with prior exposure to flavivirus infection or vaccine. As in prior studies of this vaccine in flavivirus-naive volunteers, flavivirus-experienced subjects received two doses of vaccine six months apart and were followed closely for clinical events, laboratory changes, viremia, and neutralizing antibody titers. TV003 was well tolerated with few adverse events other than rash, which was predominately mild. Following one dose, 87% of vaccinees had an antibody response to all four serotypes (tetravalent response), suggesting a robust immune response. In addition, 76% of vaccinees were viremic; mean peak titers ranged from 0.68–1.1 log_10_ PFU/mL and did not differ by serotype. The second dose of TV003 was not associated with viremia, rash, or a sustained boost in antibody titers indicating that a single dose of the vaccine is likely sufficient to prevent viral replication and thus protect against disease. In comparison to the viremia and neutralizing antibody response elicited by TV003 in flavivirus-naïve subjects from prior studies, we found that subjects who were flavivirus-exposed prior to vaccination exhibited slightly higher DENV-3 viremia, higher neutralizing antibody titers to DENV-2, -3, and -4, and a higher tetravalent response frequency after TV003 administration. In summary, we demonstrate that the NIH tetravalent dengue vaccine TV003 is well-tolerated in flavivirus-experienced individuals and elicits robust post-vaccination neutralizing antibody titers.

**Trial registration:**

ClinicalTrials.gov NCT01506570

## Author summary

As live-attenuated dengue vaccine candidates are developed, it is important to ascertain their safety in all populations, regardless of past exposure to dengue, closely related flaviviruses, or similar vaccines. Each of the four dengue virus (DENV) serotypes can cause clinical disease. Severe dengue disease may be life-threatening and is epidemiologically linked to secondary infection with a serotype distinct from the first infection. Candidate tetravalent dengue vaccines are designed to induce neutralizing antibody responses to all serotypes, but confirmation is needed that vaccination itself, as a secondary exposure, is not associated with the development of enhanced reactogenicity. The National Institutes of Health live attenuated tetravalent dengue vaccine candidate, TV003, has previously been shown to be safe and immunogenic in flavivirus-naïve populations. We performed a randomized, placebo-controlled clinical trial of TV003 in individuals previously exposed to flaviviruses and demonstrated tolerability and strong, broad immunogenicity across serotypes. No subjects experienced any dengue-like illness. Vaccine viremia was self-limited and occurred at acceptably low levels compared to those associated with severe dengue from natural infection. TV003 is well-tolerated in healthy adults, regardless of flavivirus exposure, and will be evaluated next in DENV-endemic settings.

## Introduction

The four serotypes of dengue virus (DENV-1 to 4) are the major cause of mosquito-borne viral disease globally. Approximately 40% of the world’s population is at risk of dengue infection and all serotypes cause clinical disease [1, 2]. The incidence of dengue is increasing dramatically throughout the world, both in regions with known disease and in new areas where the mosquito vectors *Aedes aegypti* and *Aedes albopictus* have expanded [3].

Dengue infection causes a spectrum of clinical disease from subclinical infection (most common) to a life-threatening vascular leak vascular leak syndrome [4]. Classic dengue fever, which consists of high fever, myalgia, and rash, as well as neutropenia and thrombocytopenia, is commonly seen in primary infection and is self-limiting. Primary infection leads to life-long protection from symptomatic homotypic infection, but only short-lived cross-protection from infection with the other serotypes [5]. Severe disease (dengue shock syndrome and/or hemorrhagic fever) may be associated with organ impairment, plasma leakage, and the need for fluid management, and may be fatal in the absence of appropriate medical care. Severe disease occurs most frequently in infants and young children, although all ages are affected [6].

Although the full spectrum of illness can be seen with primary dengue, most cases of severe disease are observed with secondary, heterotypic DENV infection. Antibody-dependent enhancement of infection (ADE) is thought to be a significant contributor to pathophysiology. In the ADE model, antibodies elicited to the serotype seen in the primary infection are still binding, yet do not effectively neutralize the new serotype and may “enhance” entry of the new DENV serotype into susceptible cells (e.g. monocytes) through Fcγ receptors, leading to increased viral replication and viremia [7–9]. The level of DENV viremia has been positively associated with dengue disease severity [10, 11]. Following recovery from secondary dengue infection or disease, the risk of severe disease is very low upon subsequent infection with either of the two remaining serotypes (i.e. postsecondary exposures), suggesting that protective immunity does progressively develop [12, 13].

Over the past decade there has been progress in the development of candidate dengue vaccines, several of which are live-attenuated vaccines containing antigens from all four DENV serotypes (tetravalent vaccines) [14–21]. One of these (Sanofi-Pasteur’s Dengvaxia, a chimeric yellow fever dengue–tetravalent dengue vaccine, CYD-TDV) has been recently approved for limited use in several countries. In CYD-TDV, the DENV structural proteins from each serotype have been inserted into the yellow fever virus (YFV) non-structural genetic backbone. CYD-TDV efficacy was significantly lower in those persons who were dengue-naïve at the time of vaccination and in younger, predominantly flavivirus-naïve, age groups [16, 17]. During the first year of the long-term safety follow-up period, a higher rate of hospitalization due to dengue was observed in CYD-TDV recipients who were under 9 years of age at the time of vaccination compared to placebo recipients who were under 9 years of age at the time of dosing [16, 17]. For these reasons, the WHO Strategic Advisory Group of Experts on Immunization has recommended limited use of the vaccine to individuals 9–45 years of age in highly dengue-endemic areas (dengue seroprevalence ≥ 70%) [22].

The goal of DENV vaccination is to achieve protection from disease caused by each of the four serotypes. There is concern that if the immunologic responses following vaccination are imbalanced across serotypes, subsequent infection by serotype(s) for which the vaccine did not induce a protective immune response may result in more severe illness due to ADE. It has been postulated [23] that ADE may explain recent findings with CYD-TDV, in which vaccinated children 2–5 years of age exhibited a 7.45-fold higher rate of dengue hospitalizations compared to unvaccinated children in the same age group [15, 17]. Theoretically, replication of a live DENV vaccine virus could also be increased by cross-reactive antibodies in persons previously exposed to DENV or another flavivirus, although it is unlikely that the level of dengue vaccine viremia would achieve a level associated with clinical disease.

The NIH live-attenuated tetravalent dengue vaccine TV003 is attenuated by one or more 30-nucleotide deletions in the 3’ untranslated region of the viral genome, which limits replication *in vivo* [24]. As shown in a series of previous Phase I clinical trials, this single-dose vaccine is safe and immunogenic against all four dengue serotypes, inducing a tetravalent antibody response in over two-thirds of subjects after a single dose [19, 25–27]. TV003 provided complete protection against experimental infection with a DENV-2 challenge strain [26]. To further document the safety of the TV003 tetravalent dengue vaccine in subjects who may have had prior flavivirus exposure, we performed a randomized-controlled clinical trial of TV003 in healthy, flavivirus-experienced adults. Prior to this study, evaluation of the TV003 vaccine had been performed exclusively in flavivirus-naïve adult cohorts [19, 25, 28].

## Materials and methods

### Ethics statement

This study was performed under an FDA-reviewed investigational new drug application and was approved by the Institutional Review Boards of Johns Hopkins University and the University of Vermont. Written informed consent was obtained in accordance with federal and international regulations (21CFR50, ICHE6) and Good Clinical Practices (GCP) were followed throughout. The NIAID Data Safety Monitoring Board reviewed all safety data every 6 months and external independent monitoring was performed.

### Trial design and study setting

**Fig 1:**
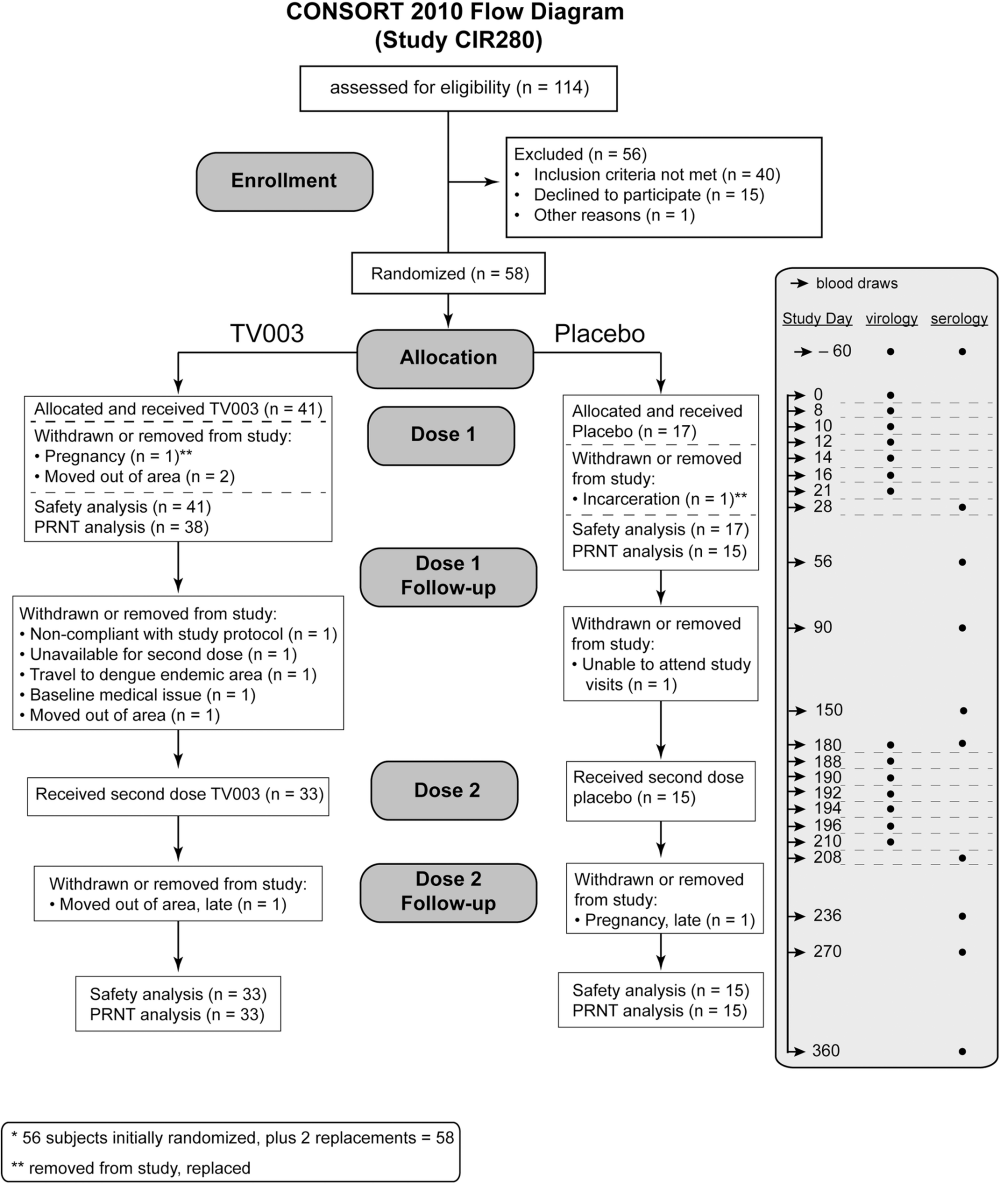
CONSORT diagram of enrollment and follow up of subjects for first and second dosings of TV003 in subjects who were flavivirus-exposed prior to vaccination with TV003 (Study CIR280). The schedule of blood draws for serological and virological testing is indicated in the shaded box at right.

This Phase 1 randomized, double-blind, placebo-controlled trial was conducted in Baltimore, MD and Burlington, VT. Study subjects were enrolled under study protocol CIR 280 (ClinicalTrials.gov NCT01506570). The study evaluated the safety and immunogenicity of the NIH live attenuated tetravalent vaccine (TV003) in 58 flavivirus-exposed adult subjects. To determine the effect of the second vaccination on frequency of seroconversion, tetravalent response, and mean neutralizing antibody titer, a second dose of the same vaccine was administered six months following the first dose. Subjects received the same assignment (vaccine or placebo) for both doses (**Fig 1)**. Subjects were randomized using a random-number generator into blocks of seven, with each block containing five vaccine and two placebo recipients. Study teams from the clinical sites were blinded to treatment assignment. Data was unblinded 270 days after all subjects within a block received the first treatment dose. The clinical trial protocol is included in **Supporting Information**.

### Study population

Healthy adults, aged 18–50, from the study sites were recruited and enrolled. Eligibility criteria were as previously described [19], with the addition of an inclusion criterion requiring evidence of exposure to any of the following flaviviruses: DENV1-4, YFV, Japanese encephalitis virus (JEV), West Nile virus (WNV), St. Louis Encephalitis virus (SLEV), or tick-borne encephalitis virus (TBEV). Serological evidence consisted of neutralizing antibodies to DENV-1-4, YFV, WNV, JEV, SLEV, or TBEV. Documented history of vaccination with YFV, JEV, or an experimental DENV vaccine was also accepted as demonstration of past exposure to flaviviruses. Eligible subjects were required to be seronegative for hepatitis B, hepatitis C, and human immunodeficiency viruses and were required to have normal blood hematology, serum chemistry, and physical examination findings.

### Investigational product/ vaccine

The live attenuated dengue vaccine (TV003) contains all four dengue serotypes (DENV1-4) in a tetravalent admixture. The vaccine constructs, which are based on limiting viral replication by deleting a 30-nucleotide sequence in the 3’ untranslated region of the viral genome(s), are described elsewhere [24]. The development of the tetravalent formulations, the safety and immunogenicity of the vaccine components, and selection of TV003 as a lead candidate is also described elsewhere [28]. The DENV-2 component, rDEN2/4Δ30 was further attenuated by chimerization in which the prM and E proteins of DENV-2 replace those of DENV-4 in the rDEN4Δ30 construct. TV003 contains rDENV1Δ30 [29], rDEN2/4Δ30 [30], rDENV3Δ30,31 [28], or rDENV4Δ30 [31]. Vaccine components were produced as seed viruses in Vero cells in the Laboratory of Infectious Diseases (LID), NIAID, and the vaccine was manufactured in Vero cells at Charles River Laboratories (CRL) Biopharmaceutical Services facility in Malvern, PA (rDENV1Δ30, DENV2/4D30, and rDENV4Δ30 components) or Meridian Life Science, Memphis TN (DEN3Δ30/31 component) under GMP conditions. Viral stocks were stored at -80°C ± 15°C prior to thawing, dilution, and preparation of admixture containing 3.3 log_10_ plaque-forming units (PFU) of each serotype. The final admixture provided a final dose of 3 log_10_ PFU per 0.5 mL dose. Final potency titers were confirmed after preparation. Diluent (qualified Leibovitz L-15 medium) was used as placebo.

### Clinical procedures

Subjects were randomly selected to receive 0.5 mL of vaccine (n = 41) or placebo (n = 17), administered subcutaneously on day 0. Subjects were then evaluated as outpatients with clinical assessments and examinations every other day for the first 16 days and then on day 21, 28, 56 and 90. Symptoms and oral temperatures were also recorded by the subjects. On day 150, subjects were re-evaluated with inclusion and exclusion criteria, and then received a second dose of vaccine or placebo on day 180. The follow-up schedule and subject self-recording of temperature and symptoms was the same as dose one. Blood for safety laboratory studies, including virus titration, was drawn up to 60 days before dosing, and on days 0, 8, 10, 12, 14, 16, 21, and 28 relative to both first and second dosing. Blood for assay of neutralizing antibody titer was drawn on days 0, 28, 56, 90, 150, and 180 relative to both first and second dosing (**Fig 1**).

### Adverse events

Adverse events were recorded and classified based on intensity (mild, moderate, severe) and association to vaccination, as described [25]. Dengue vaccine-like rash was defined as a maculopapular rash, typically found on the trunk and proximal extremities, and most frequently observed 10–16 days after vaccination. Standard toxicity tables (https://www.fda.gov/downloads/BiologicsBloodVaccines/ucm091977 were used to grade and report abnormal clinical laboratory data.

### Laboratory assays and definitions

A subject was determined to have been infected by the vaccine if any DENV serotype was detected in blood or the subject seroconverted to any DENV serotype (as below) at any time point post-vaccination. A ‘dengue-like illness’ was defined as infection with fever and ≥ 2 symptoms of moderate intensity lasting ≥ 12 hours (i.e. headache, photophobia, myalgias, arthralgias). For detection of viremia, amplification and direct titration of DENV on vero cell monolayers was performed using serotype-specific monoclonal antibodies [19, 32]. Neutralizing antibodies to DENV were determined by plaque-reduction neutralization titer (PRNT) assays as described [32]. The reciprocal of the lowest calculated dilution which reduced virus by 50% (PRNT_50_) was reported as the neutralizing titer. Seropositivity was defined as a PRNT_50_ ≥1:10 at any time point up to and including day 90 post-vaccination. Seroconversion is defined as a four-fold rise in PRNT from day 0. For those who were seronegative to DENV at Day 0 (PRNT_50_ ≤ 1:5), a titer of 1:2.5 was assigned as the starting titer and thus a titer of ≥ 1:10 indicated seroconversion. An antibody “boost” was defined as a ≥4-fold rise in serum neutralization antibody titer comparing antibody titer at time of second dose (day 180) to peak titers up to day 270 post-vaccination.

### Data and statistical analysis

A per-protocol analysis was performed, although safety was evaluated for all subjects receiving vaccine. For analysis of adverse events, demographics, and differences in percentage of responders, Fisher’s Exact test was used. Wilcoxon rank-sum analysis was used to compare peak titers (only data from seropositive subjects was included post-vaccination, however once a subject became seropositive, titers from that subject were included in all subsequent analyses). Chi-square exact test of proportions was used to test seropositivity frequencies. For comparison of the responses to TV003 in subjects who were flavivirus-naïve at the time of vaccination, we used data collected from two previous cohorts (CIR268, n = 20 and CIR279, n = 38) [19]. With regard to clinical safety assessment procedures, assessment of viremia, and laboratory assay procedures, these studies are identical to the one described here. Within the flavivirus-naïve study cohort, there was one minor difference in a single timepoint in a subset of the subjects studied. For the CIR268 subjects, serum for neutralizing antibody was collected at Day 0, 28, 56, and 180 following dosing; for CIR279 subjects, neutralizing antibody was collected at Day 0, 28, 56, 90, and 180 following dosing (identical to this study). Statistical software packages SAS version 9.3 (SAS institute, Cary, North Carolina) were used.

## Results

### Demographic characteristics and study enrollment

One-hundred and fourteen subjects were assessed for eligibility, which included evidence of prior flavivirus exposure by serology for neutralizing antibodies against DENV1-4, yellow fever virus (YFV), West Nile virus (WNV), St. Louis encephalitis virus (SLEV), or Japanese encephalitis virus (JEV) or by documentation of YFV or JEV vaccination. Subjects were also eligible based on documented previous participation in a clinical trial of an experimental monovalent DENV vaccine–rDENV1Δ30 [29], rDEN2/4Δ30 [30], rDENV3Δ30,31 [28], or rDENV4Δ30 [31]. (See **Supporting information** for clinical protocol and CONSORT 2010 checklist). Fifty-eight subjects were enrolled and dosed (**Fig 1**). Of these, 25 (43.1%) were Black/African American, 29 (50%) were White, 2 (3.4%) were Asian and 2 (3.4%) self-reported as more than one racial category. Subjects were a median of 30 years of age, (range 20–50 years of age), with 46% males. Of the 58 subjects receiving a first dose, 41 received vaccine and 17 received placebo. There was no difference in median age or gender between vaccinees and placebo recipients.

Forty-eight subjects had evidence of antibodies to yellow fever virus with sixteen presenting evidence of vaccination. Fifteen subjects had received a dose of monovalent candidate dengue vaccine. No subjects had received a JEV vaccine. One, five, and six subjects had antibodies to JEV, WNV, SLEV, respectively. Seven subjects had an indication of prior exposure to more than one flavivirus (**S1 Table**). Between the first and second dose, ten subjects were withdrawn or removed from the study: one subject was removed due to pregnancy, one subject was incarcerated, three subjects moved out of the area, one subject was non-compliant with the study protocol, one subject was unavailable for a second dose, one subject had traveled to a dengue-endemic area in the interim, one subject had a previously undisclosed baseline medical issue (anaphylactic reaction), and one was unavailable to attend follow-up visits. Safety information was collected on all 58 subjects receiving a first dose (n = 41 for TV003 and n = 17 for placebo), but due to timing of study withdrawals, full immunogenicity data was collected on 55 of these (n = 38 for TV003, and n = 15 for placebo). Forty-eight subjects received a second dose (n = 33 for TV003 and n = 15 placebo). All but two subjects (one due to pregnancy and one due to moving out of study area) were followed throughout the remainder of the study. However, due to timing of removal from the study, full safety and immunogenicity data was collected on all subjects receiving a second dose.

### Safety and reactogenicity

Overall, both doses were well tolerated. Adverse events following the first dose are presented in **Table 1**. Rash was the only adverse event that occurred significantly more often in vaccine vs. placebo recipients (*P* < 0.001). Twenty-seven vaccinees (66%) experienced a dengue vaccine-like rash following the first dose. The rash was mild in 22/27 (81%) vaccines. Due to additional pruritus, rash was designated as moderate in 5/27 (18.5%) of vaccinees. No volunteer required medication for the pruritus. One placebo-recipient developed a transient rash similar to the dengue-vaccine-like rash.

**Table 1:** Adverse events following first and second dose of TV003 in flavivirus-experienced individuals.

		Dose 1			Dose 2	
Adverse event n, (%)	TV003 (n = 41)	Placebo (n = 17)	*P*-*value*^1^	TV003 (n = 33)	Placebo (n = 15)	*P value*^1^
**Injection site:**						
Erythema	1 (2)	0	1	0	0	n/a
Pain	1 (2)	0	1	1 (3)	2 (13)	0.23
Tenderness	3 (7)	1 (6)	1	0	0	n/a
Induration	0	0	n/a	0	0	n/a
**Systemic:**						
Fever	2 (5)	0	1	0	0	n/a
Headache	18 (44)	7 (41)	1	10(30)	6 (40)	0.53
** Rash**	27 (66)	1 (6)^2^	**<0.001**	0	0	n/a
Neutropenia	4 (10)	0	0.31	0	0	n/a
Elevated ALT	1 (2)	0	1	0	1 (7)	0.31
Myalgia	2 (5)	0	1	1 (3)	1(7)	0.53
Arthralgia	3 (7)	0	0.54	0	0	n/a
Retro-orbital pain	2 (5)	1 (6)	1	1 (3)	0	1
Fatigue	6 (15)	8 (47)	0.02^3^	4 (12)	3 (20)	0.66
Nausea	6 (15)	4 (24)	0.42	3 (9)	1 (7)	1
Prolonged PT	2 (5)	1 (6)	1	1(3)	0	1
Prolonged PTT	2 (45)	0	1	1(3)	0	1
Thrombocytopenia	0	0	n/a	1(3)	0	1

^1^Fisher’s exact test *P-*value for comparing placebo and TV003 recipients.

^2 ^Rash lasted one day.

^3^Not significant compared to placebo when adjusted for multiple comparisons by Bonferroni correction.

PT, prothrombin time; PTT, partial thromboplastin time.

There were two serious adverse events reported, both in the same vaccine recipient and both assessed as unrelated to vaccination. The first severe adverse event was syncope, occurring 51 days post-vaccination, and the second was a transient ischemic attack 77 days post-first vaccination. Four vaccinees developed short-lived neutropenia: two cases were mild in severity (absolute neutrophil count [ANC] of 880/mm^3^ on day 10 and 870/mm^3^ on day 16, respectively) and two were moderate (ANC nadir of 718/mm^3^ on day 8 and 704/mm^3^ on day 16, respectively). Three of those four subjects identified as Black/African American and all had relatively low ANC pre-vaccination (1,940/mm^3^ and 1,010/mm^3^, 1256/mm^3^, 1056/mm^3^, respectively). Reference ANC range is 1,500–8,850/mm^3^ (University of Vermont Medical Center Laboratory/Johns Hopkins Medical Laboratories Services). Two subjects had an elevated temperature possibly related to vaccine; in one volunteer fever lasted one hour and in the other volunteer, fever lasted for one day. One of these subjects complained of a constellation of mild upper respiratory symptoms following vaccination beginning day 9 post-vaccination, which coincided with household illness. Fever to a maximum of 101.5°F was noted on day 12 but resolved the next day, and a nasal swab was negative for influenza and RSV.

### Vaccine virus replication

Following the first dose of TV003, vaccine virus was detected in 31 subjects (76%) in the first sixteen days of follow up. Serotype, titer, day of onset, and duration of vaccine viremia is shown in **Table 2**. Most viremia was short lived, with a mean duration of 1–3 days. Among the subjects with viremia, 18 (58%) had viremia due to one dengue serotype, 12 (39%) due to two serotypes, and one (3%) due to three serotypes. DENV-3 was the most commonly recovered; it was found in 22 subjects, 54% of all vaccinees. Mean peak titers did not differ significantly between serotypes, and ranged from 0.68 to 1.1 log_10_ PFU/mL. A maximum titer of 2.4 log_10_ PFU/mL for DENV-2 and DENV-3 were seen in one volunteer each. Notably, viremia was not observed in any volunteer following the second dose. When compared to flavivirus-naïve subjects who received TV003 [19], the peak viremia titers for DENV-1, 2 and 4 were not significantly higher in flavivirus experienced persons. DENV-3 (DEN3Δ30/31) mean peak titers, although still low, were higher in flavivirus-exposed individuals (0.97 log_10_ PFU/ml vs. 0.56 log_10_ PFU/mL, *P* = 0.04). Together these results show that TV003 induces low-level vaccine viremia in previously flavivirus-exposed vaccinees, to levels that are largely in line with the vaccine viremia induced by this vaccine in flavivirus-naïve subjects, with no indication of heightened reactogenicity compared to flavivirus-naïve vaccinees.

**Table 2:** Viremia following the first dose of TV003 in flavivirus-experienced subjects (n = 41)^a^.

Vaccine component	No. of subjects with viremia (%)	Mean peak virus titer ± SE (log_10_ PFU/mL)	Maximum viral titer (log_10_ PFU/mL)	Mean day of viremia onset [range]	Mean duration of viremia, days [range]
DENV-1	9 (22)	0.71 ± 0.1	1.5	9.3 [8–11]	2.8 [1–6]
DENV-2	3 (7)	1.13 ± 0.6	2.4	10.0 [8–11]	1.0 [1]
DENV-3	22 (54)	**0.97 ± 0.1**^**1**^	2.4	8.9 [7–14]	2.7 [1–6]
DENV-4	11(27)	0.68 ± 0.1	1.6	8.7 [7–14]	1.7 [1–3]
Total	31 (76%)^2^				

^a^Post-TV003 viremia data in flavivirus-experienced subjects are compared to flavivirus-naive subjects from ref. [19], and include both cohorts (CIR279 and CIR268) from that study.

^1^The peak titer of DEN3Δ30/31 vaccine virus recovered from flavivirus-experienced vaccinees was significantly higher (0.97 log_10_ PFU/ml vs. 0.56 log_10_ PFU/mL, *P* = 0.04) than that recovered from flavivirus-naïve vaccinees in a previous trial of TV003 (ref. [19]).

^2^Eighteen subjects had viremia with one serotype. Thirteen subjects had viremia with >1 serotype. DENV-1+DENV-2 (n = 2), DENV-1+DENV-3 (n = 2), DENV-2+DENV-4 (n = 1), DENV-3+DENV-4 (n = 7), DENV-1+ DENV-3+DENV-4 (n = 1).

Viremia was not observed in any volunteer after the second dose of TV003.

### Serologic response to DENV

**Fig 2:**
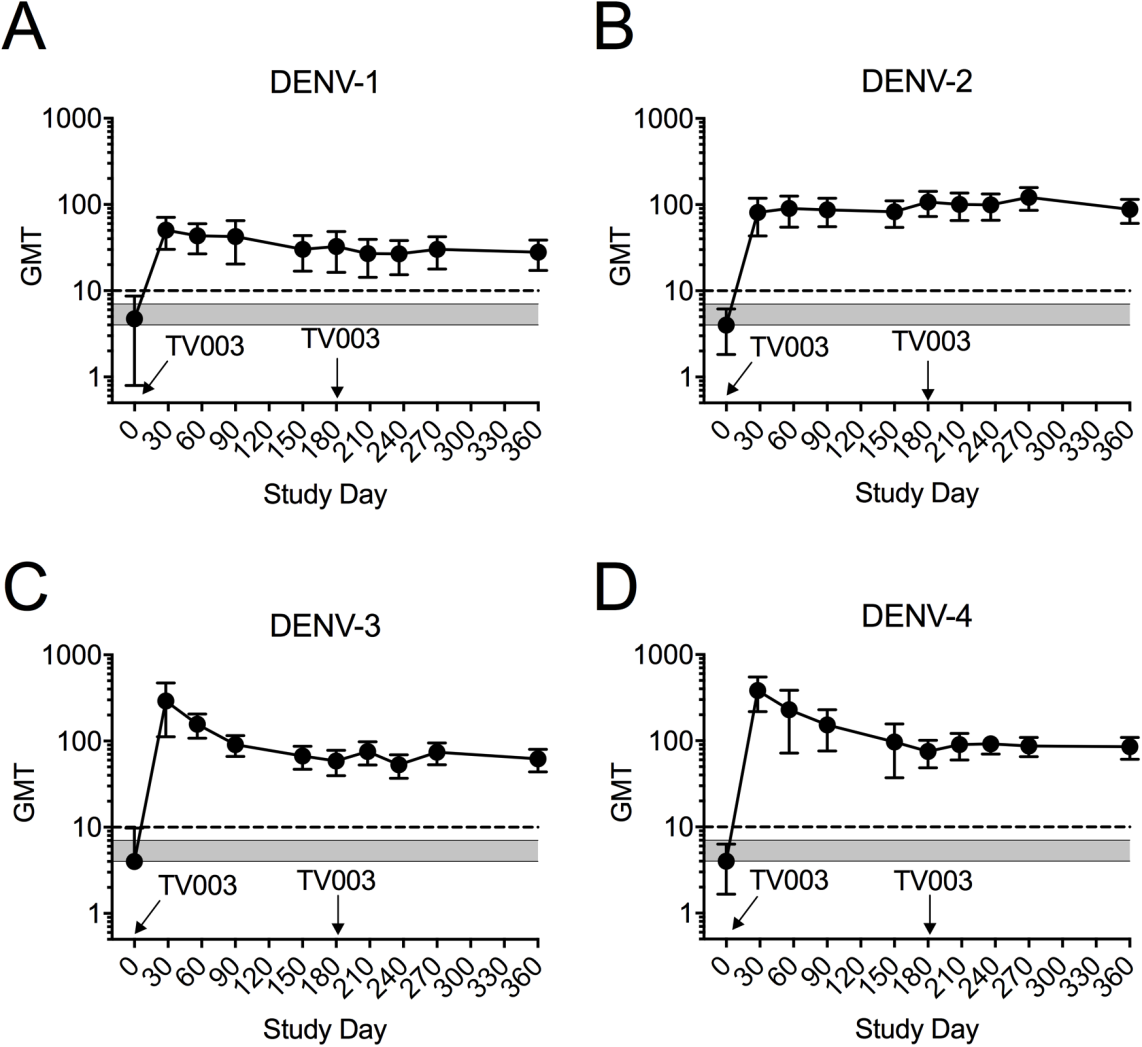
Mean plaque reduction (50%) neutralizing titers (PRNT_50_) for DENV-1 (**A**), DENV-2 (**B**), DENV-3 (**C**), and DENV-4 (**D**) following one or two doses of TV003 in flavivirus-experienced subjects (n = 41 for Dose 1, and n = 33 for Dose 2). All data represent mean titers (GMT) ± 95% confidence interval. Dotted line indicates seropositivity (PRNT_50_ ≥ 1:10) and gray area represents the range of Ab levels to each serotype observed in subjects who received placebo (n = 17 for dose 1 and n = 15 for Dose 2).

Neutralizing antibody responses for all serotypes were induced in flavivirus-experienced subjects following a single dose of TV003 (**Table 3**). The peak response for DENV-1 and DENV-2 neutralizing antibodies was broad across the first 90 days, whereas the highest DENV-3 and DENV-4 neutralizing antibody peaks were observed earlier (Day 28 after vaccination) (**Fig 2**). Following the second dose, there was no significant change in the mean peak antibody titers for any serotype compared to the pre-second dose titer (day 180) either in terms of magnitude (**Table 3**) or duration (**Fig 2**).

**Table 3:** Mean peak neutralizing antibody titers to TV003 in subjects who were flavivirus-experienced or -naïve^a^ prior to vaccination.

		Reciprocal geometric mean titer [range]			
		DENV-1	DENV-2	DENV-3	DENV-4
Dose 1 peak^1^	FV-experienced (n = 38)	56 [14–346]	**103** [16–584]	**171** [12–2841]	**252** [1111–3076]
	FV-naïve (n = 58)^a^	76 [11–433]	45 [10–239]	65 [11–469]	124 [43–508]
Dose 2 peak^2^	FV-experienced (n = 33)	38 [12–150]	**107** [14–426]	79 [16–244]	100 [17–348]
	FV-naïve (n = 44)^a^	41 [11–769]	34 [11–133]	63 [17–842]	78 [12–575]

^1^Mean peak neutralizing titer after Dose 1 of TV003 (measured between days 0–90 after vaccination) administered to subjects who were flavivirus-exposed versus flavivirus–naïve at Day 0: DENV-1 (*P* = 0.09), DENV-2 (*P* = 0.001), DENV-3 (*P* = 0.0003) DENV-4 (*P* = 0.004).

^2^Mean peak neutralizing antibody titer after Dose 2 of TV003 (measured between days 0–90 after second dose, i.e. study days 180–270) administered to subjects who were flavivirus-exposed versus flavivirus–naïve at Day 0: DENV-1 (*P* = 0.52), DENV-2 (*P* <0.0001), DENV-3 (*P* = 0.2), DENV-4 (*P* = 0.16).

Significant values (*P* <0.03 level after adjusting for multiple comparisons) in **BOLD**.

^a^TV003 vaccine response data in flavivirus-naive subjects are from ref. [19], and include both cohorts (CIR279 and CIR268) from that study.

There were eleven subjects who were seropositive (PRNT_50_ >1:10) on day 0: DENV-1 (n = 4), DENV-2 (n = 1), DENV-3 (n = 3) and DENV-4 (n = 3), however reciprocal PRNT_50_ values were 10–20, indicating low level of positivity.

We also compared the neutralizing antibody responses of TV003 vaccinees with prior flavivirus exposure to those in subjects from prior studies of TV003 [19] who were flavivirus-naïve at the time of vaccination. As seen in **Table 3,** the antibody responses for DENV-1 (*P* = 0.09), DENV-2 (*P* = 0.001), DENV-3 (*P* = 0.0003), and DENV-4 (*P* = 0.004) following dose 1 of TV003 were significantly higher in flavivirus-exposed subjects even after adjustment for multiple comparisons. DENV-2 neutralizing antibodies remained elevated (*P* < 0.0001) post dose 2 of TV003 in flavivirus-exposed subjects compared to those who were naïve at the start of the trial.

We next assessed mean neutralizing antibody titers to DENV-1 to -4 six months following one or two doses of TV003. Six months after one dose of TV003, mean neutralizing antibody titers to DENV-2 were higher in flavivirus-experienced vaccinees compared to flavivirus -naïve vaccines (**Table 4**). DENV-2 (*P* < 0.0001), DENV-3 (*P* = 0.02), and DENV-4 (*P* = 0.01), but not DENV-1 (*P* = 0.56), antibody responses were higher 6 months after a second dose of TV003 in flavivirus-experienced vaccinees compared to flavivirus -naïve vaccinees (**Table 4**).

**Table 4:** Reciprocal mean neutralizing antibody titers 6 months after dose 1 (Day 180) and dose 2 (Day 360) after vaccination with TV003 of subjects who were either flavivirus-experienced or–naïve^a^ at time of first vaccination.

		Reciprocal geometric mean titer [range]			
		DENV-1	DENV-2	DENV-3	DENV-4
Day 180^1^	FV-experienced (n = 38)	**33** [11–275]	**108** [20–380]	59 [11–248]	75 [11–430]
	FV-naive (n = 58)^a^	22 [11–310]	24 [11–219]	43 [10–228]	55 [14–243]
Day 360^2^	FV-experienced (n = 33)	29 [10–117]	**89** [14–530]	**62** [11–229]	**85** [11–293]
	FV-naive^a^ (n = 44)^a^	28 [10–342]	29 [10–171]	31 [11–279]	36 [11–185]

^1^Comparison of mean neutralizing antibody titer at Day 180 (6 months after dose 1) post-TV003) between subjects who were flavivirus-exposed versus flavivirus–naïve at Day 0: DENV-1 (*P* = 0.03), DENV-2 (*P* <0.0001), DENV-3 (*P* = 0.93), DENV-4 (*P* = 0.56).

^2^Comparison of mean neutralizing antibody titer at Day 360 (6 months after dose 2) post-TV003) between subjects who were flavivirus-exposed versus flavivirus–naïve at Day 0: DENV-1 (*P* = 0.56), DENV-2 (*P* <0.0001), DENV-3 (*P* = 0.02), DENV-4 (*P* = 0.01).

Significant values in **BOLD** (At *P* < 0.05 level after adjusting for multiple comparisons).

^a^TV003 vaccine response data in flavivirus-naive subjects are from ref. [19], and include both cohorts (CIR279 and CIR268) from that study.

In our enrolled flavivirus-experienced cohort there were 29 subjects with prior yellow fever exposure either serologically (n = 17) or via documentation of YFV vaccination (n = 12) assigned to the TV003 arm. Eleven subjects allocated to the TV003 arm had prior dengue exposure (either serologically, n = 3, or via participation in a previous monovalent DENV vaccine trial, n = 8) [28–31]. There was no difference in type of flavivirus exposure across treatment arms (**S1 Table**). There was no difference in DENV viremia or vaccine reactogenicity (**S2 Table**) or DENV peak immunogenicity (**S1 Fig**) after a single dose of TV003 depending on method of establishing prior flavivirus-exposures (serology versus vaccination documentation). Furthermore, among those who received TV003, there were thirty-five subjects with a single flavivirus exposure and six with more than one exposure. There were also no differences in TV003-induced DENV viremia or reactogenicity (**S2 Table**) or DENV immunogenicity (**S1 Fig**) depending on whether the prior flavivirus exposure that met inclusion criteria was due to single or multiple different flavivirus exposures.

**Fig 3:**
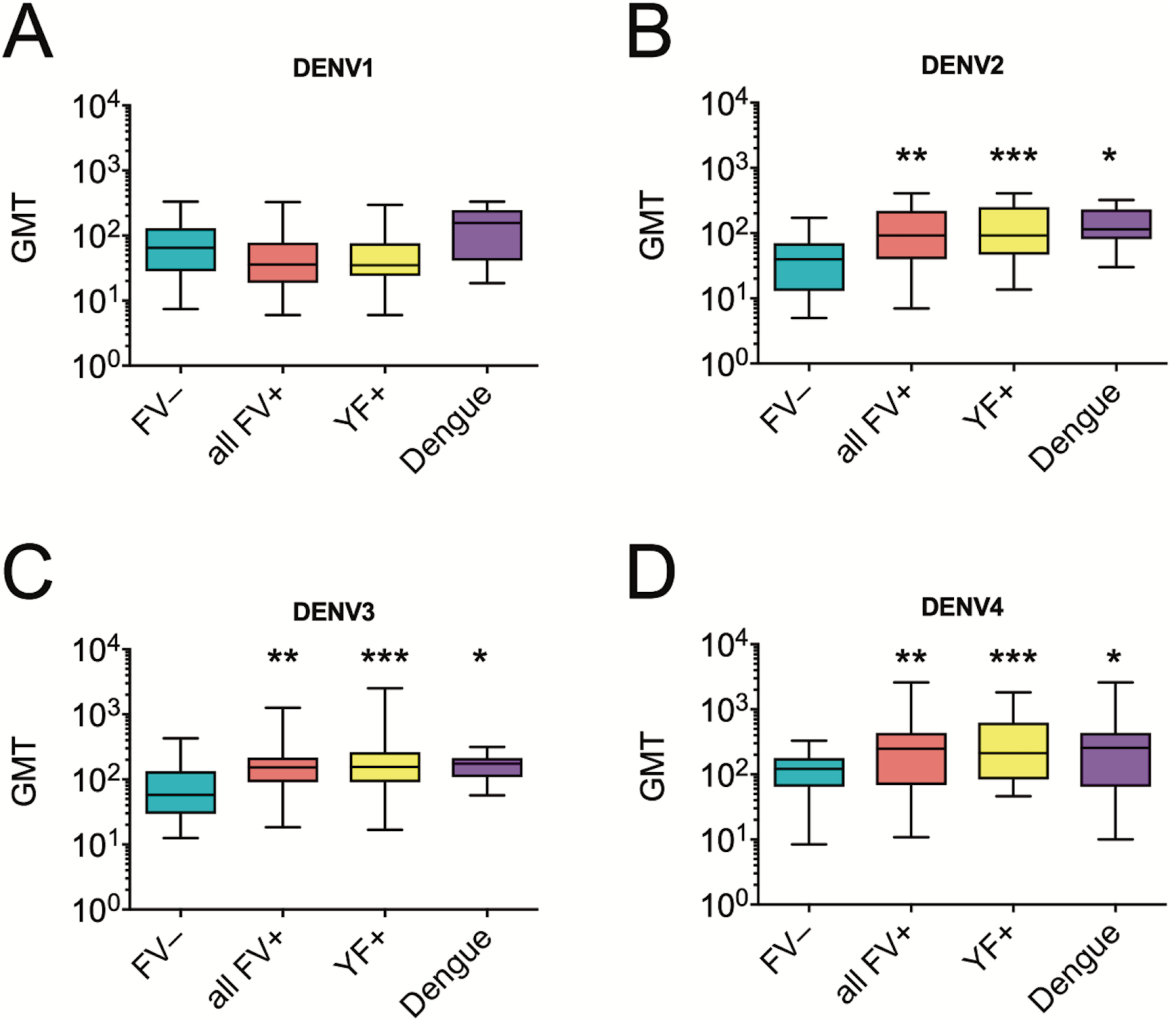
Mean peak neutralizing antibody titers (PRNT_50_) for DENV-1(**A**), DENV-2 (**B**), DENV-3 (**C**), and DENV-4 (**D)** up to 90 days following one dose of TV003 in flavivirus-naïve subjects (n = 58, from ref. [19]), all flavivirus-experienced (all FV+) subjects (n = 38) and by two subgroups–those with yellow fever virus (YF+, n = 31) or dengue virus exposure (Dengue, n = 11). All data represent mean titers (GMT) ± 95% confidence interval. Statistical significance of the difference between FV-naïve group was assessed by one-way ANOVA with Sidak correction for multiple comparisons. *, *P* < 0.05, **, *P* < 0.01, *** *P*, < 0.001.

Anamnestic antibody responses to YFV, JEV, or DENV vaccine have been observed in subjects with a heterologous flavivirus exposure prior to vaccination [33–36]. In addition, the CYD-TDV tetravalent dengue vaccine protected against dengue disease only those individuals who were DENV-primed prior to vaccination [16, 17]. We, thus, investigated whether prior YFV or dengue virus exposure had an impact on mean peak neutralizing antibody levels after one dose of TV003. We found that YFV or DENV exposure prior to vaccination with TV003 were associated with significantly higher neutralizing antibody titers to DENV-2, DENV-3, and DENV-4 following a single dose of TV003 (**Fig 3**).

We also assessed overall seropositivity to each DENV serotype after one or two doses of TV003 as a function of flavivirus exposure status at the time of first receiving TV003. In line with prior results for this vaccine in flavivirus-naïve subjects [19–21, 25, 28], TV003 vaccination led to over to 89% seroconversion to all DENV serotypes in flavivirus-experienced subjects. In particular, prior flavivirus exposure was associated with a sustained effect on DENV-2 seropositivity following vaccination with TV003 (**Table 5**).

**Table 5:** Frequency of seropositivity to each DENV serotype after one or two doses of TV003 in subjects who were flavivirus-experienced or -naïve^a^ prior to vaccination.

Seropositivity		DENV-1	DENV-2	DENV-3	DENV-4
Dose 1	FV-experienced (n = 38)	89%	**95%**^**1**^	97%	100%
	FV-naïve (n = 58)^a^	95%	67%	98%	100%
Dose 2	FV-experienced (n = 33)	82%	**97%**^**2**^	94%	100%
	FV-naïve (n = 44)^a^	75%	70%	91%	98%

Comparison of cumulative seropositivity for each DENV serotype post-TV003 between subjects who were flavivirus-exposed versus flavivirus–naïve at Day 0.

^1^ Dose 1 unadjusted *P*-value = 0.0019.

^2^ Dose 2 unadjusted *P*-value = 0.0057.

Significant values in **BOLD** (At *P* < 0.03 level after adjusting for multiple comparisons) by Chi-square exact test of proportions.

^a^TV003 vaccine response data in flavivirus-naive subjects are from ref. [19], and include both cohorts (CIR279 and CIR268) from that study.

TV003 has been shown to elicit neutralizing antibodies to each DENV serotype in flavivirus-naive vaccinees [19]. We, therefore, assessed the valency of DENV-specific antibody responses after TV003 vaccination in previously flavivirus-exposed subjects. Overall, 87% of flavivirus-experienced subjects achieved a tetravalent antibody response and an additional 10% achieved a trivalent response to vaccination following a single dose of TV003 (**Table 6**). We further found that flavivirus exposure prior to vaccination with TV003 was significantly associated with an increased the proportion of subjects with a tetravalent response as compared the proportion of tetravalent responders who were flavivirus-naïve [19] prior to receiving TV003 (*P* = 0.013).

**Table 6:** Valency of DENV responses after one or two doses of TV003 in subjects who were flavivirus-experienced or -naïve^a^ prior to vaccination.

		Subjects with antibody response by valence, %, (cumulative)			
		Tetra	Tri	Bi	Mono
Dose 1^1^	FV-experienced (n = 38)	**87**	10 (97)	0 (100)	3 (100)
	FV-naïve (n = 58)^a^	66	29 (95)	5 (100)	0 (100)
Dose 2^2^	FV-experienced (n = 33)	**79**	18 (97)	0 (100)	3 (100)
	FV-naïve (n = 44)^a^	48	39 (87)	14 (100)	0 (100)

Frequency of tetravalent responses after 1 or 2 doses of TV003 (measured between days 0–180 after dosing) administered to subjects who were flavivirus-exposed versus flavivirus–naïve prior to vaccination at Day 0. **BOLD** indicates statistical significance between FV-experienced versus FV-naïve by Chi-square exact test of proportions.

^1^Dose 1 of TV003 (87% vs. 66%, *P* = 0.02)

^2^Dose 2 (79% vs. 48%, P = 0.006), respectively.

^a^TV003 vaccine response data in flavivirus-naive subjects are from ref. [19], and include both cohorts (CIR279 and CIR268) from that study.

## Discussion

Here we assessed the safety and immunogenicity of the NIH live-attenuated tetravalent dengue vaccine TV003 in a randomized controlled trial of healthy adult subjects with known previous exposure to flaviviruses. Subjects had evidence of exposure to DENV, YFV, WNV, SLEV, or JEV. Flavivirus-experienced vaccinees received two doses of TV003 or placebo, at a six-month interval. The TV003 vaccine was well tolerated and broadly immunogenic against all four viral serotypes, leading to an 87% tetravalent response after a single dose. As previously demonstrated for flavivirus-naïve vaccinees [19, 25, 27], the second dose of TV003 did not affect overall immunogenicity, and is likely unnecessary for optimal effectiveness of the vaccine. Adverse events or post-vaccine reactogenicity to TV003 were not different in flavivirus-experienced subjects when compared to flavivirus-naïve subjects evaluated in previous studies of this vaccine [19, 25, 28]. As anticipated, the only significant event when comparing vaccinees and placebo recipients was a self-resolving rash in most (66%) subjects following the first, but not second, dose of TV003. The protocol-defined criteria for dengue-like illness was not met in any study subject.

As previously reported, following first vaccination with TV003, three-quarters of flavivirus-naive subjects experienced viremia following the first vaccination and none experienced viremia following the second dose [19, 28]. Here, in subjects who were flavivirus-exposed prior to vaccination with TV003, we also observed that 76% of subjects became viremic. The incidence of TV003-induced viremia did not depend on type of flavivirus exposure (**S2 Table**). Although vaccine-induced viremia generally leads to the development of protective and sterilizing immunity, it is not strictly correlated with immunogenicity. When directly compared to flavivirus-naïve vaccinees from a recent trial of TV003 [19] virus titers for all DENV serotypes trended higher in flavivirus-exposed vaccinees, but only the DENV-3 mean peak viral titer was significantly elevated in flavivirus-exposed subjects (Table 2). It is, therefore, possible that pre-existing flavivirus antibodies may play a minor role in transiently enhancing DENV vaccine viremia on a serotype-specific basis. A recent report suggested that pre-existing antibodies to JEV may transiently increase YFV viremia after YFV vaccination [36]. CYD-TDV viremia was not enhanced by prior YFV exposure, but overall CYD-TDV infectivity in this study was very low [37]. Importantly, DENV vaccine viremia was not associated with any increased reactogenicity compared to that which has been observed in flavivirus-naïve vaccinees. The mean day of onset and mean duration of viremia for all serotypes in response to TV003 were also not different between serotypes or compared to findings from TV003-vaccinated naïve subjects. In all, the levels of DENV viremia we observed in flavivirus-exposed or–naïve TV003-vaccinated subjects were on the order of 0.7 to 1.1 log_10_ PFU/mL as assessed by culture of live virus. In natural dengue, some estimates of viremia during disease have been based on mosquito infectious doses or genome equivalents, suggest viremia levels in excess of 7–8 logs [10, 11]. Thus, with the noted caveat of differing methods, this suggests that the level of viremia seen after TV003 is relatively low and is not globally affected by flavivirus-status. Nevertheless, for live-attenuated DENV vaccination, viremia itself appears to be one of several indicators of infection, along with immune response, and clinical signs such as dengue vaccine-like rash. Although the exact correlation/interaction of these signals is still unknown, vaccine infectivity remains the principal indicator that a live vaccine is performing as desired.

After the first dose of TV003, neutralizing antibody responses (PRNT_50_) to DENV-2, -3, and -4 were higher than those previously observed in individuals who were flavivirus-naïve at the time of first TV003 administration [19]. At present, the mechanism of flavivirus anamnestic responses to TV003 is unknown, but is under investigation. It is not clear why post-vaccination DENV-1 antibody titers were not affected by prior flavivirus experience. Seroconversion to DENV1 after CYD-TDV vaccination was delayed compared to DENV-2, -3, and -4 seroconversion in flavivirus-primed subjects [37]. As expected from studies of flavivirus-naive TV003 vaccinees [19, 25, 28], mean peak antibody titers post TV003 in flavivirus-experienced subjects gradually declined during the six months of follow-up to a resting level higher than observed at Day 0 (**Fig 2**). However, a second dose of TV003 failed to induce a significant increase in neutralizing antibodies to DENV serotype in flavivirus-experienced subjects. In line with this result, a second dose of TV003 also failed to increase neutralizing antibodies in subjects who were flavivirus-naïve at the time of first vaccination [19, 25, 27]. Lack of a clear boost (4-fold or greater) after the second dose strongly suggests the potential of TV003 as a single dose vaccine since it appears that immunity raised to the first dose may provide sterilizing immunity against infection with the second dose.

Results from recent trials of CYD-TDV showed significant efficacy only in subjects with DENV exposure prior to vaccination with CYD-TDV [15, 16, 38]. However other flaviviruses such as YFV or Zika virus (ZIKV) share the same vectors as DENV, often co-circulate, and, in the case of YFV in the Americas, there is widespread vaccination. Among the flavivirus exposures in the flavivirus-experienced vaccine cohort we studied here, YFV or DENV exposures comprised the largest fractions. This offered an opportunity to examine whether DENV exposure *per se* was a determining factor to account for the increased immunogenicity after TV003 in the flavivirus-exposed cohort as compared to flavivirus-naïve TV003 vaccine cohort. In agreement with other results [37, 39], we found that vaccination of DENV-exposed subjects with a live attenuated tetravalent dengue vaccine promoted increased DENV neutralizing antibody titers as compared to flavivirus-naïve vaccinees. Interestingly, we also found that YFV-exposed subjects exhibited peak neutralizing antibody titers to TV003 which were similar to those in the DENV-exposed vaccinees. Thus, our results with TV003 are consistent with a priming effect of YFV exposure on neutralizing antibody responses elicited by live monovalent DENV2 [35] and live attenuated tetravalent CYD-TDV vaccines [37]. Since CYD-TDV is based on the YFV genetic backbone whereas TV003 contains only DENV genome, it is possible that different mechanisms for YFV/DENV anamnestic responses are involved. Indeed, TV003 induced a balanced neutralizing response across serotypes in flavivirus-exposed subjects, which is consistent with our results in flavivirus-naïve vaccinees [19, 25, 26]. Taken together with the efficacy data for CYD-TDV, our results support the notion that prior flavivirus exposure contributes to an anamnestic response to live attenuated tetravalent dengue vaccination.

Protective DENV immunity, whether through vaccination or natural infection is thought to be comprised of several factors including neutralizing antibody as well as activation of CD8+ and CD4+ T cells [40–42]. Recently we have learned that CD8+ T cells predominantly target conserved nonstructural (NS) elements of DENV in natural infection and after immunization with the live attenuated DENV vaccine TV003 [43, 44]. Similarly, CD4+ T cells target predominantly the Capsid and NS3 and NS5 [42]. Nonstructural genes are more conserved among flaviviruses than are structural genes [45]. Thus, mechanistically, it is possible that pan-flavivirus memory T cells expected to be present in flavivirus-experienced individuals may contribute to increased immune response to TV003 compared to flavivirus-naïve individuals that lack such T cell memory.

This study has several limitations, including the fact that it was not performed in a flavivirus-endemic region. Most exposures were due to either YFV or DENV with less representation from other flaviviruses and no exposures to TBEV. We did not have capabilities at the time of this study to test for Zika virus, as the study was conducted well before the Zika virus outbreak of 2015–2016 in the Americas. In addition, comparison of neutralizing antibody titers from this cohort to the previously studied (flavivirus-naïve) subjects [19] is imperfect. We note, however, that recruitment procedures, study demographics, and retention rates were comparable to those for prior trials of TV003 in flavivirus-naïve subjects [19, 25, 28] (**S3 Table**). Although the core assays (PRNT_50_) were performed in the same manner, they were not performed concurrently for the two studies. Another limitation is that for twenty of the flavivirus-naïve vaccinees studied (out of a total of 58) there was no day 90 timepoint for assessment of neutralizing antibody titer. However, we found cumulatively that 91% of flavivirus-naïve vaccinees who had neutralizing antibody testing through day 90 had exhibited peak antibody levels by day 56 (**S4 Table**). Furthermore, although neutralizing antibodies are the currently accepted readout of dengue vaccine immunogenicity, it is not known whether this measure *per se* is sufficient to predict protection against clinical dengue disease. In several subjects, multiple flavivirus antibodies (likely cross-reactive) were seen pre-dosing and the original flavivirus infection could not be determined.

In summary, this work demonstrates the tolerability and immunogenicity of the single-dose NIH TV003 vaccine in flavivirus-experienced subjects and adds to the existing safety record of this vaccine in flavivirus-naïve adults. In flavivirus-experienced subjects we found limited reactogenicity to TV003, which is in line with previous results obtained for this vaccine in flavivirus-naïve subjects who received TV003. We found higher DENV-3 vaccine viremia after administration of TV003 to flavivirus-experienced subjects as compared to flavivirus-naïve subjects; however, levels of vaccine viral replication remained low in all subjects. The live attenuated dengue vaccine TV003 triggered higher neutralizing antibody titers (in three of four serotypes) in flavivirus-experienced subjects compared to levels observed following administration of TV003 in flavivirus-naive subjects studied previously [19]. These data have informed the clinical development of TV003 and have contributed to the advancement of this live attenuated dengue vaccine candidate to Phase 2 and 3 studies currently underway in dengue-endemic Thailand and Brazil, respectively.

## Supporting information

S1 Table Flavivirus exposures in randomly assigned treatment arms. (DOCX)

S2 Table Incidence and mean peak of DENV1-4 viremia and incidence of dengue vaccine-like rash after one dose of TV003, based on number of prior flavivirus exposures, type of exposure, and method of verification (serology or documentation). (DOCX)

S3 Table Study retention/completion of vaccine studies. (DOCX)

S4 Table Incidence of peak DENV PRNT_50_ titer which occurred prior to Day 90 after administration of TV003 to flavivirus-naïve subjects. (DOCX)

S1 Protocol Clinical trial protocol for study CIR 280. (PDF)

S1 Checklist Consort 2010 checklist for CIR 280. (PDF)

S1 Fig Mean peak neutralizing antibody titer to DENV-1 (A), -2 (B), -3 (C), and -4 (D) after one dose of TV003 to flavivirus-exposed subjects.
(DOCX)
