# Supplementary material for: In a randomized trial, the live attenuated tetravalent dengue vaccine TV003 is well-tolerated and highly immunogenic in subjects with flavivirus exposure prior to vaccination
Source: PLoS Negl Trop Dis. 2017 May 8;11(5):e0005584. doi: 10.1371/journal.pntd.0005584 (PMC5436874; doi:10.1371/journal.pntd.0005584)
Supplement: S1 Table — (DOCX) [file pntd.0005584.s001.docx]

**Table S1: Flavivirus exposures in randomly assigned treatment arms**

| Assignments | TV003, n | PLACEBO, n | *P*-value^a^ |
| --- | --- | --- | --- |
| Single | 35 | 16 | 0.66^a^ |
| ≥ 2 | 6 | 1 |  |
| DENV | 11 | 8 | 0.2^b^ |
| YFV | 29 | 10 |  |
| WNV/SLEV/JEV (combined) | 8 | 1 |  |

^a^Fisher’s exact test, ^b^Chi-square.
