# Supplementary material for: In a randomized trial, the live attenuated tetravalent dengue vaccine TV003 is well-tolerated and highly immunogenic in subjects with flavivirus exposure prior to vaccination
Source: PLoS Negl Trop Dis. 2017 May 8;11(5):e0005584. doi: 10.1371/journal.pntd.0005584 (PMC5436874; doi:10.1371/journal.pntd.0005584)
Supplement: S2 Table — (DOCX) [file pntd.0005584.s002.docx]

**Table S2. Incidence and mean peak of DENV1-4 viremia and incidence of dengue vaccine-like rash after one dose of TV003, based on number of prior flavivirus exposures, type of exposure, and method of verification (serology or documentation).**

| Assigned to TV003 | | Viremic  n, (%) | Mean peak  DENV1-4 viremia^a^ | Rash  n, (%) |
| --- | --- | --- | --- | --- |
| Number of FV exposures | Single (n = 35) | 28 (68%) | 0.79 ± 0.51 | 23 (66%) |
|  | ≥ 2 (n = 6) | 3 (50%) | 1.55 ± 0.95 | 4 (67%) |
| Type of FV exposure | YF (n = 29) | 24 (83%) | 0.83 ± 0.58 | 21 (72%) |
|  | DENV (n = 11) | 7 (64%) | 1.1 ± 0.80 | 7 (64%) |
|  | Other (n = 8) | 4 (50%) | 1.48 ± 0.83 | 4 (50%) |
| Method of validation | YF-doc (n = 12) | 12 (100%) | 0.56 ± 0.24 | 10 (83%) |
|  | YF-sero (n= 17) | 12 (71%) | 1.06 ± 0.63 | 11 (65%) |
|  | DENV-doc (n = 8) | 6 (75%) | 0.95 ± 0.71 | 4 (50%) |
|  | DENV-sero (n= 3) | 1 (33%) | 2.3 | 1 (33%) |

^a^log^10^ PFU/mL ± S.D.

Other = WNV, SLEV, JEV

No differences in viremia or rash incidence (Chi-square) or in peak viremia levels (one–way ANOVA) were detected amongst the groups.
