# Supplementary material for: In a randomized trial, the live attenuated tetravalent dengue vaccine TV003 is well-tolerated and highly immunogenic in subjects with flavivirus exposure prior to vaccination
Source: PLoS Negl Trop Dis. 2017 May 8;11(5):e0005584. doi: 10.1371/journal.pntd.0005584 (PMC5436874; doi:10.1371/journal.pntd.0005584)
Supplement: S3 Table — (DOCX) [file pntd.0005584.s003.docx]

**Table S3: Study retention/completion of vaccine studies.**

| **RECRUITMENT** | FV-naïve^a^ | | FV-experienced | |
| --- | --- | --- | --- | --- |
| White | 57.1% | | 50% | |
| African American | 39.3% | | 43.1% | |
| Non-white/AA races/ethnicities | 4.6% | | 3.4% | |
| Males | 52.4% | | 50% | |
| Median age | 29.3 | | 30 | |
| **RETENTION** | FV-naïve^a^ | | FV-experienced | |
| Study completion | 62/84 (74%) | | 48/58 (83%) | |
| *By treatment* | TV003 | PLACEBO | TV003 | PLACEBO |
| Dose 1 | 60 | 24 | 41 | 17 |
| Dose 2 | 44 (73%) | 18 (75%) | 33 (80%) | 15 (88%) |
| Study completion (safety) | 44 (73%) | 18 (75%) | 33 (80%) | 15 (100%) |
| Study completion (PRNT) | 44 (73%) | 18 (75%) | 33 (80%) | 15 (88%) |

^a^Data for flavivirus-naive study subjects are from ref. [[19](#_ENREF_19)], and include both cohorts (CIR279 and CIR268) from that study.
