# Supplementary material for: In a randomized trial, the live attenuated tetravalent dengue vaccine TV003 is well-tolerated and highly immunogenic in subjects with flavivirus exposure prior to vaccination
Source: PLoS Negl Trop Dis. 2017 May 8;11(5):e0005584. doi: 10.1371/journal.pntd.0005584 (PMC5436874; doi:10.1371/journal.pntd.0005584)
Supplement: S4 Table — (DOCX) [file pntd.0005584.s004.docx]

**Table S4:** Incidence of peak DENV PRNT_50_ titer which occurred prior to Day 90 after administration of TV003 to flavivirus-naïve^a^ subjects (n = 38).

| by serotype | | | | Cumulative |
| --- | --- | --- | --- | --- |
| DENV-1 | DENV-2 | DENV-3 | DENV-4 |  |
| 31/38 (82%) | 34/38 (89%) | 38/38 (100%) | 36/38 (95%) | 91% |

^a^Data for flavivirus-naive TV003-vaccinated subjects are from ref. [[19](#_ENREF_19)], and include only cohort CIR279 from that study.
