# Supplementary material for: In a randomized trial, the live attenuated tetravalent dengue vaccine TV003 is well-tolerated and highly immunogenic in subjects with flavivirus exposure prior to vaccination
Source: PLoS Negl Trop Dis. 2017 May 8;11(5):e0005584. doi: 10.1371/journal.pntd.0005584 (PMC5436874; doi:10.1371/journal.pntd.0005584)
Supplement: S1 Protocol — (PDF) [file pntd.0005584.s005.pdf]

A Phase 1 Evaluation of the Safety and Immunogenicity of the Recombinant Live Attenuated Tetravalent Dengue Virus Vaccine Admixtures TV003 and TV005 in Healthy Flavivirus-experienced Adult Subjects

CIR Protocol Number	280
WIRB Protocol Number	20120069
FDA IND Number	BB-IND 14268
Ionizing Radiation	None
Multi-institution	Yes
Project Assurance:	FWA# 00000287 (Johns Hopkins Bloomberg School of Public Health<br>FWA# 0000727 (Fletcher Allen Health Care)<br>FWA# 0000723 (University of Vermont)
Protocol Version:	4.0 FINAL
Date:	12 September 2013

Sponsored by:
Regulatory Compliance and Human Subjects Protection Branch (RCHSPB)
National Institute of Allergy and Infectious Diseases (NIAID)
National Institutes of Health (NIH)

The information contained herein is the property of the National Institute of Allergy and Infectious Diseases, National Institutes of Health and may not be reproduced, published, or disclosed to others without the written authorization of the National Institute of Allergy and Infectious Diseases.

Team Roster

Principal Investigator:

Anna Durbin, MD
Center for Immunization Research (CIR)
Johns Hopkins School of Public Health
624 N. Broadway, Baltimore, MD 21205
410-614-4736 (phone)
410-340-6852 (phone)

Co –Investigators

Beth Kirkpatrick, MD
University of Vermont, College of Medicine
University of Vermont
89 Beaumont Avenue
Given C205
Burlington, VT 05405

Clinical Investigators:

Kawsar Talaat, MD
Noreen A Hynes MD, MPH, DTM&H
Cecilia Tibery, PAC
Daniel Elwood, RN, CCRC
Beulah Sabundayo, Pharm D, MPH
Center for Immunization Research
Johns Hopkins School of Public Health
624 N. Broadway, Baltimore, MD 21205

Kristen Pierce, MD
Catherine Larsson, BA, CCRC
University of Vermont, College of Medicine
University of Vermont
89 Beaumont Avenue
Given C205
Burlington, VT 05405

Scientific Investigators:

Stephen S. Whitehead, PhD
Kanta Subbarao, MD
Laboratory of Infectious Diseases
NIAID, NIH
301-496-7692 (phone)
301-451-3839 (phone)

RCHSPB Regulatory Affairs:

John Tierney, RN, MPM
RCHSPB/NIAID/NIH
6700-B Rockledge Dr. Room #1133
MSC 7609 Bethesda, MD 20892
301-451-5136 (phone)

Sponsor Medical Monitor:

Barry Eagel, MD
Clinical Safety Director
CMRP/RCHSPP/NIAID/NIH
SAIC Frederick, Inc
5705 Industry Lane
Frederick, MD 21702-1201
301-846-7322 (phone)
RCHSPSafety@mail.nih.gov

Clinical Study Sites:

Center for Immunization Research
Johns Hopkins School of Public Health
624 N. Broadway, Baltimore, MD 21205
410-955-1622 (phone)

University of Vermont, College of Medicine
University of Vermont
89 Beaumont Avenue
Given C205
Burlington, VT 05405
and
Fletcher Allen Health Care (FAHC)
General Clinical Research Center (GCRC)
Baird 7
111 Colchester Avenue
Burlington, VT 05401

Research Laboratories:

Center for Immunization Research
Johns Hopkins School of Public Health
615 N. Wolfe St., Baltimore, MD 21205
410-955-7230 (phone)

University of Vermont, College of Medicine
University of Vermont
89 Beaumont Avenue
Given C205
Burlington, VT 05405

Clinical Laboratories:

Quest Diagnostics
1901 Sulphur Spring Road
Baltimore, MD 21227

Fletcher Allen Health Care (FAHC)
111 Colchester Avenue
Burlington, VT 05401

Table of Contents

TEAM ROSTER **2**
TABLE OF CONTENTS **4**
1 PROTOCOL SUMMARY **10**
2 INTRODUCTION **11**
2.1 Background – Dengue 11
2.2 Background - Dengue Vaccines 12
2.3 Vaccine Descriptions 13
2.3.1 rDEN1Δ30 Vaccine 13
2.3.1.1 Final Container rDEN1Δ30 .... *13*
2.3.1.2 Composition rDEN1Δ30 .... *13*
2.3.2 rDEN2/4Δ30(ME) Vaccine 14
2.3.2.1 Final Container rDEN2/4Δ30(ME) *14*
2.3.2.2 Composition rDEN2/4Δ30(ME) *15*
2.3.3 rDEN3Δ30/31-7164 16
2.3.3.1 Final Container of rDEN3Δ30/31-7164 Lot #M10377008 .... *16*
2.3.3.2 Composition of rDEN3Δ30/31-7164 Lot #M10377008 .... *16*
2.3.3.3 Final Container of rDEN3Δ30/31-7164 Lot DEN3#113B *17*
2.3.3.4 Composition of rDEN3Δ30/31-7164 Lot DEN3#113B *17*
2.3.4 rDEN4Δ30 Vaccine Lot# 109A 18
2.3.4.1 Final Container rDEN4Δ30 Lot# 109A *18*
2.3.4.2 Composition rDEN4Δ30 Lot#109A *18*
2.4 Rationale 19
2.4.1 Animal Experience 19
2.4.2 Clinical Experience with the recombinant live attenuated dengue vaccine candidates 20
2.4.2.1 Clinical experience with DENV-1, DENV-2, DENV-3, and DENV-4 monovalent vaccine candidates *21*
2.4.2.2 Summary of clinical, virologic, and serologic response to monovalent candidates *22*
2.4.2.3 Summary of clinical, virologic, and serologic response to TV003 .... *27*
2.4.2.4 Summary of clinical, virologic, and serologic response to TV005 .... *28*
2.4.3 Mosquito Transmissibility of monovalent vaccine candidates 33
2.4.4 Participation of Children 34
3 OBJECTIVES **34**
3.1 Primary Objectives 34
3.2 Secondary Objectives 34
3.3 Exploratory Objectives 35
4 STUDY DESIGN **35**
4.1 Overall Design 35
4.2 Dosing Strategy 36
4.3 Sample Size and Placebo Ratio 36

4.4 Duration of Subject Participation 36
4.5 Estimated Duration of the Study 37
4.6 Treatment Assignment 37
4.7 Blinding 37

5 SELECTION AND ENROLLMENT OF SUBJECTS 38

5.1 Inclusion Criteria 38
5.2 Exclusion Criteria 38
5.3 Additional Inclusion Criteria for Second Dose of Vaccine 39
5.4 Exclusion Criteria for Second Dose of Vaccine 40
5.5 Other Treatments and Ongoing Exclusion Criteria 40
5.6 Subject Withdrawal and Termination Criteria 41
5.7 Access to Medical Records 42

6 VACCINE PREPARATION 42

6.1 Pre-Vaccination Preparation 42
6.2 Vaccine Storage 43
6.3 Vaccine Accountability 43
6.4 Storage Disposition of Used/Unused Supplies 43

7 STUDY PROCEDURES 43

7.1 Recruitment and General Screening 43
7.2 Screening Procedures 44
7.3 Immunization Procedure 45
7.4 Detailed Study Procedures 45
7.4.1 First Vaccination 45
7.4.2 Second Vaccination 47
7.5 Subject Temperature Memory Card 49
7.6 Clinical Laboratory Testing 50
7.7 Medical History and Concomitant Medications 50
7.8 Immunology Testing 51
7.8.1 Antibody Testing 51
7.8.2 Other Immunological Assays 51

8 ADVERSE EVENT MONITORING 51

8.1 Definitions 51
8.1.1 Adverse Event 51
8.1.2 Serious Adverse Event 52
8.1.3 Unexpected Adverse Events 53
8.1.4 Suspected and Unexpected Serious Adverse Reaction (SUSAR) 53
8.1.5 Unanticipated Problems 53
8.1.6 Pre-existing Conditions, Worsening of Pre-existing Condition 53
8.2 Assessment of Adverse Events 54
8.2.1 Identification of Adverse Events 54
8.2.2 Protocol Specific Adverse Event Definitions 54
8.2.3 Determination of Severity 54
8.2.4 Relationship with Receipt of Study Vaccine 57

8.2.5 Adverse Event Action Taken 57
8.2.6 Adverse Event Outcome 57
8.2.7 Adverse Event Seriousness 58
8.3 Adverse Event Reporting 58
8.3.1 Non-Serious Adverse Events 58
8.3.2 Serious Adverse Events 58
8.3.3 Unanticipated Problems 60
8.3.4 Pregnancy 60
8.4 Sponsor's Reporting Responsibilities 60
8.5 Stopping Criteria 60
8.6 Safety Oversight 61
8.6.1 Transfer of Regulatory Obligations (TORO) and Safety Review and Communications Plan (SRCP) 61
8.6.2 Data and Safety Monitoring Board (DSMB) 62
9 DATA COLLECTION AND MONITORING 62
9.1 Source Documentation and Data Collection 62
9.2 Study Documentation 63
9.3 Retention of Specimens 63
9.4 Retention of Records 63
9.5 Protocol Compliance 64
9.6 Clinical Investigator's Brochure 64
9.7 Study Monitoring 65
10 STATISTICAL CONSIDERATIONS 65
10.1 General Design 65
10.2 Statistical Methods 65
10.3 Safety 66
10.4 Immunogenicity 66
11 PROTECTION OF HUMAN SUBJECTS 67
11.1 Institutional Review Boards 67
11.2 Informed Consent 67
11.3 Risks 67
11.3.1 Venipuncture 67
11.3.2 Immunization 67
11.4 Benefits 68
11.5 Compensation 68
11.6 Confidentiality 68
11.7 Biohazard Containment 68
12 PUBLICATION POLICY 69
13 REFERENCES 70
APPENDIX 1: ADVERSE EVENT GRADING TABLE 74
14 SCHEDULE OF PROCEDURES POST EACH VACCINATION 80

List of Tables

TABLE 1: ABBREVIATIONS 8
TABLE 2: VACCINE COMPONENTS AND STUDY SUBJECTS 10
TABLE 3: CANDIDATE VACCINE VIRUSES ARE ATTENUATED IN RHESUS MACAQUES COMPARED WITH THEIR WILD-TYPE PARENT VIRUS 19
TABLE 4: PHASE 1 STUDIES OF MONOVALENT COMPONENTS OF TETRAVAX-DV 20
TABLE 5: CLINICAL RESPONSE TO MONOVALENT DENV VACCINE CANDIDATES FOR USE IN STUDY ADMIXTURES 24
TABLE 6: MAGNITUDE, ONSET, AND DURATION OF VIREMIA IN SUBJECTS INOCULATED WITH MONOVALENT DENV VACCINE CANDIDATES FOR USE IN TETRAVALENT VACCINE ADMIXTURES 25
TABLE 7: SERUM ANTIBODY RESPONSES INDUCED BY EACH MONOVALENT DENV CANDIDATE VACCINE GIVEN AS A SINGLE SUBCUTANEOUS DOSE 26
TABLE 8: CLINICAL RESPONSE TO 5 TETRAVALENT DEN VACCINE CANDIDATE ADMIXTURES 30
TABLE 9: MAGNITUDE, ONSET, AND DURATION OF VIREMIA IN SUBJECTS[1] INOCULATED WITH TV003 30
TABLE 10: MAGNITUDE, ONSET, AND DURATION OF VIREMIA IN SUBJECTS[1] INOCULATED WITH TV005 31
TABLE 11: SUMMARY OF SEROLOGIC RESPONSE INDUCED BY A SINGLE SUBCUTANEOUS DOSE OF TV003 OR TV005.. 31
TABLE 12: GEOMETRIC MEAN PEAK TITERS FOLLOWING VACCINATION WITH TV003 OR TV005 32
TABLE 13: PERCENT AND CUMULATIVE NEUTRALIZING ANTIBODY[1] RESPONSES FOLLOWING A SINGLE DOSE OF TV003 OR TV005.. 32
TABLE 14: SOLICITED ADVERSE EVENTS 52
TABLE 15: SEVERITY DEFINITIONS... 54
TABLE 16: ASSESSMENT OF SOLICITED ADVERSE EVENTS 56

List of Figures

FIGURE 1: LABEL FOR FINAL VIAL rDEN1Δ30 (ENLARGED SAMPLE)......................... 14
FIGURE 2: LABEL FOR FINAL VIAL RDEN2/4Δ30(ME) (ENLARGED SAMPLE) 15
FIGURE 3: LABEL FOR FINAL VIAL RDEN3Δ30/31-7164 LOT M10377008 (ENLARGED SAMPLE).. 17
FIGURE 4: LABEL FOR FINAL VIAL RDEN3Δ30/31-7164 LOT DEN3#113B (ENLARGED EXAMPLE) 17
FIGURE 5: LABEL FOR FINAL VIAL RDEN4Δ30 (ENLARGED SAMPLE) 18

Table 1: Abbreviations

AE	adverse event
ALT	alanine aminotransferase
ANC	absolute neutrophil count
AST	aspartate aminotransferase
C	capsid
CBC	complete blood count
CIR	Center for Immunization Research
CLIA	Clinical Laboratory Improvement Amendments
CPK	creatine phosphokinase
CRIMSON	Clinical Research Information Management System of the NIAID
CRL	Charles River Laboratories
CSO	Clinical Safety Office
DENV	dengue virus (serotypes DENV-1, DENV-2, DENV-3, and DENV-4)
DF	dengue fever
DHF	dengue hemorrhagic fever
DSMB	Data and Safety Monitoring Board
DSS	dengue shock syndrome
E	Envelope protein of dengue virus
FDA	Food and Drug Administration
GCP	Good Clinical Practice
HBsAg	hepatitis B surface antigen
HCG	human choriogonadotropin
HCV	hepatitis C virus
HID_{50}	50% human infectious dose
HIV	human immunodeficiency virus
HLA	human leukocyte antigen
ICH	International Conference on Harmonization
ICMJE	The International Committee of Medical Journal Editors
IND	investigational new drug
IRB	Institutional Review Board
JEV	Japanese encephalitis virus
JHSPH	Johns Hopkins Bloomberg School of Public Health
LID	Laboratory of Infectious Diseases
LLN	lower limit of normal
M	Membrane protein of dengue virus
MID_{50}	50% mosquito infectious dose
NIAID	National Institute of Allergy and Infectious Diseases

MM	Medical Monitor
NIH	National Institutes of Health
NS	non-structural
PBMC	peripheral blood mononuclear cell
PFU or FFU	plaque-forming units or focus-forming units (virus titer)
PI	principal investigator
PRNT	plaque reduction neutralization titer
PT/PTT	prothrombin time/ partial thromboplastin time
RCHSPB	Regulatory Compliance and Human Subjects Protection Branch
SAE	serious adverse event
SCID	severe combined immunodeficiency
SE	standard error
SLE	St. Louis encephalitis virus
TBEV	Tick-borne encephalitis virus
ULN	upper limit of normal
UTR	untranslated region
UVM	University of Vermont
UVM IRB	University of Vermont Institutional Review Board
WBC	white blood count
WHO	World Health Organization
WIRB	Western Institutional Review Board
WNV	West Nile virus
wt	wild type
YFV	Yellow fever virus

1 Protocol Summary

Protocol Title: A Phase 1 Evaluation of the Safety and Immunogenicity of the Recombinant Live Attenuated Tetravalent Dengue Virus Vaccine Admixtures TV003 and TV005 in Healthy Flavivirus-experienced Adult Subjects

Version: 4.0 FINAL

Version Date: **12 September 2013**

Revision History: v1.0 (07 Dec. 2011)
v2.0 (4 March 2012)
v3.0 (30 March 2012)

Phase: Phase 1

Subjects: Healthy male and non-pregnant female subjects 18 to 50 years of age with a documented history or serologic evidence of a previous flavivirus infection.

Number of Subjects: 112

Study Design: Placebo-controlled, double-blind study evaluating the safety and immunogenicity of the live attenuated tetravalent dengue vaccines TV003 and TV005 in healthy flavivirus-experienced adult subjects

Table 2: Vaccine Components and Study Subjects

Admixture	Total Subjects	Dose
TV003	40 vaccinees, 16 placebo recipients	10^3 PFU of rDEN1Δ30<br>10^3 PFU of rDEN2/4Δ30(ME)<br>10^3 PFU of rDEN3Δ30/31-7164<br>10^3 PFU of rDEN4Δ30
TV005	40 vaccinees, 16 placebo recipients	10^3 PFU of rDEN1Δ30<br>10^4 PFU of rDEN2/4Δ30(ME)<br>10^3 PFU of rDEN3Δ30/31-7164<br>10^3 PFU of rDEN4Δ30

Product Description: The vaccine candidate TetraVax-DV is a live attenuated recombinant tetravalent dengue virus vaccine. TV003 will contain 10^3 PFU of rDEN1Δ30, 10^3 PFU of rDEN2/4Δ30(ME), 10^3 PFU of rDEN3Δ30/31-7164, and 10^3 PFU of rDEN4Δ30. TV005 will contain 10^3 PFU of rDEN1Δ30, 10^4 PFU of rDEN2/4Δ30(ME), 10^3 PFU of rDEN3Δ30/31-7164, and 10^3 PFU of rDEN4Δ30.

2 Introduction

2.1 Background – Dengue

The World Health Organization (WHO) estimates that dengue viruses are responsible for more than 50 million cases of dengue fever (DF) and approximately one half million cases annually of the more severe disease, dengue hemorrhagic fever/shock syndrome (DHF/DSS) (21). Infection with dengue viruses (DENV) is the leading cause of hospitalization and death in children in at least 8 tropical Asian countries (33). There are 4 serotypes of dengue virus (DENV-1, DENV-2, DENV-3, and DENV-4), each capable of causing dengue illness ranging from a mild, self-limited febrile illness to life-threatening disease. Dengue viruses are endemic in most tropical and subtropical regions of the world with more than 2 billion persons at risk for acquiring dengue. For these reasons, the WHO has made development of a dengue vaccine a top priority (Resolution WHA 46.31).

Infection with one dengue serotype induces long-lived homotypic immunity and short-lived heterotypic immunity (24). Immunity is primarily mediated by neutralizing antibodies against the envelope (E) glycoprotein. Epidemiological studies have demonstrated that most cases of DHF/DSS occur in persons experiencing a second dengue infection with a serotype different from that which caused their first dengue infection (6). For this reason, DHF/DSS occurs predominately in children or adults living in dengue-endemic regions with multiple dengue serotypes circulating simultaneously. The goal of immunization is to induce a long-lived neutralizing antibody response against all 4 dengue serotypes. This can be best achieved economically using a live attenuated tetravalent virus vaccine delivered in one or more doses. Development of a live attenuated vaccine is a reasonable goal since it has already been achieved for the related yellow fever virus (YFV) and Japanese encephalitis virus (JEV), and other mosquito-borne flaviviruses present in tropical regions of the world (21, 27). Because a successful DENV vaccine will be deployed in dengue-endemic areas and will be administered to individuals that may have been previously exposed to dengue (or other flaviviruses), it will be important to evaluate the safety and immunogenicity of a LATV dengue vaccine in persons with a documented history of vaccination with a flavivirus or previous exposure to a DENV.

In humans, DENV infects predominately monocytes, dendritic cells, and lymphocytes and does not exhibit tropism for any particular organ, except perhaps the liver (35). The majority of primary and secondary infections with DENV are asymptomatic. Following an incubation period of approximately 1 week, self-limiting acute illness designated DF occurs that is characterized by a febrile period of about 5 days accompanied by systemic symptoms such as headache, malaise, anorexia, arthralgia, and myalgia. Viremia, rash (including petechial hemorrhages), lymphadenopathy, leucopenia, and thrombocytopenia can accompany the fever. Elevations of liver enzymes during DF are common, and the virus is known to infect human hepatocytes (7, 19, 20). The virus does not establish persistent infection and is usually eliminated by the end of the second week. DHF/DSS occurs much less commonly than DF. It develops at the time of defervescence and is characterized by an increased tendency to bleed into the skin or from mucous membranes and by a marked increase in vascular permeability resulting in hemoconcentration and

shock. This state of increased vascular permeability is short-lived (a few days) and with proper management is fatal in only about 1% of patients (21). Because previous infection with one DENV serotype may increase the risk for DHF/DSS following infection with a different serotype, it is clear that a DENV vaccine will need to protect against each of the 4 DENV serotypes, namely DENV-1, DENV-2, DENV-3, and DENV-4. However, it is unknown if a tetravalent dengue vaccine can be used safely in persons who have experienced at least one previous DENV or other flavivirus infection in the past.

The DENV genome contains a single open reading frame encoding a polyprotein which is processed by proteases of both viral and cellular origin into 3 structural proteins, namely the capsid (C), membrane, (M) and envelope (E) proteins, and at least 7 non-structural (NS) proteins. Each end of the DENV virus genome consists of an untranslated region (UTR), which is predicted to be highly structured. The E glycoprotein is on the surface of the virion, and immunity is mediated primarily by neutralizing antibodies to this protein. The E protein also defines each of the 4 DENV virus serotypes (DENV-1, DENV-2, DENV-3, and DENV-4). The 3′ untranslated region is highly conserved between the 4 dengue serotypes. Studies in mice, Rhesus monkeys, and mosquitoes were designed to evaluate the level of attenuation conferred independently by chimerization and the Δ30 (30 nucleotide deletion) mutation (32).

2.2 Background - Dengue Vaccines

Numerous studies of wild-type (wt) DENV and live attenuated dengue vaccine candidates have been conducted over the past 60 years. Various dengue live attenuated vaccine candidate viruses have been administered to nearly 1500 subjects in Phase 1 and Phase 2 clinical studies (1, 2, 14, 15, 18, 23, 25, 26, 28, 34). The majority of dengue vaccines tested to date have been biologically derived by repeated passage in tissue culture. Although some of these vaccine candidates have appeared promising in early clinical development, none has achieved the ideal balance between reactogenicity and immunogenicity, particularly when included as part of a tetravalent vaccine formulation (2, 23). It has been difficult to achieve adequate antibody responses to all 4 serotypes when the vaccine viruses are administered in the tetravalent formulation. Common adverse events (AEs) noted in these studies were signs and symptoms of mild DF, notably fever, headache, myalgias, rash, neutropenia, and elevated liver function tests. In the published literature, all subjects who experienced any AE recovered completely and without sequelae. Of note, no live attenuated dengue vaccine candidate to date has induced severe dengue disease. The lack of a suitable licensed tetravalent live attenuated vaccine candidate forms the basis for testing the TetraVax-DV candidate described in this protocol.

2.3 Vaccine Descriptions

The TetraVax-DV TV003 and TV005 admixtures will consist of 4 different dengue vaccine candidates, representing each of the 4 serotypes: rDEN1Δ30, rDEN2/4Δ30(ME), rDEN3Δ30/31-7164, and rDEN4Δ30 (See **Table 2**). TV003 and TV005 differ in the dose of the DENV-2 component, rDEN2/4Δ30(ME). The dose of this component is 10-fold higher in TV005 (see **Table 2**) than in TV003. It has been determined that the monovalent vaccine candidates may be stored at -80 ± 15°C. This recommended temperature range, although not reflected on the monovalent vaccine labels, will be instituted for the storage of the vaccine candidates.

2.3.1 rDEN1Δ30 Vaccine

The vaccine candidate rDEN1Δ30 is a live attenuated virus derived from the DENV-1 Western Pacific (WP) wt strain of dengue using recombinant DNA technology. A full-length cDNA clone of the DENV-1 WP genome was constructed and a 30-nucleotide deletion was then created in the 3′ untranslated region. Genome-length, capped, RNA transcripts were synthesized, purified, and transfected into qualified Vero cells. Recovered virus was terminally diluted and then amplified by serial passaging in Vero cells. The final amplification of virus was made in serum-free medium and the titer was determined by plaque titration in Vero cells. From initial transfection through final amplification, only serum-free medium was used for Vero cell culture and propagation of virus. Reagents used during the transfection process and present initially in fluid harvested from the transfection were diluted more than 10^{12}-fold as a result of the biological cloning (terminal dilution) and amplification of the virus. The rDEN1Δ30-1545 seed virus was generated in the Laboratory of Infectious Diseases (LID), NIAID, and the vaccine was manufactured at Charles River Laboratories (CRL) Biopharmaceutical Services facility in Malvern, PA. The Final Drug Product, rDEN1Δ30-1545 (Lot DEN1#104A) was manufactured in qualified Vero cells at CRL on 26 August 2004 based on a method developed by LID/NIAID/NIH and CRL.

2.3.1.1 Final Container rDEN1Δ30

The Final Drug Product was dispensed as 0.6 mL aliquots of approximately $10^{6.8}$ plaque-forming units (PFU)/mL Live Recombinant Dengue Virus Type 1 rDEN1Δ30-1545 Vero Grown Virus Vaccine into 2.0 mL sterile cryovials.

2.3.1.2 Composition rDEN1Δ30

The Final Drug Product composition is a concentration of live attenuated rDEN1Δ30-1545 (Lot DEN1#104A) in Leibovitz L-15 Medium containing 1X SPG (sucrose, 0.218 M; KH_2PO_4, 0.0038 M; K_2HPO_4, 0.0072 M; mono-sodium glutamate, 0.0054 M). The potency of rDEN1Δ30-1545 (Lot DEN1#104A) is $10^{6.8}$ PFU/mL.

FIGURE 1: LABEL FOR FINAL VIAL rDEN1Δ30 (ENLARGED SAMPLE)

<table>
<tr><td rowspan="3">0804- Vial #
Lot DEN1#104A</td><td>Live Recombinant Dengue Virus Type 1
rDEN1Δ30-1545
Vero Grown Virus Vaccine</td></tr>
<tr><td>CAUTION: NEW DRUG LIMITED
BY FEDERAL (USA) LAW
TO INVESTIGATIONAL USE</td></tr>
<tr><td>Store at -70°C or below
Charles River Laboratories
Malvern PA</td></tr>
</table>

*NOTE: Since manufacture of the Final Drug Product, evaluation of live attenuated dengue virus vaccines (developed at the LID/NIH/ NIAID) in both laboratory and clinical situations indicates that these viruses are stable and potent when stored at a temperature of -80°C ± 15°C. Therefore, -80°C ± 15°C is the recommended storage condition for the Final Drug Product.

2.3.2 rDEN2/4Δ30(ME) Vaccine

The rDEN2/4Δ30(ME) virus is a live attenuated chimeric virus derived from rDEN4Δ30. A full-length cDNA copy of rDEN4Δ30 was constructed in which the M and E coding sequences of DENV-4 were replaced with those of DENV-2 NGC prototype. Genome-length, capped, RNA transcripts were synthesized, purified, and transfected into qualified Vero cells. Recovered virus was terminally diluted and then amplified by serial passaging in Vero cells. The final amplification of virus was made in serum-free medium and the titer was determined by plaque titration in Vero cells. The rDEN2/4Δ30(ME)-1495,7163 seed virus was generated in LID/NIAID and then manufactured at Charles River Laboratories (CRL) Biopharmaceutical Services facility in Malvern, PA. The Final Drug Product, rDEN2/4Δ30(ME)-1495,7163 (Lot DEN2/4#106C) was manufactured in qualified Vero cells at CRL on 8 February 2007 based on a method developed by LID/NIAID/NIH and CRL.

2.3.2.1 Final Container rDEN2/4Δ30(ME)

The Final Drug Product was dispensed as 0.6 mL aliquots of approximately $10^{7.8}$ PFU/mL Live Recombinant Dengue Virus Type 2 rDEN2/4Δ30(ME)-1495,7163 Vero Grown Virus Vaccine into 2.0 mL sterile cryovials (Corning #430659).

2.3.2.2 Composition rDEN2/4Δ30(ME)

The Final Drug Product composition is a concentration of live attenuated rDEN2/4Δ30(ME)-1495,7163 (Lot DEN2/4#106C) in Leibovitz L-15 Medium containing 1X SPG (sucrose, 0.218 M; KH_2PO_4, 0.0038 M; K_2HPO_4, 0.0072 M; mono-sodium glutamate, 0.0054 M). The potency of rDEN2/4Δ30(ME)-1495,7163 (Lot DEN2/4#106C) is $10^{7.8}$ PFU/mL.

FIGURE 2: LABEL FOR FINAL VIAL rDEN2/4Δ30(ME) (ENLARGED SAMPLE)

**Live Recombinant Dengue Virus Type 2**<br>**rDEN2/4Δ30(ME)-1495,7163**<br>**VERO Grown Virus Vaccine**	**Lot DEN 2/4 #106C**<br>**0207-vial #**
**CAUTION: NEW DRUG LIMITED**<br>**BY FEDERAL (USA) LAW**<br>**TO INVESTIGATIONAL USE**	
Store at -70±10°C<br>Charles River Laboratories, Malvern, PA	

*NOTE: Since manufacture of the Final Drug Product, evaluation of live attenuated dengue virus vaccines (developed at the LID/NIH/ NIAID) in both laboratory and clinical situations indicates that these viruses are stable and potent when stored at a temperature of -80°C ± 15°C. Therefore, -80°C ± 15°C is the recommended storage condition for the Final Drug Product.

2.3.3 rDEN3Δ30/31-7164

rDEN3Δ30/31-7164 was constructed by further mutating the DEN3 3´ UTR of the DENV-3 Sleman/78 by combining the Δ30 deletion with an additional, 31 nucleotide deletion in the DEN3 3´UTR (5). Since the flavivirus 3´ UTR is non-coding, it is well suited for use as a genetic element to condition attenuation in DENV vaccine candidates. The attenuation phenotype of the rDEN3Δ30/31-7164 virus vaccine is expected to be phenotypically stable since the Δ30 mutation was shown to be stable following administration of the DEN4Δ30 virus vaccine to human subjects (8). Genome-length, capped, RNA transcripts were synthesized from the linearized p3Δ30/31-7164 clone #28 using the AmpliCap SP6 Message Maker Kit (EpiCentre Technologies, Madison, WI; Lot AC650408) and purified using the RNeasy Mini Kit (Qiagen, Valencia, CA). Virus was recovered in qualified C6/36 cells (Sponsor's Master File, BB-MF #11371) transfected on 6 January 2006 with purified RNA transcripts using DOTAP liposomal transfection reagent (Roche, Indianapolis, IN). The rDEN3Δ30/31-7164 Seed Virus was transferred from LID/NIAID/NIH to Meridian Life Sciences, Inc. (Memphis, TN) for production of the virus vaccine. The virus was amplified by 1 passage on Vero cells in serum free medium to produce the Final Drug Product, rDEN3Δ30/31-7164 Lot M10377008. The Final Drug Product was manufactured on 25 January 2008.

An aliquot of the seed virus was later submitted to Charles River Laboratories (CRL), Biopharmaceutical Services (Malvern, PA), for production of a second lot of vaccine. The lot was amplified by 1 passage on Vero Cells in serum free medium to produce the Final Drug Product, rDEN3Δ30/31-7164 Lot DEN3#113B. Final drug product was manufactured on 18 January 2012.

2.3.3.1 Final Container of rDEN3Δ30/31-7164 Lot M10377008

The Final Drug Product was dispensed as 0.6 mL aliquots of approximately $10^{7.3}$ PFU/mL of Live Recombinant Dengue Virus Type 3 rDEN3Δ30/31-7164 Vero Grown Virus Vaccine into 2.0 mL sterile cryovials.

2.3.3.2 Composition of rDEN3Δ30/31-7164 Lot M10377008

The Final Drug Product composition is a concentration of live attenuated rDEN3Δ30/31-7164 in L-15 medium containing 1X SPG (sucrose, 0.218 M; KH_2PO_4, 0.0038 M; K_2HPO_4, 0.0072 M; mono-sodium glutamate, 0.0054 M). The titer of rDEN3Δ30/31-7164, Lot M10377008, is $10^{7.3}$ PFU/mL.

Figure 3: Label for Final Vial rDEN3Δ30/31-7164 Lot M10377008 (Enlarged Sample)

Live Recombinant
Dengue Virus Type 3
rDEN3Δ30/31-7164
VERO Grown Virus Vaccine

CAUTION: NEW DRUG LIMITED
BY FEDERAL (USA) LAW
TO INVESTIGATIONAL USE

Lot M10377008
Part: 80118
Date: 01/25/08
Vial # XXXX
Store at -70±10°C
Meridian Life Science
5175 Wilfong Road
Memphis, TN 38134
USA

*NOTE: Since manufacture of the Final Drug Product, evaluation of live attenuated dengue virus vaccines (developed at the LID/NIH/ NIAID) in both laboratory and clinical situations indicates that these viruses are stable and potent when stored at a temperature of -80°C ± 15°C. Therefore, -80°C ± 15°C is the recommended storage condition for the Final Drug Product.

2.3.3.3 Final Container of rDEN3Δ30/31-7164 Lot DEN3#113B

The Final Drug Product was dispensed as 0.6 mL aliquots of approximately $10^{7.7}$ PFU/mL of Live Recombinant Dengue Virus Type 3 rDEN3Δ30/31-7164 Vero Grown Virus Vaccine into 2.0 mL sterile cryovials.

2.3.3.4 Composition of rDEN3Δ30/31-7164 Lot DEN3#113B

The Final Drug Product composition is a concentration of live attenuated rDEN3Δ30/31-7164 in L-15 medium containing 1X SPG (sucrose, 0.218 M; KH_2PO_4, 0.0038 M; K_2HPO_4, 0.0072 M; mono-sodium glutamate, 0.0054 M). The titer of rDEN3Δ30/31-7164 Lot DEN3#113B is $10^{7.7}$ PFU/mL.

Figure 4: Label for Final Vial rDEN3Δ30/31-7164 Lot DEN3#113B (Enlarged Example)

Live Recombinant Dengue Virus Type 3
rDEN3Δ30/31-7164
VERO Grown Virus
CAUTION: NEW DRUG LIMITED
BY FEDERAL (USA) LAW
TO INVESTIGATIONAL USE
Store at -70±10°C
Charles River Laboratories, Malvern, PA

Date: 18JAN2012
Vial#: XXXX
Lot: DEN3#113B

*NOTE: Evaluation of live attenuated dengue virus vaccines (developed at the LID/NIH/ NIAID) in both laboratory and clinical situations indicates that these viruses are stable and potent when stored at a temperature of -80°C ± 15°C. Therefore, -80°C ± 15°C is the recommended long-term storage condition for the Final Drug Product.

2.3.4 rDEN4Δ30 Vaccine Lot# 109A

The vaccine candidate rDEN4Δ30 is a live attenuated virus derived from the DENV-4 Dominica/81 wt strain of DENV using recombinant DNA technology. A full-length cDNA clone of the DENV-4 Dominica/81 genome was constructed and a 30-nucleotide deletion was then created in the 3′ untranslated region. Genome-length, capped, RNA transcripts were synthesized, purified, and transfected into qualified Vero cells. Recovered virus was terminally diluted and then amplified by serial passaging in Vero cells. The final amplification of virus was made in serum-free medium and the titer was determined by plaque titration in Vero cells. From initial transfection through final amplification, only serum-free medium was used for Vero cell culture and propagation of virus. The rDEN4Δ30-7132,7163, 8308 seed virus was generated at LID/NIAID, and vaccine was manufactured at Charles River Laboratories (CRL) Biopharmaceutical Services facility in Malvern, PA. The Final Drug Product, rDEN4Δ30-7132, 7163, 8308 (Lot DEN4#109A) was manufactured in qualified Vero cells at CRL on 18 July 2007 based on a method developed by LID/NIAID/NIH and CRL.

2.3.4.1 Final Container rDEN4Δ30 Lot# 109A

The Final Drug Product was dispensed as 0.6 mL aliquots of approximately $10^{7.2}$ PFU/mL Live Recombinant Dengue Virus Type 4 rDEN4Δ30-7132,7163,8308 Vero Grown Virus Vaccine into 2.0 mL sterile cryovials (Corning #430659).

2.3.4.2 Composition rDEN4Δ30 Lot#109A

The Final Drug Product composition is a concentration of live, attenuated rDEN4Δ30-7132, 7163, 8308 (Lot DEN4#109A) in L-15 medium containing 1X SPG. The potency of rDEN4Δ30-7132, 7163, 8308 (Lot DEN4#109A) is $10^{7.2}$ PFU/mL.

Figure 5: Label for Final Vial rDEN4Δ30 (Enlarged Sample)

<table>
<tr><td>Live Recombinant Dengue Virus Type 4
rDEN4Δ30-7132,7163,8308
VERO Grown Virus Vaccine</td><td rowspan="3">Lot DEN 4 #109A
0707-vial #</td></tr>
<tr><td>CAUTION: NEW DRUG LIMITED
BY FEDERAL (USA) LAW
TO INVESTIGATIONAL USE</td></tr>
<tr><td>Store at -70±10°C
Charles River Laboratories, Malvern, PA</td></tr>
</table>

*NOTE: Since manufacture of the Final Drug Product, evaluation of live attenuated dengue virus vaccines (developed at the LID/NIH/ NIAID) in both laboratory and clinical situations indicates that these viruses are stable and potent when stored at a temperature of -80°C ± 15°C. Therefore, -80°C ± 15°C is the recommended storage condition for the Final Drug Product.

2.4 Rationale

2.4.1 Animal Experience

Each of these candidate vaccine viruses has been evaluated in juvenile Rhesus monkeys and in the novel rodent model consisting of severe combined immunodeficiency (SCID) mice bearing intraperitoneal tumors of the human liver cell line HuH-7. Replication of these candidate vaccine viruses in the SCID-HuH-7 mouse model was compared with that of their wt parent viruses. The candidate vaccine viruses were attenuated in the SCID-HuH-7 mice, replicating to a peak titer from $10^{0.9}$ (rDEN4Δ30) up to $10^{2.4}$ (rDEN4Δ30-200,201) lower than that of their wt parent DENV (5, 16, 31, 32). Juvenile Rhesus monkeys were inoculated subcutaneously with monovalent doses (10^5 PFU) of rDEN1Δ30, rDEN2/4Δ30(ME), rDEN3-3′D4Δ30, rDEN3Δ30/31-7164, rDEN4Δ30, or the wt parent DENV (5, 16, 31, 32). All monkeys inoculated with wt virus became viremic with the mean duration of viremia ranging from 2.8 days (DENV-1) to 5.5 days (DENV-2) (**Table** 3). Fewer monkeys inoculated with the candidate vaccine viruses were viremic compared with the parent virus for each vaccine candidate except rDEN4Δ30. In addition, the mean number of days of viremia and the mean peak titer were also reduced for each candidate vaccine virus compared with its parent virus (**Table** 3).

Table 3: Candidate vaccine viruses are attenuated in Rhesus macaques compared with their wild-type parent virus

Virus	Dose (log_{10} PFU)	No. of monkeys	No. Viremic	Mean # of days viremic	Mean peak serum titer (log_{10}PFU/ mL)
DENV-1 (wt)	5	4	4	2.8	2.1 ± 0.1
**rDEN1Δ30**	5	4	2	0.5	0.8 ± 0.1
DENV-2 (wt)	5	4	4	5.5	2.1 ± 0.15
**rDEN2/4Δ30(ME)**	5	4	1	0.3	0.7 ± 0.02
DENV-3 (wt)	5	4	4	3.5	1.8 ± 0.1
**rDEN3Δ30/31-7164**	5	4	0	n/a[1]	n/a[1]
DENV-4 (wt)	5	4	4	3	2.7 ± 0.09
**rDEN4Δ30**	5	6	6	2.5	2.0 ± 0.15

1. Not applicable

A tetravalent admixture of the live attenuated dengue vaccine candidates was evaluated for safety/toxicity, virus replication and immunogenicity in Rhesus macaques. The admixture included rDEN1Δ30, rDEN2/4Δ30(ME), rDEN3-3′D4Δ30, and rDEN4Δ30 (designated 'TV1'). The animals received 10^5 PFU of each of the component viruses in the admixture in a single subcutaneous injection of 0.5 mL. Monkeys were observed twice daily for mortality and morbidity, as well as for signs of neurologic, hemorrhagic or dermal disease. No signs consistent with these diseases were observed in any of the animals. Multiple episodes of lymphopenia were seen in all monkeys inoculated with TV1, and 3 of the 4 monkeys inoculated with placebo. These results were seen throughout the study including the samples taken on Day -8 and Day 0 prior to

inoculation. Mild deviations from the normal reference range were noted on the clinical pathology profiles for most monkeys but are not likely to be of statistical or biological significance.

The DENV-4 component of the tetravalent vaccine was the only virus detected in any of the monkeys inoculated with the TV1 admixture. The rDEN4Δ30 vaccine virus was detected at low titer ($10^{1.0}$ to $10^{1.3}$ PFU/mL) in 3 of the 4 monkeys that received TV1, and only on Study Day 2. These findings confirm the attenuation of each of the component vaccine viruses.

Despite the low/undetectable levels of viremia observed in the animals, the tetravalent vaccine admixture elicited neutralizing antibody titers against the component vaccine viruses. Inoculation of monkeys with TV1 resulted in 75-100% seroconversion to DENV-1, DENV-2, DENV-3, and DENV-4.

In summary, the TV1 tetravalent dengue virus vaccine formulation was safe and well-tolerated in Rhesus monkeys, and elicited antibody responses against the component viruses.

2.4.2 Clinical Experience with the recombinant live attenuated dengue vaccine candidates

Each of the candidate live attenuated DENV vaccines included in TetraVax-DV has been evaluated in at least 1 Phase 1 clinical trial (See **Table 4**). These trials were conducted at the Center for Immunization Research (CIR) at the Johns Hopkins Bloomberg School of Public Health (JHSPH) and the University of Vermont. The experience with each of these vaccine candidates is summarized below. The absence of clinically apparent dengue-like illness is likely a result of the high degree of attenuation of the vaccine viruses. Peak viremia titers observed in the studies described below were more than 1,000-fold lower than those observed with symptomatic wt DENV infection (17, 22, 30). In preparation of performing studies of a LATV vaccine in persons with existing flavivirus antibody, a subjects who had received a live attenuated monovalent DENV vaccine were given a second, heterotypic monovalent vaccine approximately 6 months to 7 years following the initial vaccination (11). There was no increase in vaccine reactogenicity in subjects who received a heterotypic vaccine. In only one cohort was an increase in mean peak titer observed. This was observed in the cohort that received rDEN2/4Δ30 4 to 7 years following rDEN4Δ30. The increase in mean peak titer was small (0.5 log_{10} PFU/mL in primary vaccination vs 1.2 log_{10} PFU/mL following second, heterotypic vaccination).

Table 4: Phase 1 studies of monovalent components of TetraVax-DV

Serotype	Vaccine candidate	Lot number	IND number
DENV-1	rDEN1Δ30	DEN1#104A	BB-IND-11677
DENV-2	rDEN2/4Δ30(ME)	DEN2/4#106C	BB-IND-11938
DENV-3	rDEN3Δ30/31-7164	M10377008	BB-IND-13886
DENV-4	rDEN4Δ30	DEN4#109A	BB-IND-8463

2.4.2.1 Clinical experience with DENV-1, DENV-2, DENV-3, and DENV-4 monovalent vaccine candidates

rDEN1Δ30 was first evaluated at a dose of 10^3 PFU in 2 placebo-controlled Phase 1 studies at the CIR/JHSPH (3). In a single-dose-study, 28 healthy male and non-pregnant female adult subjects, between the ages of 18 and 50, were enrolled and were randomly assigned to receive vaccine or placebo; 20 subjects received vaccine, 8 subjects received placebo (vaccine diluent). In a two-dose schedule comparison study of rDEN1Δ30 lotDEN1#104A, 2 cohorts of 30 healthy male and non-pregnant female adult subjects between the ages of 18 and 50 were randomly assigned to receive their second dose of vaccine either 120 days or 180 days post-Dose 1 (13). In each cohort, 25 subjects were randomly assigned to receive vaccine and 5 subjects were randomly assigned to receive placebo. One subject in this study was replaced after receiving vaccine and a second subject was replaced after receiving placebo. Thus far, a total of 71 subjects have received rDEN1Δ30 at a dose of 10^3 PFU. The vaccine was found to be safe and immunogenic (**Table 5, Table 6, and Table 7).** The candidate vaccine was further studied at a dose of 10^1 PFU to determine the 50% human infectious dose (HID_{50}) of this candidate vaccine. Fourteen out of 15 vaccinees (93%) were infected with 10^1 PFU of vaccine, indicating that the HID_{50} is well below 10^1 PFU.

A total of 40 adult flavivirus-naïve subjects have received the rDEN2/4Δ30(ME) candidate DENV-2 vaccine at a dose of 10^3 PFU. rDEN2/4Δ30(ME) was first evaluated at a dose of 10^3 PFU in a Phase 1, placebo-controlled trial conducted at the CIR (10). Twenty-eight healthy male and non-pregnant female adult subjects, between the ages of 18 and 50, were enrolled; 20 subjects received vaccine, 8 subjects received placebo (vaccine diluent). The vaccine was found to be safe and immunogenic (**Table 5, Table 6, and Table 7).** rDEN2/4Δ30(ME) was also evaluated in a 2-dose study at a dose level of 10^3 PFU, with the second dose given at 4 or 6 months post Dose 1. A total of 50 subjects were enrolled (25 in each cohort). Twenty subjects received 10^3 PFU of rDEN2/4Δ30(ME) subcutaneously at Day 0 and again at 6 months. Five subjects were enrolled as placebo-controls and received vaccine diluent at time 0 and 4 months. Because no subject was infected with the second dose given at 6 months, subjects were not enrolled in the cohort evaluating a second dose given at 4 months. The vaccine was found to be safe, well tolerated, and immunogenic (**Table 5, Table 6, and Table 7**).

Eighteen adult flavivirus-naïve subjects were enrolled in a trial evaluating the safety and immunogenicity of rDEN2/4Δ30(ME) given as a single subcutaneous dose of 10^1 PFU. Fifteen subjects received vaccine; 3 subjects received placebo. A dose of 10^1 PFU was given in order to determine the HID_{50} of the candidate vaccine. Fifty-three percent of vaccinees were infected by a dose of 10^1 PFU indicating that the HID_{50} of this candidate is approximately 10^1 PFU. Because of the lower infectivity of this individual candidate vaccine and the results of a study evaluating the safety and immunogenicity of rDEN2/4Δ30(ME) given at 10^3 PFU in a tetravalent admixture (see below) it is planned to evaluate a 10-fold higher dose of rDEN2/4Δ30(ME) [10^4 PFU] in a tetravalent admixture, TV005, in this protocol.

rDEN3Δ30/31-7164 has been evaluated at a dose of 10^3 PFU given as a single subcutaneous dose in a Phase 1, placebo-controlled trial conducted at the CIR and the

University of Vermont. Seventy male and non-pregnant female adult subjects, between the ages of 18 and 50, were enrolled; 50 subjects received vaccine, 20 received placebo (vaccine diluent). The vaccine was found to be safe and immunogenic (**Table 5, Table 6, and Table 7**). The candidate vaccine was further studied at a dose of 10^1 PFU to determine the HID_{50} of this candidate vaccine. Eighteen out of 20 vaccinees (90%) were infected with 10^1 PFU of vaccine, indicating that the HID_{50} is well below 10^1 PFU.

rDEN4Δ30, Lot 4-9 was evaluated in 2 clinical trials (8, 12). In the first study, 20 healthy male and non-pregnant female adult subjects received 10^5 PFU of the candidate vaccine as a single subcutaneous dose. In the second study, 20 healthy male and non-pregnant female adult subjects received this vaccine at a dose of 10^3 PFU, 10^2 PFU, or 10^1 PFU. Twelve subjects received vaccine diluent as a placebo. The vaccine was found to be safe and immunogenic (**Table 5, Table 6, and Table 7**). The HID_{50} was determined to be less than 10 PFU. Five subjects who received 10^5 PFU of the rDEN4Δ30 candidate vaccine developed a transient elevation in serum alanine aminotransferase (ALT) levels. Elevation in serum ALT was abrogated by decreasing the dose of the candidate vaccine (**Table 5).** Thus far, a total of 80 subjects have received rDEN4Δ30.

Because the clinical supply of Lot# 4-9 of rDEN4Δ30 was exhausted, a second clinical lot of rDEN4Δ30, Lot# 109A, was manufactured. Seventy healthy adult male and non-pregnant female subjects were enrolled and received a single subcutaneous 10^3 dose of rDEN4Δ30 (50 subjects) or placebo (20 subjects). The vaccine was well tolerated by all subjects. The most common vaccine related AE reported was an asymptomatic, transient maculopapular rash in 20 of 50 (40%) vaccinees (**Table 5**). One vaccinee developed an elevated serum ALT level. Lot# 109A will be used as the DENV-4 component for these tetravalent vaccine studies.

2.4.2.2 Summary of clinical, virologic, and serologic response to monovalent candidates

All of the vaccines described above have been well tolerated and were found to be both safe and strongly immunogenic when administered as monovalent vaccines. The safety, infectivity, and immunogenicity profiles of the monovalent vaccines were found to be comparable. (**Table 5, Table 6, and Table 7**) (3). Vaccinees did not develop a dengue-like illness at any dose (dengue-like illness is defined in **Section 8.5**). Local reactogenicity was minimal in all subjects. The most common AEs observed in all studies were transient neutropenia, rash, and headache (**Table 5**). Fifty-three of 231 vaccinees (23%) developed a transient neutropenia following vaccination. The neutropenia was graded as mild (1,000 – 1500/mm^3) in 48 vaccinees (16%), as moderate (750 – 1499/mm^3) in 9 vaccinees (3%) and as severe (<750/mm^3) in 7 vaccinees (2%). The neutropenia was of short duration (generally 3 – 5 days). All episodes of severe neutropenia were ≤4 days. None of the neutropenic subjects developed clinical complications. A total of 92 vaccinees (40%) developed a transient maculopapular rash over the trunk and proximal upper extremities following first vaccination. The rash was characteristically non-pruritic and went unnoticed by the majority of affected subjects. A total of 92 vaccinees (40%) complained of headache; however, this was comparable to the number of headaches reported in placebos (36%). None of the 231 subjects developed

a fever. Other AEs experienced in the 28 day follow-up period following vaccination included arthralgia, myalgia, malaise, retro-orbital pain, and injection site reactions. These occurred in fewer than 10% of vaccinated subjects.

Vaccine virus was recovered from the blood of subjects inoculated with each monovalent DENV vaccine candidate **(Table 6)**. The mean peak titers of the monovalent DEN candidate vaccine viruses were very low, ranging from $10^{0.5}$ PFU/mL (recipients of 10^3 PFU of rDEN3Δ30/31-7164 and rDEN4Δ30) to $10^{1.6}$ PFU/mL (recipients of 10^5 PFU of rDEN4Δ30) (**Table 6**).

Table 5: Clinical response to monovalent DENV vaccine candidates for use in study admixtures

Vaccine candidate	Dose (log_{10}PFU)	No. of subjects	% viremic	No. of subjects (%) with indicated clinical response				
				Fever	Rash	Headache	Neutropenia[2]	↑ALT
**rDEN1Δ30**	**3**	**71**	**60**	**1[1] (1.4)**	**22 (31)**	**29 (41)**	**32 (45)**	**1 (1.4)**
**rDEN2/4Δ30(ME)**	**3**	**40**	**60**	**0 (0)**	**13 (32)**	**12 (30)**	**11 (28)**	**3 (8)[2]**
**rDEN3Δ30/31-7164**	**3**	**50**	**34**	**0 (0)**	**26 (52)**	**18 (36)**	**2 (4)**	**1 (2)[1]**
**rDEN4Δ30 (Lot 4-9)**	**3**	**20**	**35**	**0 (0)**	**11 (55)**	**7 (35)**	**5 (25)**	**1 (5)**
**rDEN4Δ30 (Lot 109A)**	**3**	**50**	**26**	**0 (0)**	**20 (40)**	**26 (52)**	**3 (6)**	**1 (2)**
Placebo[3]	n/a[4]	76	**0**	1 (1)	2 (3)	27 (36)	6 (8)	3 (4)

1. Not related to vaccine.
2. Peak ALT levels in these subjects ranged from 1.3 to 1.7 x ULN. 2/3 subjects with elevated ALT had an ALT above the ULN on Day 0 prior to vaccination despite being normal at screening.
3. Includes the placebo recipients enrolled in the clinical trial of the monovalent candidates listed in this table.
4. Not applicable.

Table 6: Magnitude, onset, and duration of viremia in subjects inoculated with monovalent DENV vaccine candidates for use in tetravalent vaccine admixtures

Vaccine candidate	Dose (log_{10}PFU)	N	(%) with viremia	Mean peak titer ± SE (log_{10} PFU/mL)[1]	Mean day of onset of viremia ± SE	Mean # of days of viremia ± SE
**rDEN1Δ30**	**3**	**71**	**60**	**1.0 ± 0.08**	**10.0 ± 0.3**	**3.3 ± 0.3**
**rDEN2/4Δ30(ME)**	**3**	**40**	**60**	**0.5 ± 0.03**	**9.2 ± 0.6**	**3.3 ± 0.6**
**rDEN3Δ30/31-7164**	**3**	**50**	**34**	**0.6 ± 0.01**	**7.6 ± 0.64**	**3.2 ± 0.5**
**rDEN4Δ30 (Lot 4-9)**	**3**	**20**	**35**	**0.5 ± 0.1**	**9.1 ± 1.2**	**1.6 ± 0.8**
**rDEN4Δ30 (Lot 109A)**	**3**	**50**	**26**	**0.7 ± 0.1**	**10.8 ± 0.7**	**2.2 ± 0.4**

1. Mean peak titer is calculated only for those subjects who were viremic. Lower limit of detection is 0.5 log_{10} PFU/mL.

Table 7: Serum antibody responses induced by each monovalent DENV candidate vaccine given as a single subcutaneous dose

Vaccine candidate	Dose ($\log_{10}$)	No. vaccinees	%. infected[2]	Geometric mean titer, reciprocal (range)[1]			% seroconverted[3]
				Day 0	Day 28	Day 42	
**rDEN1Δ30**	**3**	**70**	**94**	**<5**	**170 (<5 - 6309)**	**140 (<5 – 2753)**	**93[4]**
**rDEN2/4Δ30(ME)**	**3**	**40**	**100**	**<10**	**88 (11– 1043)**	**104 (11 - 1377)**	**100**
**rDEN3Δ30/31-7164**	**3**	**50**	**82**	**<5**	**84 (14-1715)**	**79 (8-1341)**	**81**
**rDEN4Δ30 (Lot 4-9)**	**3**	**20**	**95**	**<10**	**139 (<10 – 2365)**	**129 (15 – 1222)**	**95**
**rDEN4Δ30 (Lot 109A)**	**3**	**50**	**93**	**<5**	**112 (12 – 892)**	**135 (22 - 748)**	**93**

1. Geometric mean titer calculated only for those subjects who seroconverted.
2. Defined as either recovery of vaccine virus from the blood or by seroconversion.
3. Defined as a ≥4-fold rise in serum neutralizing titer compared to Day 0.

Each of the monovalent candidate vaccines induced seroconversion (as defined as a ≥4-fold rise in serum neutralizing antibody titer at Study Day 28 or 42 compared with Study Day 0) in a large majority of vaccinees (**Table 7**). Seroconversion rates ranged from 81% (rDEN3Δ30/31-7164) to 100% (rDEN2/4Δ30(ME), rDEN4Δ30). rDEN1Δ30 induced sterilizing immunity to a second dose of vaccine administered at 4 or 6 months, in all vaccinees (data not shown).

2.4.2.3 Summary of clinical, virologic, and serologic response to TV003

Five different tetravalent admixtures have been evaluated in healthy adult volunteers, including TV003. Two different DENV-3 candidate vaccines (rDEN3Δ30/31-7164 and rDEN3-3′D4Δ30) and 2 different DENV-4 candidate vaccines (rDEN4Δ30 and rDEN4Δ30-200,201) were evaluated in combination with rDEN1Δ30 and rDEN2/4Δ30(ME). All viruses were given subcutaneously. DENV-1, DENV-3, and DENV-4 were given at a dose of 10^3 PFU in all tested admixtures. DENV-2 was given at a dose of 10^3 PFU in admixtures in TV001, TV002, TV003 and TV004 and at a dose of 10^4 PFU in TV005. For each admixture, 20 subjects received vaccine and 8 subjects received a placebo (vaccine diluent). For comparison, the safety data for all five tetravalent admixtures is presented in **Table 8**. The most common AE related to vaccine across all cohorts was an asymptomatic rash similar to that reported for the monovalent vaccines and headache. All of the tetravalent admixtures were well tolerated (**Table 8**). TV003 (bolded in table) was well tolerated by the subjects. There was 1 serious adverse event (SAE), an episode of cholecystitis at Study Day 156. The subject underwent cholecystectomy. The cholecystitis was unrelated to vaccine. No subject developed fever following vaccination. Fifteen vaccinees developed a dengue vaccine virus-like rash. The day of onset of the rash ranged from Study Day 9 to Study Day 15. The rash was of mild severity in all subjects. Three vaccinees and 1 placebo recipient developed a mild neutropenia. One vaccinee developed moderate neutropenia with an ANC nadir of 930/mm^3 on Study Day 14. Eight subjects developed mild cervical lymphadenopathy. Four vaccinees and 3 placebo recipients developed headache. The headaches were mild in 3 of the 4 vaccinees. Headache in the fourth vaccinee was moderate and occurred on Study Day 28. This headache was determined to be unlikely or unrelated to vaccine.

Virology: Fifteen of 20 vaccinees had vaccine virus recovered from the blood following Dose 1 (**Table 9**). One subject was viremic with 3 dengue vaccine viruses (DEN2/4Δ30(ME), DEN3Δ30/31, and DEN4Δ30). Three subjects were viremic with 2 dengue vaccine viruses; one with DEN1Δ30 and DEN3Δ30/31, one with DEN1Δ30 and DEN4Δ30, and one with DEN2/4Δ30(ME) and DEN4Δ30. Only 1 serotype of vaccine virus was recovered from the remaining 11 vaccinees. DEN3Δ30/31 was recovered from 8 subjects, DEN1Δ30 from 6 subjects, DEN4Δ30 from 5 subjects, and DEN2/4Δ30(ME) from 2 subjects.

Serology: Following a single vaccination, 100% of vaccinees became seropositive to DENV-1, 50% of vaccinees became seropositive to DENV-2, and 85% of vaccinees became seropositive to DENV-3 and 100% to DENV-4 (Table **11**). Seropositivity was defined as a 60 percent plaque reduction neutralization titer ($PRNT_{60}$) ≥ 1:10. Geometric mean peak titers are presented in

Table 12. Forty percent of vaccinees had a tetravalent response, 50% had a trivalent response, and 10% had a bivalent response.

Although each admixture induced at least a trivalent response in at least 75% of vaccinated adults, the TV003 admixture appeared to induce a broader response resulting in 90% of vaccinated adults achieving at least a trivalent response following a single dose. For this reason, TV003 is being further evaluated in this study.

2.4.2.4 Summary of clinical, virologic, and serologic response to TV005

Twenty subjects received admixture TV005 and 8 subjects received placebo. Following a single subcutaneous dose, the most common AE related to vaccine across all cohorts was an asymptomatic rash similar to that reported for the monovalent vaccines and headache. All of the tetravalent admixtures were well tolerated **(Table 8)**. TV005 (bolded in table) was well tolerated by the subjects. One vaccinee developed a fever on Study Days 8 – 9 post-vaccination (TMax 101.6°F), however this fever was unrelated to vaccine and was related to an episode of acute pelvic inflammatory disease (PID). The subject was treated for PID and all signs/symptoms resolved. Ten vaccinees developed a dengue vaccine virus-like rash. The day of onset of the rash ranged from Study Day 8 to Study Day 14. The rash was of mild severity in all subjects. One vaccinee developed a mild neutropenia (Study Day 16). Six vaccinees developed mild cervical lymphadenopathy. Ten episodes of headache were reported from 8 vaccinees and 7 episodes of headache were reported from placebo recipients. The headaches were mild in 7 of the 8 vaccinees; headache in the remaining vaccinee was moderate and occurred on Study Day 12.

Virology: Seventeen of 20 vaccinees had vaccine virus recovered from the blood following Dose 1 (**Table 10**). One subject was viremic with 3 dengue vaccine viruses (DEN2/4Δ30(ME), DEN3Δ30/31, and DEN4Δ30). Three subjects were viremic with 2 dengue vaccine viruses; one with DEN1Δ30 and DEN3Δ30/31, one with DEN1Δ30 and DEN4Δ30, and 1 with DEN2/4Δ30(ME) and DEN4Δ30. Only 1 serotype of vaccine virus was recovered from the remaining 11 vaccinees. DEN3Δ30/31 was recovered from 8 subjects, DEN1Δ30 from 6 subjects, DEN4Δ30 from 5 subjects, and DEN2/4Δ30(ME) from 2 subjects.

Serology: Following a single vaccination, 80% of vaccinees became seropositive to DENV-1, 60% of vaccinees became seropositive to DENV-2, and 80% of vaccinees became seropositive to DENV-3 and 100% to DENV-4 (**Table 11**). Seropositivity was defined as a $PRNT_{60}$ ≥1:10. Geometric mean peak titers are presented in

Table 12. Forty percent of vaccinees had a tetravalent response, 50% had a trivalent response, and 10% had a bivalent response.

Table 8: Clinical response to 5 tetravalent DEN vaccine candidate admixtures

Vaccine admixture	Dose of each component (log_{10})[1]	No. of subjects	% of subjects with indicated clinical sign/symptom				
			Fever	Rash	Headache	Neutropenia[2]	↑ALT
TV001	3333	20	0	65	45	25	0
TV002	3333	21	0	43	38	24	5
**TV003**	**3333**	**20**	**0**	**75**	**20**	**20**	**5**
TV004	3333	20	0	70	55	25	5
**TV005**	**3433**	**20**	**5**[3]	**50**	**40**	**5**	**0**
Placebo	n/a	40	0	0	38	13	3

1. The titer of each component of the administered tetravalent admixture was 3 log_{10} PFU with the exception of the DENV-2 component of TV005; the titer of DEN2/4Δ30 in TV005 was 4 log_{10} PFU.

Neutropenia was defined as an ANC ≤1,500/mm^3.

3. Fever was unrelated to vaccine. Subject had episode of acute pelvic inflammatory disease.

Table 9: Magnitude, onset, and duration of viremia in subjects[1] inoculated with TV003

Vaccine component	No. viremic (%)	Mean peak titer[2] ± SE	Mean day of onset of viremia (range)	Mean duration in days (range)
DEN1Δ30	6 (30)	0.7 ± 0.2	9.8 (8-12)	2.3 (1-5)
DEN2/4Δ30(ME)	2 (10)	0.5 ± 0.2	6.0 (all 6)	1.0 (all 1)
DEN3Δ30/31-7164	8 (40)	0.6 ± 0.1	8.2 (5-14)	2.6 (1-6)
DEN4Δ30	5 (25)	0.5 ± 0.1	7.4 (6-9)	1.4 (1-3)
	Total 15 (75)			

1. Number of subjects = 20
2. Titer is expressed as log_{10} PFU/mL.

Table 10: Magnitude, onset, and duration of viremia in subjects[1] inoculated with TV005

Vaccine component	No. viremic (%)	Mean peak titer[2] ± SE	Mean day of onset of viremia (range)	Mean duration in days (range)
DEN1Δ30	8 (40)	0.6 ± 0.05	13.6 (10-16)	1.4 (1-4)
DEN2/4Δ30(ME)	5 (25)	0.5 ± 0.06	9.0 (2-14)	2.4 (1-8)
DEN3Δ30/31-7164	8 (40)	0.6 ± 0.05	9.1 (6-12)	3.1 (1-8)
DEN4Δ30	9 (45)	0.5 ± 0.05	11.9 (6-16)	1.3 (1-3)
	Total 18 (90)			

1. Number of subjects = 20
2. Titer is expressed as $\log_{10}$ PFU/mL.

Table 11: Summary of serologic response induced by a single subcutaneous dose of TV003 or TV005

		% of vaccinees seroconverting to specific serotype[1]			
Admixture	N	DENV-1	DENV-2	DENV-3	DENV-4
TV003	20	100	50	85	100
TV005	20	80	60	90	100

[1] Seropositive = 60 percent plaque reduction neutralization titer ($PRNT_{60}$) of ≥1:10

Table 12: Geometric mean peak titers following vaccination with TV003 or TV005

Admixture	Geometric Mean Peak Titer (range)			
	**DENV-1**	**DENV-2**	**DENV-3**	**DENV-4**
TV003	62 (24-175)	44 (14-133)	36 (12-96)	66 (33-266)
TV005	40 (16-91)	44 (10-149)	35 (14-113)	70 (14-338)

Table 13: Percent and cumulative neutralizing antibody[1] responses following a single dose of TV003 or TV005

		% Vaccinees with multivalent response (cumulative)				
Admixture	N	Tetravalent	Trivalent	Bivalent	Monovalent	None
TV003	20	**40 (**40**)**	**50** (90)	**10** (100)	**0** (100)	**0**
TV005	20	**60** (60)	**30** / (90)	**0** / (90)	**10** / (100)	0

1. $PRNT_{60} \geq 1:10$

2.4.3 Mosquito Transmissibility of monovalent vaccine candidates

Both the wt rDENV-1 and rDEN1Δ30 demonstrated a low infectivity (50% mosquito infectious dose [MID_{50}] of >10^3 PFU/mL) (31). This is consistent with previously reported studies that demonstrated a very low infectivity of DENV-1 for *Aedes aegypti* fed an infectious blood meal. It is unlikely that the vaccine virus would be transmitted from an infected vaccinee to a mosquito since rDEN1Δ30 is poorly infectious for mosquitoes and because of the low level of viremia demonstrated in humans (9).

Transmissibility of rDEN2/4Δ30(ME) to both *Ae. aegypti* and *Toxorhynchites splendens* mosquitoes is reduced compared with both rDENV-4 and DENV-2-NGC (32). The minimum infectious dose of rDEN2/4Δ30(ME) needed to infect 50% of *Ae. aegypti* mosquitoes (MID_{50}) was ≥10-fold higher than that of either rDENV-4 or DENV-2-NGC, indicating impaired infectivity for the midgut. rDEN2/4Δ30(ME) was less infectious than either parent virus for *T. splendens*, indicating reduced ability of the chimeric virus to disseminate to the head of the mosquito. It is unlikely that the vaccine virus would be transmitted from an infected vaccinee to a mosquito since rDEN2/4Δ30(ME) is poorly infectious for mosquitoes and because of the low level of viremia demonstrated in humans (10).

rDEN2/4Δ30(ME) should be poorly transmitted to mosquitoes, as previously observed for the attenuated rDEN4Δ30 vaccine candidate, which was not transmitted from 10 infected vaccinees to over 300 feeding mosquitoes (8, 29). The parent virus of the rDEN3-3′D4Δ30 and rDEN3Δ30/31-7164 virus vaccines, DENV-3-Sleman/78, is very poorly transmitted to mosquitoes: ingestion by *Ae. aegypti* mosquitoes of $10^{4.1}$ PFU of wt DENV-3 Sleman/78 infected the midgut of only 4 of 28 (14%) mosquitoes tested and disseminated from the midgut in only 2 of 28 (7%) mosquitoes. The required dose of wt DENV-3 Sleman/78 is in excess of 10^5 PFU/mL in blood to allow for transmission to *Ae. aegypti* mosquitoes, the natural vector of DENV (4).

In addition, for dengue viruses to be transmitted from 1 person to another there is a 10 – 14 day incubation period within the mosquito. Therefore, for these vaccine viruses to be transmitted to an in-house family member, the subject would have to be viremic with a peak virus titer greater than 10^5 PFU/mL, would have to be bitten by a viable vector mosquito at the peak of viremia, the mosquito would have to live for an additional 10 – 14 days, and the same mosquito would then have to bite another family member. The peak virus titer of all the live attenuated dengue vaccine viruses tested thus far were at least 1000-fold below the viremia level required for transmission to mosquitoes.

The rDEN4Δ30 virus construct has been extensively studied with respect to its transmissibility to humans by mosquitoes (29). The rDEN4Δ30 was restricted both in its ability to infect the midgut and to cause a disseminated infection in mosquitoes. The ability of rDEN4Δ30 to be transmitted from viremic subjects to *Ae. albopictus* mosquitoes was also evaluated. Mosquitoes fed on subjects on Study Days 7, 8, and 9 post-infection. Vaccine virus was not recovered from any of the 352 mosquitoes that fed on the subjects even though some of the subjects were viremic on each of the days tested (29). The restricted capacity of rDEN4Δ30 to disseminate from the midgut of the mosquito to its head is specified by the Δ30 mutation.

The restricted capacity of rDEN4Δ30 to disseminate from the midgut of the mosquito to its head is specified by the Δ30 mutation, which is a mutation engineered into rDEN3-3´4Δ30. Ingestion by *Ae. aegypti* mosquitoes of $10^{4.1}$ PFU of wild type DENV-3 Sleman/78 infected the midgut of only 4 of 28 (14%) mosquitoes tested and disseminated from the midgut in only 2 of 28 (7%) mosquitoes. In addition, the low level of viremia in vaccinated subjects will preclude transmissibility to mosquitoes. rDEN3-3´D4Δ30 and rDEN3Δ30/31-7164 should be poorly transmitted to mosquitoes, as previously observed for the attenuated rDEN4Δ30 vaccine candidate, which was not transmitted from 10 infected vaccinees to over 300 feeding mosquitoes (8, 29).

2.4.4 Participation of Children

Although these vaccines have been evaluated in more than 300 healthy adults and well tolerated, it is felt that insufficient data are available to judge the potential risk in children. Once safety is established in adults in the United States and then in endemic regions, it is anticipated that future evaluation of these vaccines in children in dengue-endemic regions will be initiated.

3 Objectives

3.1 Primary Objectives

The primary objectives of this study are the following:

1. Determine the safety of TetraVax-DV TV003 and TV005 in persons with a documented history or evidence of flavivirus immunity prior to enrollment (flavivirus experienced), as assessed by the frequency of vaccine-related AEs, graded by severity.
2. Determine the immunogenicity of TV003 and TV005, as assessed by neutralizing antibody titers to DENV-1, DENV-2, DENV-3, and DENV-4 at 28, 56, 90, and 180 days after each vaccination. Monovalent, bivalent, trivalent, and tetravalent seropositivity rates will be determined at 28, 56, and 90 days post-each vaccination.
3. Determine if a second dose of TV003 or TV005 given at Study Day 180 will induce seropositivity in those vaccinees that remained seronegative to 1 or more DENV serotypes following the first vaccination.

3.2 Secondary Objectives

The secondary objectives of this study are the following:

1. Assess the frequency, quantity, and duration of viremia following vaccination.
2. Determine the number of flavivirus-experienced vaccinees infected with DENV-1, DENV-2, DENV-3, and DENV-4 by vaccination with TV003 or TV005. Infection is defined as recovery of vaccine virus from the blood or serum of a subject and/or by developing seropositivity to DEN virus ($PRNT_{50}$ ≥1:10).
3. Determine the duration of the neutralizing antibody response 26 weeks after each vaccination.

3.3 Exploratory Objectives

The exploratory objectives of this study are the following:

1. To evaluate the cellular immune response in flavivirus-experienced subjects following vaccination with TV003 or TV005.
2. To evaluate B and T cell memory responses in flavivirus-experienced subjects following vaccination with TV003 or TV005.
3. To evaluate the innate immune response in flavivirus-experienced subjects following vaccination with TV003 orTV005.

4 Study Design

4.1 Overall Design

The study is a placebo-controlled, double-blind study in normal healthy flavivirus-experienced adult subjects recruited from the metropolitan Baltimore/Washington, DC and areas in the state of Vermont. The trial will be conducted at the Center for Immunization Research in Baltimore, MD and at the University of Vermont, Burlington, VT. The purpose of this study is to evaluate the safety, reactogenicity, and immunogenicity of a single formulation of the live attenuated tetravalent dengue vaccine TetraVax-DV TV003 or TV005 when given as a subcutaneous injection to a person who has laboratory evidence or documented history of previous flavivirus infection. Subjects will be recruited from:

1.) Subjects who have failed screening for previous flavivirus vaccine trials because they were previously exposed to a flavivirus or were previously vaccinated with a flavivirus and/or
2.) Subjects who respond to an advertisement stating we are recruiting subjects who may have had dengue or other flavivirus infection.
3.) Subjects who have screened for other studies

TV003 will contain 10^3 PFU of rDENV-1Δ30, 10^3 PFU of rDEN2/4Δ30(ME), 10^3 PFU of rDEN3Δ30/31-7164 and 10^3 of rDEN4Δ30. TV005 will contain 10^3 PFU of rDENV-1Δ30, 10^4 PFU of rDEN2/4Δ30(ME), 10^3 PFU of rDEN3Δ30/31-7164 and 10^3 of rDEN4Δ30. Placebo controls are included in the study as a control to better assess vaccine-associated versus non-vaccine-associated AEs.

After providing written informed consent, subjects will undergo eligibility screening, including; medical history, physical examination, hematology testing, liver and renal function testing, human immunodeficiency virus (HIV) screening, hepatitis B and C screening, and urinalysis. Those subjects without documentation of previous flavivirus infection will have serum screened for neutralizing antibodies against DENV-1, DENV-2, DENV-3, DENV-4, WNV, SLE, TBEV, and YFV. Pregnancy testing will be performed on applicable female subjects. All screening tests must be performed within 60 days of vaccination.

All clinically significant abnormalities will be reviewed with subjects and referral for follow-up care will be provided. Subjects will be determined to be eligible based on the inclusion and exclusion criteria found in **Section 5** of this protocol. For subjects who are eligible, the Day 0 visit will be scheduled for the subjects to receive their first vaccination. Pregnancy

testing will be repeated on female subjects on the day of vaccination and during follow-up. Subjects will be considered enrolled in the study only after they are vaccinated (receive vaccine or placebo).

After vaccination, subjects will be evaluated in the clinic for at least 30 minutes and then return to the clinic on Study Day 3 post-vaccination. They will be seen every other day in the clinic from Study Day 8 through Study Day16 for evaluation by a clinician and to have blood drawn for clinical laboratory studies, virologic assays, and immunologic assays. They will also have a clinical evaluation performed at each specified visit (see **Section 7.4** for detailed description of study procedures). They will return to the clinic on Study Days 21, 28, 56, 90, and 150. On Study Day 180, subjects will return to the clinic for a second vaccination. They will receive the same test article that they received for their first vaccination. Follow-up after second vaccination will be the same as after first vaccination with the exception of the Study Day 150 visit. They will return to the clinic approximately 6 months following second vaccination (approximately 1 year after initial vaccination) for their final visit. All subjects will be provided with a memory enhancement card on which they will be asked to record their temperature 3 times a day for the first 16 days post-vaccination.

4.2 Dosing Strategy

Fifty-six subjects will receive 2 subcutaneous doses of the admixture of TetraVax-DV TV003 or TV005 vaccine (40 subjects each cohort) or placebo (16 subjects each cohort). Subjects will be followed for approximately 26 weeks after receiving the last dose of vaccine or placebo.

4.3 Sample Size and Placebo Ratio

A sample size of 40 vaccinees was chosen in order to detect small differences in the mean peak titers of each virus attained when flavivirus-experienced subjects were vaccinated compared with those flavivirus-naïve subjects who were vaccinated with the same formulation in another study (CIR 279). We hope to determine if pre-existing flavivirus antibody in these subjects will result in enhanced viremia following vaccination. A sample size of 40 vaccinees in each group will allow us to determine with 80% power an increase in virus titer of $\geq 0.3 \log_{10}$ PFU with an α value of 0.05. Placebo recipients are included in order to better discern adverse events related to vaccine from those adverse events that occur randomly and may not be related to vaccine. Trending of adverse events will be noted. One hundred and twelve subjects (80 vaccinees and 32 placebo recipients) will be enrolled in the study. Forty subjects in each cohort will be chosen to receive vaccine for the following reasons:

- This study is designed to build upon the previous Phase 1 studies of each monovalent candidate and the tetravalent admixtures, which used similar cohorts and the same vaccine to placebo ratio. We have increased the numbers of vaccine recipients to expand the safety evaluation of these admixtures.
- Placebo-recipients are included to better assess if common AEs are vaccine-related.

4.4 Duration of Subject Participation

Subjects will be followed for approximately 52 weeks (approximately 360 days) from the time of first vaccination. Subjects will be screened for eligibility up to 60 days prior to vaccination on Study Day 0.

4.5 Estimated Duration of the Study

The entire duration of the study is approximately 80 weeks, including approximately 8 weeks of screening prior to vaccination. The study will last for a total of approximately 52 weeks from the time the last subject is vaccinated.

4.6 Treatment Assignment

Subjects in the first cohort will be randomly assigned to receive TetraVax-DV TV003 vaccine or placebo. Subjects in the second cohort will be randomly assigned to receive TetraVax-DV TV005 or placebo. The TV003 cohort will be enrolled first. Treatment assignment will be done using a random number generator to prepare the sequence in which subjects are assigned to receive vaccine or placebo. Subjects will be randomized in blocks of 7 by the vaccine preparation study staff at JHSPH. There will be 8 blocks of 7 for each cohort; each block of 7 will include 5 vaccine recipients and 2 placebo recipients. Subjects will be enrolled sequentially. Once all 7 subjects in a block have reached Study Day 270, the block may be unblinded.

On the day of vaccination, once subjects have met all eligibility criteria, study staff will assign the subjects sequential ID study numbers from a prepared list. Clinical staff will remain blinded to treatment assignment until all subjects within a block have reached Study Day 270.

A master log of treatment assignments will be maintained in a record separate from other study records. This log will be kept in the CIR Laboratory at JHSPH. A copy of the treatment log will also be kept in the General Clinical Research Center (GCRC)/ Fletcher Allen Health Care (FAHC) Research Pharmacy (Vermont). At each location, the log will be kept in a locked room with limited access. A sealed envelope containing a copy of the treatment assignment will also be kept by the Data and Safety Monitoring Board (DSMB) Executive Secretary.

The subjects will be informed of their treatment assignment after they have reached Study Day 270.

4.7 Blinding

This study will be conducted as a double-blind study to avoid biased assessment of AEs. Vaccine or placebo will be prepared and drawn up into syringes by pharmacy/laboratory personnel who are not involved with the clinical assessment of subjects or performing research assays. Syringes are labeled according to SOPs/recommendations. Because vaccine diluent is used as placebo, there will be no difference in the appearance of the contents of the syringes.

The subject, investigator, and clinical staff will not know to which treatment group the subject has been assigned. In addition, other personnel assigned to monitor the study will not know the treatment assignment of the subject. Clinical staff will remain blinded to treatment assignment until all subjects within a block of 7 subjects have reached Study Day 270.

Routine unblinding will occur after the Principal Investigator (PI) requests treatment assignments in writing from the unblinded study staff.

If the need arises to unblind a specific subject's assignment for emergency medical management prior to all subjects within a block completing Study Day 270, the PI will contact CIR or GCRC research pharmacy unblinded study staff and obtain the treatment assignment of the subject in question. Only that specific subject's assignment will be unblinded. The Sponsor and the DSMB Executive Secretary will be notified of the event within 2 business days. This will also be documented in the subject's study chart.

5 Selection and Enrollment of Subjects

Subjects must meet all eligibility criteria to be enrolled in the study. Any enrolled participant who is noted to have received placebo at the time of the dose assignment unblinding may be offered the opportunity to re-enroll in a different cohort within the study. This enrollment is contingent on meeting all of the eligibility criteria. Subjects will not be allowed to enroll into the protocol more than twice.

5.1 Inclusion Criteria

All of the following criteria must be fulfilled for a subject to qualify for inclusion in this study:

1. Adult male or female between 18 and 50 years of age, inclusive.
2. Good general health as determined by physical examination, laboratory screening, and review of medical history.
3. Documented history or serologic evidence of previous dengue virus infection or other flavivirus infection (e.g., YFV, SLE, WNV, JE, or TBEV) or
    a. Documented previous receipt of a flavivirus vaccine (licensed or experimental).
4. Available for the duration of the study, approximately 26 weeks post-second vaccination.
5. Willingness to participate in the study as evidenced by signing the informed consent document.
6. Females Only: Female subjects of childbearing potential willing to use effective contraception for the duration of the trial. Reliable methods of contraception include: hormonal birth control, condoms with spermicide, diaphragm with spermicide, surgical sterilization, intrauterine device, and abstinence ($\geq$6 months since last sexual encounter). All female subjects will be considered having child-bearing potential except for those with hysterectomy, tubal ligation, tubal coil (at least 3 months prior to vaccination), or post-menopausal status documented as at least 1 year since last menstrual period.

5.2 Exclusion Criteria

A subject will be excluded from the study if any of the following criteria are met:

1. Females Only: Currently pregnant, as determined by positive β-human choriogonadotropin (HCG) test, breast-feeding.

2. Evidence of clinically significant neurologic, cardiac, pulmonary, hepatic, rheumatologic, autoimmune, or renal disease by history, physical examination, and/or laboratory studies.
3. Behavioral, cognitive, or psychiatric disease that in the opinion of the investigator affects the ability of the subject to understand and cooperate with the requirements of the study protocol.
4. Screening laboratory values of Grade 1 or above for absolute neutrophil count (ANC), ALT, and serum creatinine, as defined in this protocol.
5. Any other condition that in the opinion of the investigator would jeopardize the safety or rights of a subject participating in the trial or would render the subject unable to comply with the protocol.
6. Any significant alcohol or drug abuse in the past 12 months which has caused medical, occupational, or family problems, as indicated by subject history.
7. History of a severe allergic reaction or anaphylaxis.
8. Severe asthma (emergency room visit or hospitalization within the last 6 months).
9. HIV infection, by screening and confirmatory assays.
10. Hepatitis C virus (HCV) infection, by screening and confirmatory assays.
11. Hepatitis B virus (HBV) infection, by Hepatitis B surface antigen (HBsAg) screening.
12. Any known immunodeficiency syndrome.
13. Use of anticoagulant medications.
14. Use of corticosteroids (excluding topical or nasal) or immunosuppressive drugs within 42 days prior to or following vaccination. Immunosuppressive dose of corticosteroids is defined as ≥10 mg prednisone equivalent per day for ≥14 days.
15. Receipt of a live vaccine within 28 days or a killed vaccine within the 14 days prior to vaccination or anticipated receipt of any vaccine during the 42 days following vaccination.
16. Asplenia.
17. Receipt of blood products within the past 6 months, including transfusions or immunoglobulin or anticipated receipt of any blood products or immunoglobulin during the 42 days following vaccination.
18. Anticipated receipt of any investigational agent in the 42 days before or after vaccination.
19. Subject has definite plans to travel to a dengue endemic area during the study.
20. Refusal to allow storage of specimens for future research.

5.3 Additional Inclusion Criteria for Second Dose of Vaccine

1. Good general health as determined by physical examination and review of medical history.
2. Available for the duration of the study, approximately 6 months post-vaccination.
3. Females Only: Female subjects of childbearing potential willing to use effective contraception for the duration of the trial. Reliable methods of contraception include: hormonal birth control, condoms with spermicide, diaphragm with spermicide, surgical sterilization, intrauterine device, and abstinence (≥6 months since last sexual encounter). All female subjects will be considered having child-bearing potential except for those with hysterectomy, tubal ligation, tubal coil (at least 3 months prior to

vaccination), or post-menopausal status documented as at least 1 year since last menstrual period.

5.4 Exclusion Criteria for Second Dose of Vaccine

1. Anaphylaxis or angioedema following the first dose of vaccine.
2. Females Only: Currently pregnant, as determined by positive β- HCG test, breast-feeding.
3. Evidence of clinically significant neurologic, cardiac, pulmonary, hepatic, rheumatologic, autoimmune, or renal disease by history, physical examination, and/or laboratory studies.
4. Behavioral, cognitive, or psychiatric disease that in the opinion of the investigator affects the ability of the subject to understand and cooperate with the requirements of the study protocol.
5. Any other condition that in the opinion of the investigator would jeopardize the safety or rights of a subject participating in the trial or would render the subject unable to comply with the protocol.
6. Any significant alcohol or drug abuse in the past 12 months which has caused medical, occupational, or family problems, as indicated by subject history.
7. History of a severe allergic reaction or anaphylaxis.
8. Severe asthma (emergency room visit or hospitalization within the last 6 months).
9. HIV infection, by screening and confirmatory assays.
10. HCV infection, by screening and confirmatory assays.
11. HBV infection, by Hepatitis B surface antigen (HBsAg) screening.
12. Any known immunodeficiency syndrome.
13. Use of anticoagulant medications.
14. Use of corticosteroids (excluding topical or nasal) or immunosuppressive drugs within 42 days prior to or following vaccination. Immunosuppressive dose of corticosteroids is defined as ≥10 mg prednisone equivalent per day for ≥14 days.
15. Receipt of a live vaccine within 28 days or a killed vaccine within the 14 days prior to vaccination or anticipated receipt of any vaccine during the 42 days following vaccination.
16. Asplenia.
17. Receipt of blood products within the past 6 months, including transfusions or immunoglobulin or anticipated receipt of any blood products or immunoglobulin during the 42 days following vaccination.
18. Anticipated receipt of any other investigational agent in the 42 days before or after vaccination.
19. Subject has definite plans to travel to a dengue endemic area during the study.
20. Refusal to allow storage of specimens for future research.

5.5 Other Treatments and Ongoing Exclusion Criteria

The following criteria will be reviewed on Study Days 28 and 56 following each vaccination. If any become applicable during the study, the subject will not be included in further immunogenicity evaluations, as of the exclusionary visit. The subject will, however, be encouraged to remain in the study for safety evaluations for the duration of the study.

Ongoing Exclusion Criteria

1. Use of any investigational drug or investigational vaccine other than the study vaccine during the 42-day period post-vaccination.
2. Chronic administration (≥14 days) of steroids (defined as prednisone equivalent of ≥10 mg per day), immunosuppressants, or other immune-modifying drugs initiated during the 42-day period post-vaccination (topical and nasal steroids are allowed).
3. Receipt of a licensed vaccine during the 42-day period post vaccination.
4. Receipt of immunoglobulins and/or any blood products during the 42-day period post-vaccination.
5. Pregnancy

5.6 Subject Withdrawal and Termination Criteria

A subject will not be considered to have completed the trial if any of the following reasons apply. However, any subject who has received vaccine or placebo will be encouraged to remain in the study for periodic safety evaluations for the duration of the study at the discretion of the investigator.

1. ***Research terminated by Sponsor or Investigator*** – applies if the entire study is terminated by the Sponsor or investigator for any reason.
2. ***Withdrawal of consent*** – applies to a subject who withdraws consent to participate in the study for any reason.
3. ***Noncompliant with protocol*** – applies to a subject who does not or is not able to comply with protocol-specific visits or evaluations on a consistent basis such that adequate follow-up is not possible and the subject's safety would be compromised by continuing in the trial.
4. ***Subject withdrawal*** may occur if the investigator believes that it is in the best interest of the subject to be withdrawn from the study.
5. ***Other*** – is a category used when previous categories do not apply and requires an explanation.

Special Situations:

<u>Pregnancy</u>:
If a subject becomes pregnant during the study, the subject will not be included in the immunogenicity evaluations from her estimated date of conception. She will, however, be encouraged to remain in the study for periodic safety evaluations and will be followed until completion of her pregnancy. The subject will be asked to sign a release of medical information form so that records can be obtained from her obstetrician regarding the outcome of the pregnancy.

<u>Lost to follow-up</u>:
A subject who is not reachable by telephone or other means of communication and therefore not able to be located is considered lost to follow-up. A subject may be considered lost to follow-up and withdrawn from the study once 3 attempts have been made to contact the subject, followed by nonresponse to a certified letter to the last known address requesting that the subject contact the clinical site.

Incarceration:
In the event that a subject becomes incarcerated during the course of the study, every attempt will be made to contact the subject for determination of his/her safety. A subject may be terminated from the study if his/her period of incarceration will make him/her unable to make scheduled visits.

5.7 Access to Medical Records

The medical history of a subject will be obtained from the subject and recorded. Medical records from an outside institution will not be requested unless there is a need to clarify the subject's medical history. Medical records from an outside institution will not be requested without the medical release form signed by the subject.

6 Vaccine Preparation

6.1 Pre-Vaccination Preparation

Monovalent vaccine virus for this protocol will be stored at a NIAID-contracted repository until requested by the clinical site. Vials of frozen vaccine for administration will be formally requested to be transferred to the clinical site by the PI/designee after Institutional Review Board (IRB) approval for the study has been granted and the Food and Drug Administration (FDA) has been in receipt of the protocol for at least 30 days without issuing a clinical hold. Vaccine may be transferred to the study site prior to IRB approval for the purpose of determining the titer of the vaccine only.

After transfer to the clinical site, each monovalent vaccine will be stored in a locked freezer at -80 ± 15°C until time of use. The vaccine is supplied as a concentrate that must be diluted to the proper dose prior to administration.

Clinical site pharmacy/lab personnel who are not blinded to treatment assignment will be responsible for preparing the vaccine and placebo. The tetravalent admixture of vaccine will be prepared as a site-of-injection formulation. Prior to vaccination, the PI/designee will supply a prescription request form which will include the protocol number, date of vaccination, dose, route of administration, the vaccine virus name, vaccine lot number, the vaccine titer (concentration), the investigational new drug (IND) number, the number of doses to be administered, and the dilution instructions for the vaccine to the study staff responsible for vaccine preparation.

Admixture TV003 (10^3 of rDEN1Δ30, 10^3 of rDEN2/4Δ30(ME), 10^3 of rDEN3Δ30/31-7164, and 10^3 of rDEN4Δ30) and admixture TV005 (10^3 of rDEN1Δ30, 10^4 of rDEN2/4Δ30(ME), 10^3 of rDEN3Δ30/31-7164, and 10^3 of rDEN4Δ30) will be prepared according to the site's Standard Operating Procedure (SOP). Study staff will prepare the correct dose of vaccine (or placebo) for each subject in a biosafety cabinet using aseptic technique. Vaccine will be diluted with 1X Leibovitz L-15 medium, which is prepared from a specific safety tested lot of 2X L-15 medium and sterile water for injection (1:1, v/v). A Type II Master File for L-15 was created and submitted to the FDA (BB-MF #12959) on 27 February 2006. The testing results for the current lot of 2X Leibovitz L-15 medium is available in the Master File. The diluted vaccine (or placebo) will be drawn up to a volume of 0.5 mL in a 1 mL syringe and labeled according to SOP. The labeled syringes will be transported on wet ice to the clinic

for administration. Vaccine must be used within 4 hours of being removed from the freezer. After the tetravalent admixture has been prepared at the clinical site(s), thawed undiluted (if sufficient quantities remain in the separate monovalent vaccine vials) and the final diluted tetravalent admixture will be titrated for each individual vaccine virus component.

Placebo for this study will be the vaccine diluent, 1X L-15. Placebo is prepared from a specific safety tested lot of double-strength (2X) L-15 medium (BB-MF #12959) that is obtained from a NIAID contracted repository and mixed 1:1 with sterile water for injection.

6.2 Vaccine Storage

Each monovalent vaccine candidate should remain frozen at -80 ± 15°C until just prior to use. Vaccine should never be refrozen for reuse in vaccine preparation. Diluent components will be stored at 2°C to 8°C as per the manufacturer's recommendation. Vaccine and diluent components should be opened from new containers for each use. No component should be reused for future vaccine or placebo preparation.

6.3 Vaccine Accountability

Laboratory vaccine preparation personnel will maintain an accurate inventory and accountability record of vaccine for this study. Partially used vials of vaccine or placebo components will not be refrozen or reused for future vaccinations.

6.4 Storage Disposition of Used/Unused Supplies

After laboratory personnel have diluted the vaccine and drawn up the syringes for administration, they will remove the label from the vaccine vial and place it in the vaccine preparation form. In this manner, monitoring personnel will be able to verify the accountability of all vaccine vials used for the study. In addition, the number of vaccine vials used will be accounted for in the study specific drug accountability log. The diluent will be accounted for in the general accountability log, with identification of the specific study.

An aliquot of undiluted (if available) and diluted vaccine will be titrated by laboratory personnel after vaccine has been prepared and delivered to the clinical staff. This is done to confirm the potency of vaccine administered to the subjects.

At least 1 aliquot (if available) of diluted vaccine will also be frozen and stored at -80 ± 15°C at the clinical site for future re-titration if needed. After the syringes have been dispensed and aliquots removed for titration, any remaining vaccine will be destroyed by the laboratory personnel per SOP. Any unused diluent/placebo from opened vials/bottles will also be destroyed.

7 Study Procedures

The following sections provide a detailed listing of the procedures and studies to be performed in this protocol at designated time points.

7.1 Recruitment and General Screening

Subjects may be recruited from a variety of sources including, but not limited to, subjects previously enrolled in vaccine trials at the clinical sites, the use of a center wide IRB

approved screening protocol, and/or by the use of study-specific IRB-approved print, electronic and/or other media advertising.

After an initial phone screen (using IRB approved Phone Screen/Initial Contact form) by clinic staff focused on providing background information of the trial and a review of basic inclusion and exclusion criteria, a screening visit may be scheduled.

To assure that adequate time is given for the subject to consider study participation, a read-only copy of the IRB approved ICF may be sent via postal service, email, and/or fax prior to the screening visit. During the screening process, which may require more than 1 visit, the subject will read the consent form, be encouraged to ask questions, and then complete a comprehension assessment. Study staff will review and discuss the answers from the assessment to identify those areas of the informed consent form that need further review with the subject. This procedure will help ensure that the subject has sufficient understanding of the study before the consent form is signed. The subject may either sign the consent form during the screening visit or return after further consideration. The subject may have a medical history review, pregnancy prevention counseling, physical exam and/or lab specimens drawn during the general screening process.

7.2 Screening Procedures

Subjects will undergo the following screening procedures within 60 days of vaccination:

.

1. Ensure that HIV pre-test counseling has been performed and ensure that the subject has agreed to HIV testing (required by MD state law only).
2. Elicit a complete medical history, including menstrual and contraceptive history and/or history of surgical sterilization for female subjects.
3. Perform a complete physical examination, including vital signs (height, weight, blood pressure, respiration, and pulse).
4. Obtain approximately 40 mL of blood for the following laboratory screening tests:
   - Prothrombin time/partial thromboplastin time (PT/PTT)
   - Complete blood count (CBC) with differential
   - Alanine aminotransferase (ALT)
   - Aspartate aminotransferase (AST)
   - Total bilirubin
   - Previous flavivirus infection (if not already documented)
   - Creatinine
   - Alkaline phosphatase
   - HBV
   - HCV
   - HIV
5. Obtain urine for urine dipstick testing (glucose, bilirubin, ketones, specific gravity, blood, pH, protein, urobilinogen, nitrite, leukocyte esterase).
6. Obtain either urine or serum for β-HCG testing in females. A positive β-HCG will exclude the subject from the trial.
7. Counsel females to avoid becoming pregnant during the study.

7.3 Immunization Procedure

Subjects will receive vaccine or placebo on Study Day 0 and Day 180.

Monovalent vaccine candidates will be kept frozen at -80 ± 15°C until just before use, whereupon they will be thawed, diluted, combined into a tetravalent admixture, and drawn up for administration (see **Section 6** for vaccine preparation). Vaccine or placebo will be kept on wet ice from the time it is diluted until it is delivered to clinical staff for administration. A vaccine volume of 0.5 mL will be delivered by subcutaneous injection in the deltoid region of the upper arm with a needle of appropriate gauge and length after wiping the injection site with alcohol.

7.4 Detailed Study Procedures

The study procedures to be performed at each visit are listed below. Photographs may be taken of the injection site. In addition, photographs may be taken of other areas of the skin before and/or after vaccination to record the characteristics of any rash that may develop. The total volume of blood to be drawn over each 26-week post-vaccination period is approximately 435 mL, about the same as donating a unit of blood. This amount is well within NIH guidelines (Medical Administrative Policy 95-9) of blood donation and should not compromise the health of study subjects. The total amount of blood drawn over the 52-week duration of the study is approximately 870 mL.

7.4.1 First Vaccination

On the day of first vaccination, more subjects may be invited to the clinic than will be vaccinated. These subjects will be alternates and will be vaccinated only if other subjects are not available for vaccination or are found to be ineligible on the day of vaccination. Those subjects who are alternates will be informed that they are alternates when they are invited to the clinic.

Study Day 0 (Day of Vaccination)

1. Verify that Informed Consent was obtained and that the consent form was signed by both the subject and by the study staff.
2. Verify that all applicable eligibility criteria have been met.
3. Perform interim history and focused physical exam, concentrating on any acute complaints.
4. Obtain approximately 70 mL of blood for CBC with differential, PT/PTT, ALT, creatinine, virology, and immunology. These laboratory studies are drawn as baseline values and will not determine eligibility.
5. For applicable females, perform β-HCG testing. Ensure the test is negative before proceeding; a positive test will exclude the subject from the study as per protocol, **Section 5.4**. Review pregnancy prevention with the subject.
6. Record vital signs (blood pressure, temperature, heart rate, and respiratory rate).
7. Administer the vaccine.
8. Observe for at least 30 minutes after vaccination and evaluate for immediate hypersensitivity.
9. Provide education by study staff describing proper use of thermometer, the signs and symptoms of potential AEs, and how and when to contact study staff.

Study Day 3

1. Perform interim history and focused physical exam, concentrating on any acute complaints.
2. Record vital signs.
3. Obtain approximately 25 mL of blood for CBC with differential and immunology.

Study Day 8 (± 1 day)

1. Perform interim history and focused physical exam, concentrating on any acute complaints.
2. Record vital signs.
3. Obtain approximately 45 mL of blood for CBC with differential, ALT, PT/PTT, virology and immunology.

Study Day 10 (± 1 day)

1. Perform interim history and focused physical exam, concentrating on any acute complaints.
2. Record vital signs.
3. Obtain approximately 20 mL of blood for CBC with differential, ALT, and virology.

Study Day 12 (± 1 day)

1. Perform interim history and focused physical exam, concentrating on any acute complaints.
2. Record vital signs.

   Obtain approximately 55 mL of blood for CBC with differential, PT/PTT, ALT, creatinine, virology, and immunology.

Study 14 (± 1 day)

1. Perform interim history and focused physical exam, concentrating on any acute complaints.
2. Record vital signs.
3. Obtain approximately 20 mL of blood for CBC with differential, ALT, and virology.

Study Day 16 (± 1 day)

1. Perform interim history and focused physical exam, concentrating on any acute complaints.
2. Record vital signs.
3. Obtain approximately 25 mL of blood for CBC with differential, PT/PTT, ALT, and virology.

Study Day 21 (± 1 day)

1. Perform interim history and focused physical exam, concentrating on any acute complaints.
2. Record vital signs.
3. Obtain approximately 45 mL of blood for immunology/virology. Virology will be done only if sample from Study Day 16 was positive for vaccine virus. CBC, PT/PTT,

and ALT will be done if indicated (if values from Study Day 16 were Grade 1 or higher, as protocol defined).

Study Day 28 (± 2 days)

1. Perform interim history and focused physical exam, concentrating on any acute complaints.
2. Record vital signs.
3. Obtain approximately 30 mL of blood for immunology.
4. For females, perform β-HCG testing. A positive test will exclude the subject from the study as per protocol, **Section 5.4**. Review pregnancy prevention with the subject.

Study Day 56 (± 7 days)

1. Perform interim history for interim complaints (physical exam is optional).
2. Record vital signs.
3. Obtain approximately 30 mL of blood for immunology.
4. For females, perform β-HCG testing. A positive test will exclude the subject from the study as per protocol, **Section 5.4**. Review pregnancy prevention with the subject.

Study Day 90 (+ 10 days)

1. Perform interim history and focused physical examination, concentrating on any acute complaints.
2. Record vital signs.
3. Obtain approximately 30 mL of blood for immunology.
4. Females will have β-HCG testing. A positive test will exclude the subject from the study as per protocol, **Section 5.4**. Review pregnancy prevention with the subject.

Study Day 150 (+ 21 days)

1. Perform interim history and focused physical examination, concentrating on any acute complaints.
2. Record vital signs.
3. Obtain approximately 10 mL of blood for Hepatitis B, Hepatitis C, and HIV testing.
4. Obtain approximately 30 mL of blood for immunology.
5. Females will have β-HCG testing. A positive test will exclude the subject from the study as per protocol, **Section 5.4**. Review pregnancy prevention with the subject.

7.4.2 Second Vaccination

Study Day 180 (+35 Days): Day of Second Vaccination

1. Verify that all applicable eligibility criteria have been met.
2. Perform interim history and focused physical exam, concentrating on any acute complaints.
3. Obtain approximately 70 mL of blood for CBC with differential, PT/PTT, ALT, creatinine, virology, and immunology. These laboratory studies are drawn as baseline values and will not determine eligibility.
4. For applicable females, perform β-HCG testing. Ensure the test is negative before proceeding; a positive test will exclude the subject from the study as per protocol, **Section 5.4**. Review pregnancy prevention with the subject.

5. Record vital signs (blood pressure, temperature, heart rate, and respiratory rate).
6. Administer the vaccine.
7. Observe for at least 30 minutes after vaccination and evaluate for immediate hypersensitivity.
8. Provide education by study staff describing proper use of thermometer, the signs and symptoms of potential AEs, and how and when to contact study staff.

Study Day 183 (3 days following second vaccination)
1. Perform interim history and focused physical exam, concentrating on any acute complaints.
2. Record vital signs.
3. Obtain approximately 25 mL of blood for CBC with differential and immunology.

Study Day 188 (± 1 day from 8 days following second vaccination)
1. Perform interim history and focused physical exam, concentrating on any acute complaints.
2. Record vital signs.
3. Obtain approximately 45 mL of blood for CBC with differential, ALT, PT/PTT, virology, and immunology.

Study Day 190 (± 1 day from 10 days following second vaccination)
1. Perform interim history and focused physical exam, concentrating on any acute complaints.
2. Record vital signs.
3. Obtain approximately 20 mL of blood for CBC with differential, ALT, and virology.

Study Day 192 (± 1 day from 12 days following second vaccination)
1. Perform interim history and focused physical exam, concentrating on any acute complaints.
2. Record vital signs.
3. Obtain approximately 55 mL of blood for CBC with differential, PT/PTT, ALT, creatinine, virology, and immunology.

Study Day 194 (± 1 day from 14 days following second vaccination)
1. Perform interim history and focused physical exam, concentrating on any acute complaints
2. Record vital signs.
3. Obtain approximately 20 mL of blood for CBC with differential, ALT, and virology.

Study Day 196 (± 1 day from 16 days following second vaccination)
1. Perform interim history and focused physical exam, concentrating on any acute complaints.
2. Record vital signs.
3. Obtain approximately 25 mL of blood for CBC with differential, PT/PTT, ALT, and virology.

Study Day 201 (± 1 day from 21 days following second vaccination)

1. Perform interim history and focused physical exam, concentrating on any acute complaints.
2. Record vital signs.
3. Obtain approximately 45 mL of blood for immunology/virology. Virology will be done only if sample from Study Day 196 was positive for vaccine virus. CBC, PT/PTT, and ALT will be done if indicated (if values from Study Day 196 were Grade I or higher, as protocol defined).

Study Day 208 (± 2 days from 28 days following second vaccination)

1. Perform interim history and focused physical exam, concentrating on any acute complaints.
2. Record vital signs.
3. Obtain approximately 30 mL of blood for immunology.
4. For females, perform β-HCG testing. A positive test will exclude the subject from the study as per protocol, **Section 5.4**. Review pregnancy prevention with the subject.

Study Day 236 (± 7 days from 56 days following second vaccination)

1. Perform interim history for interim complaints. (Physical exam is optional)
2. Record vital signs.
3. Obtain approximately 30 mL of blood for immunology.
4. For females, perform β-HCG testing. A positive test will exclude the subject from the study as per protocol, **Section 5.4**. Review pregnancy prevention with the subject.

Study Day 270 (+10 days from 90 days following second vaccination)

1. Perform interim history for interim complaints. (Physical exam is optional)
2. Record vital signs.
3. Obtain approximately 30 mL of blood for immunology.
4. For females, perform β-HCG testing. A positive test will exclude the subject from the study as per protocol, **Section 5.4**. Review pregnancy prevention with the subject.

Study Day 360 (+ 28/-14 days from 180 days following second vaccination)

1. Perform interim history and focused physical examination, concentrating on any acute complaints.
2. Record vital signs.
3. Obtain approximately 30 mL of blood for immunology.
4. Females will have β-HCG testing.

7.5 Subject Temperature Memory Card

Subjects will be provided a thermometer and a "Temperature Memory Card", to use as a memory aid and will be asked to record temperatures 3 times a day from Study Day 0 through Study Day 16 post-vaccination. During post-vaccination visits (Study Day 3 through 28) staff will review the subjects' temperature cards to assess for any fevers and compare subject recordings to the study visit temperatures. Staff will record all temperatures recorded by the subject and will also separately record the maximum temperature for each date (T_{max}) on the

source document. Subject memory cards will not be collected by the study staff as subject's recordings will not be considered required data.

Staff will instruct subjects how to use the thermometers, to take their temperatures at approximately the same times each day, and to take additional temperatures if they feel they have elevated temperatures. Subjects will be asked to wait at least 15 minutes after eating, drinking, and smoking before taking their temperatures. They will be asked to confirm an elevated temperature (≥100.4°F) by retaking the temperature after a 20 minute interval and at 1 hour. Temperatures not documented to last at least 1 hour will not be considered an AE or included in the analysis.

Following second vaccination, subjects will be provided another thermometer and a "Temperature Memory Card", to use as a memory aid and will be asked to record temperatures 3 times a day from Study Day 180 through Study Day 196. During post vaccination visits (Study Day 183 through 208), staff will review the subjects' temperature cards to assess for any fevers and compare subject recordings to the study visit temperatures. Staff will record all temperatures recorded by the subject and will also separately record the maximum temperature for each date (T_{max}) on the source document. Subject memory cards will not be collected by the study staff as subject's recordings will not be considered required data.

7.6 Clinical Laboratory Testing

Using standard techniques, Quest Diagnostics, or other Clinical Laboratory Improvement Amendments (CLIA) certified laboratories will perform the following tests:

1. CBC plus white blood cell differential.
2. PT/PTT.
3. ALT, AST, alkaline phosphatase, total bilirubin, creatinine.
4. HIV assay (screening antibody assay with Western blot confirmation for positive antibody assays).
5. Hepatitis B and C screening.
6. Urinalysis (in the event of an abnormal urine dipstick test).
7. Serum β-HCG, if required.

Urine and serum β-HCG testing will be performed at the clinical trial site using an FDA-approved pregnancy test kit. Urine dipstick testing will be performed at the clinical trial site using an FDA-approved product. Determination of vaccine virus titer, plaque reduction neutralization antibody assays, and cellular immune studies will be done at the clinical trial site laboratory.

7.7 Medical History and Concomitant Medications

A complete medical history will be collected during screening. Any changes reported in medical history during each 28-day post vaccination period will be assessed as possible AEs. After Study Day 28 following each vaccination, the medical history will be updated for any new or changed significant or chronic conditions.

Study staff will collect current medications as part of the medical history, including over the counter medications and herbal supplements, at the time of enrollment. All changes or updates to medications will be collected through Study Day 28 following each vaccination. After Study Day 28, concomitant medications will be collected to identify new or changed significant or chronic conditions.

Medications taken for AEs continuing after Study Day 28 following each vaccination will be recorded throughout the trial.

7.8 Immunology Testing

7.8.1 Antibody Testing

Serum antibody levels to DENV-1, DENV-2, DENV-3, and DENV-4 will be measured by plaque reduction neutralizing antibody assay using standard laboratory protocols. The $PRNT_{50}$ is defined as the highest dilution of antibody that reduces the number of foci or plaques by 50%, compared to the plaque titer of a mixture of virus with serum from the same subject prior to vaccination.

7.8.2 Other Immunological Assays

Cytokine, and T-cell and B-cell stimulation assays, and peripheral blood mononuclear cell (PBMC) phenotyping may be performed on peripheral blood mononuclear cells. RNA may be isolated from cells and run on microarrays to identify immune pathways of interest such as defining the innate immune response to vaccine virus. In addition, human leukocyte antigen (HLA) typing of samples may be performed as part of assays to map the dengue virus epitopes that induce T or B cell responses.

7.9 Retention of Study Subjects

We will employ several strategies aimed at retaining participants through study completion. During screening, we will obtain detailed primary locator information, as well as secondary contact information. Subjects will also provide information for people who may be contacted if primary and secondary means of contact fail. Locator information will be reviewed with subjects at each visit (i.e., addresses, phone numbers, email addresses). In addition, birthday cards/holiday cards may be mailed to check addresses, and reminders may be sent using various methods (including but not limited to phone, email, text messaging, and postal mail). All data will be maintained and updated in a password protected locator database.

8 Adverse Event Monitoring

8.1 Definitions

8.1.1 Adverse Event

Any untoward or unfavorable medical occurrence in a human subject, including any abnormal sign (e.g., abnormal physical exam or laboratory finding), symptom, or disease, temporally associated with the subject's participation in the research, whether or not considered related to the research.

All AEs will be evaluated for severity, action taken, seriousness, outcome and relationship to the investigational vaccine as described in **Section 8.2** in this protocol.

If a diagnosis is clinically evident, the diagnosis rather than the individual signs and symptoms or lab abnormalities will be recorded as the AE. AEs will be collected through the 28-day period following each vaccination and any vaccine-related AEs identified in the 28-day post-vaccination period will be followed until resolution.

AEs are categorized as **solicited AEs and other AEs**. Solicited AEs include local reactogenicity, systemic reactogenicity, and laboratory events. Solicited AEs are those events which the clinician is specifically evaluating during each 28 day post-vaccination period, as listed in **Table 14.**

All AEs are evaluated using the Adverse Event Grading Table in **Appendix 1**.

Table 14: Solicited Adverse Events

Systemic Reactogenicity	Laboratory Events	Local Reactogenicity
-Fever	-Hemoglobin	-Injection site pain
-Headache	-ANC	-Injection site erythema
-Retro-orbital pain	-ALT	-Injection site swelling
-Photophobia	-Platelets	-Injection site tenderness
-Nausea	- White blood count (WBC)	-Injection site induration
-Fatigue	-Creatinine	-Injection site pruritus
-Myalgia	-PT	
-Arthralgia	-PTT	
-Dengue vaccine-like rash		

8.1.2 Serious Adverse Event

An SAE is an AE that is determined to be "serious" whether considered related to the investigational vaccine or not. SAEs will be collected for the duration of the trial. An SAE results in 1 or more of the following outcomes:

- Death during the period of protocol-defined surveillance.
- Life threatening event, defined as an event that places a subject at immediate risk of death at the time of the event and does not refer to an event that hypothetically might have caused death were it more severe.

- Inpatient hospitalization or prolongation of existing hospitalization, defined as at least an overnight stay in the hospital or emergency ward for treatment that would have been inappropriate if administered in the outpatient setting.
- Congenital anomaly or birth defect.
- Persistent or significant incapacity or substantial disruption of the ability to conduct normal life functions.
- Other medically important event*

  *Medical and scientific judgment should be exercised in deciding whether expedited reporting is appropriate in other situations, such as important medical events that may not be immediately life threatening or result in death or result in hospitalization but may jeopardize the subject or may require intervention to prevent one of the outcomes listed above. These will also usually be considered serious.

  Each AE will be classified by the investigator/designee as "serious" or "nonserious". An SAE needs to meet only one or more of the above criteria to be considered serious.

8.1.3 Unexpected Adverse Events

An AE is considered unexpected if it is not listed in the Investigator Brochure or Package Insert (for marketed products) or is not listed at the specificity or severity that has been observed. "Expected" does not mean that the event is expected with pharmacologically similar drugs, the underlying disease(s) or concomitant medications. It is the responsibility of the IND Sponsor to make this determination.

8.1.4 Suspected and Unexpected Serious Adverse Reaction (SUSAR)

A SUSAR is a Suspected Adverse Reaction that is both Serious and Unexpected.

8.1.5 Unanticipated Problems

An Unanticipated Problem (UP) is any incident, experience, or outcome that is

1. unexpected in terms of nature, severity, or frequency in relation to
   a. the research risks that are described in the IRB-approved research protocol and informed consent document; Investigator's Brochure or other study documents; and
   b. the characteristics of the subject population being studied; and
2. possibly, probably, or definitely related to participation in the research; and
3. places subjects or others at a greater risk of harm (including physical, psychological, economic, or social harm) than was previously known or recognized. (An AE with a serious outcome will be considered increased risk.)

8.1.6 Pre-existing Conditions, Worsening of Pre-existing Condition

Stable chronic conditions which are present prior to enrollment and do not worsen are not considered AEs and will be accounted for in the subject's medical history. Exacerbation or worsening of pre-existing conditions are defined as AEs and are evaluated using the same criteria described in **Section 8.2** in this protocol.

8.2 Assessment of Adverse Events

8.2.1 Identification of Adverse Events

Assessment of safety will include clinical observations and monitoring of hematological, blood chemistry, and immunologic parameters. Safety will be evaluated by monitoring of the subjects for local and systemic adverse events during the course of the trial. Subjects will be closely monitored for 30 minutes following each immunization. Additionally, subjects will return to the clinic on Study Days 3, 8, 10, 12, 14, 16, 21, 28, and 56 post-vaccination at a minimum and may be asked to return more often if warranted. It is during this time period that we anticipate AEs related to infection with the vaccine virus will manifest themselves, and hence, the subjects will be seen frequently. Study staff will review subject's reported temperatures and clinical temperatures for the first 16 days post vaccination to assess for AEs. At each visit through Study Day 28 following each vaccination, they will be queried about possible vaccine related AEs (solicited AEs) and will have a focused physical exam performed. A study clinician will be available to subjects by telephone or pager 24 hours a day during the study evaluation period.

All AEs will be recorded during the period after the subject receives the study vaccine, through and including post-vaccination Study Day 28.

All SAEs will be reported following SAE reporting guidelines outlined in **Section 8.3.2** of this protocol.

8.2.2 Protocol Specific Adverse Event Definitions

Dengue Vaccine-like Rash: macular/maculo-papular rash, typically found on the trunk and proximal extremities most frequently seen 10-16 days after vaccination.
Headache: a pain located in the head, over the eyes, at the temples, or at the base of the skull.
Retro-orbital pain (ROP): bilateral pain situated behind the orbits of the eye.
Photophobia: an abnormal sensitivity to or intolerance of light.
Nausea: discomfort in the stomach with an urge to vomit.
Fatigue: excessive tiredness following minimal exertion.
Myalgia: pain in the muscles, found in $\geq$ 2 muscle groups.
Arthralgia: pain in a joint, found in $\geq$ 2 joints.

8.2.3 Determination of Severity

The investigator/designee will assess all AE severity using the following classifications.

Table 15: Severity Definitions

Severity	Defined
Grade 1 (Mild)	Event that is easily tolerated, may require 1 dose of medication/treatment
Grade 2 (Moderate)	Event that interferes with daily activity or requires more than 1 dose of medication/treatment
Grade 3 (Severe)	Event that prevents daily activity and requires medical intervention

Solicited AE severity grading classifications are listed in **Table 16**. All other AEs will be graded in severity using the Adverse Event Grading Table in **Appendix 1**.

Table 16: Assessment of Solicited Adverse Events

Local Reactogenicity	Grade	Severity
Injection Site Tenderness	1	Event that is easily tolerated
Injection Site Pain	2	Event that interferes with daily activity
Injection Site Pruritus	3	Event that prevents daily activity
Injection Site Induration	1	>0 - 20 mm
Injection Site Erythema	2	>20 - 50 mm
Injection Site Swelling	3	>50 mm
**Systemic Reactogenicity**	**Grade**	**Severity**
Fever (oral)	1	100.4° F – 101.4°F
	2	101.5°F – 102.4°F
	3	≥102.5°F
Dengue Vaccine Like Rash	1	Rash is present but asymptomatic
	2	Rash is symptomatic (pruritus/pain) but does not interfere with function
	3	Rash is symptomatic and interferes with function
Headache<br>Retro-orbital pain (ROP)<br>Photophobia<br>Nausea<br>Fatigue<br>Myalgia<br>Arthralgia	1	Event that is easily tolerated, may require 1 dose of medication/treatment
	2	Event that interferes with daily activity or requires more than 1 dose of medication/treatment
	3	Event that prevents daily activity and requires medical intervention
**Solicited Laboratory AEs**	**Grade**	**Severity**
Hemoglobin (women)	1	9.5 - 10.7 gm/dL
	2	8.0 - 9.4 gm/dL
	3	≤7.9 gm/dL
Hemoglobin (men)	1	11 – 12.5 gm/dL
	2	9.0 – 10.9 gm/dL
	3	≤8.9 gm/dL
Neutropenia (reduced ANC)	1	750 - 999/mm^3
	2	500 – 749/mm^3
	3	<500 mm^3
Leukocytosis (Increased WBCs)	1	11,500 - 13,000/mm^3
	2	13,001 - 15,000/mm^3
	3	>15,000 or <1,000/mm^3
Thrombocytopenia (Decreased Platelets)	1	≥100,000 – 120,000/mm^3
	2	≥75,000 - 99,999/mm^3
	3	≤74,999/mm^3
PT	1	>1.0 - 1.25 x ULN
	2	>1.25 - 1.5 x ULN
	3	>1.5 x ULN
PTT	1	>1.0 - 1.66 x ULN
	2	>1.66 - 2.33 x ULN
	3	>2.33 x ULN
ALT	1	>1.25 - 2.5 x ULN
	2	>2.5 - 5.0 x ULN
	3	>5.0 x ULN
Creatinine	1	1.5 – 1.7 mg/dL
	2	>1.7 – 2.0 mg/dL
	3	>2.0 mg/dL

8.2.4 Relationship with Receipt of Study Vaccine

The clinical investigator will assess all AEs for their relationship to the vaccine using the following classifications:

Definitely related:	Clear-cut temporal association, and no other possible cause
Probably related:	Reasonable temporal association and a potential alternative etiology is not apparent
Possibly related:	Less clear temporal association; other etiologies also possible
Unlikely related:	Temporal association between the AE and the vaccine or the nature of the event is such that the vaccine is not likely to have had any reasonable association with the observed illness/event (cause and effect relationship improbable but not impossible)
Unrelated:	The AE is completely independent of vaccine administration; and/or evidence exists that the event is definitely related to another etiology

Note:
Other factors (e.g., dechallenge, rechallenge) should be considered for each causality category when appropriate. Causality assessment is based on available information at the time of the assessment of the AE. The investigator may revise the causality assessment as additional information becomes available.

The degree of certainty with which an AE can be attributed to administration of the study vaccine will be determined by how well the event can be understood in terms of one or more of the following:

- A reaction of similar nature having previously been observed with this type of vaccine and/or formulation or naturally occurring dengue illness
- The event having often been reported in the literature for similar types of vaccines

All local injection-site reactions will be considered causally related to vaccination.

8.2.5 Adverse Event Action Taken

The investigator/designee will assess the action taken by the subject or the study staff in relation to the AE using the following classifications:

Action
1 = None
2 = Remedial therapy (more than 1 dose of medication required)
3 = Discontinued study
4 = Hospitalization
5 = Other

8.2.6 Adverse Event Outcome

The investigator/designee will assess the outcome of the AE, either at resolution or at the end of the study period, using the following classifications:

Outcome
1 = Resolved
2 = Continuing
3 = Death
4 = Unknown

8.2.7 Adverse Event Seriousness

The investigator/designee will categorize all AEs either as serious or non-serious, using the criteria defined in **Section 8.1** of this protocol.

Serious adverse events will be reported following SAE reporting guidelines outlined in **Section** Error! Reference source not found. of this protocol

8.3 Adverse Event Reporting

8.3.1 Non-Serious Adverse Events

Non-serious AEs will be followed to resolution, or until the study ends, and reported to the Sponsor as requested, to the IRB according to IRB policies, to the DSMB as required, and to the FDA at least annually in the Annual Report. AEs meeting the stopping criteria outlined in **Section 8.5** of this protocol will be reported to the Sponsor following the SAE reporting guidelines.

8.3.2 Serious Adverse Events

SAEs (whether or not they are also UPs) must be reported on the SAE/UP Report Form and sent to the Sponsor Clinical Safety Office (CSO) by fax or e-mail attachment.

Deaths and immediately life threatening SAEs must be reported within 1 business day after the site becomes aware of the event. All other SAEs must be reported within 3 business days of site awareness.

Regulatory Compliance and Human Subjects Protection Branch (RCHSPB) CSO

RCHSPB CSO
5705 Industry Lane
Frederick, MD 21704

Phone 301-846-5301
Fax 301-846-6224
E-mail: rchspsafety@mail.nih.gov

DSMB
All SAEs will be reported by telephone (followed by written report), email, or fax within 1 working day of notification of the SAE occurrence to:

- The DSMB Executive Secretary: Phone: 301-846-5301, Fax: 301-846-6224, E-mail: niaiddsmbia@mail.nih.gov

JHU Biosafety
All SAEs will be reported by telephone (followed by written report), email, or fax within 1 working day of notification of the SAE occurrence to:

- The JHU Institutional Biosafety Committee: Phone: 410-955-5918, Fax 410-955-5929

IRB Reporting
All SAEs will be reported to Western Institutional Review Board (WIRB) and UVM IRB as per guidelines, respectively for JHU and UVM as below:

- **WIRB Guidelines:**

WIRB Phone: 800-562-4789, Fax: 360-252-2498
www.wirb.com for updated reporting guidelines

- **UVM IRB Guidelines:**
  - Local, as used in the reporting guidelines below, is defined as any site under the jurisdiction of the UVM IRB.
  - Investigators are required to report AEs that fit the following criteria:
    - Report timelines are based upon of the time the investigator or site personnel becomes aware of them:
    - A local death report *within 48 hours* (Local death is defined as the death of a subject enrolled at sites under the jurisdiction of the UVM IRB, see below)
    - All other local AEs (Local AE is defined as those adverse events occurring in subjects enrolled at sites under the jurisdiction of the UVM IRB, see definition below) should be reported promptly and not to exceed 7 days
  - Local AEs (all adverse events occurring in subjects enrolled at sites under the jurisdiction of the UVM IRB, see definition below) (whether or not they are serious), that are unexpected AND possibly, probably, or definitely related to study participation:
    - LOCAL ADVERSE EVENT: is a negative side effect resulting from the study intervention that occurred to a subject enrolled at UVM, FAHC, or other research site under the jurisdiction of the UVM IRB.
    - UNEXPECTED: An event does not meet the criteria of unexpected if it is 1) included in the current protocol, drug/device brochure or the informed consent or 2) due to the subject's underlying disease or predisposing risk factors.
    - RELATED: An AE is considered to be related if there is a reasonable possibility that the event may have been caused by the protocol or study interventions. A related event has a strong temporal relationship to the drug, device, or intervention, and an alternative cause is unlikely. If it cannot be determined whether an event is related, it should be reported as "possibly related."

- At UVM, all SAEs will be reported to the University of Vermont IRB (UVM IRB) according to UVM IRB guidelines.
- UVM IRB Phone: 802-656-5040, Fax: 802-656-5041

8.3.3 Unanticipated Problems

Non-Serious AEs that are UPs must also be reported on the SAE/UP Report Form and sent to the CSO by fax or e-mail attachment no later than 7 calendar days of site awareness of the event. The UPs that are not AEs are not reported to the Sponsor CSO.

8.3.4 Pregnancy

Pregnancy itself is not an AE. However, complications of pregnancies are AEs and may be SAEs. Pertinent obstetrical information for all pregnancies will be reported to the CSO via fax or e-mail within 3 business days from site awareness of the pregnancy.

Pregnancy outcome data (e.g., delivery outcome, spontaneous, or elective termination of the pregnancy) will be reported to the CSO within 3 business days of the site's awareness of the outcome in an email.

8.4 Sponsor's Reporting Responsibilities

Serious and unexpected, suspected adverse reactions (SUSARs) as defined in 21 CFR 312.32 and determined by the IND Sponsor will be reported to FDA and all participating Investigators as IND Safety Reports.

The IND Sponsor will also submit an IND Annual Report of the progress of the investigation to the FDA on an annual basis as defined in 21 CFR 312.33.

AEs that are also UPs will be summarized by the IND Sponsor and distributed to investigators.

8.5 Stopping Criteria

If a dose of vaccine is considered unacceptably reactogenic, as defined below, additional vaccinations will be suspended until the DSMB and study Sponsor (RCHSPB) have reviewed the data and recommend that enrollment be continued.

The following criteria will be used to define unacceptable reactogenicity of the TetraVax-DV vaccine:

1. One or more subjects experience an SAE (as defined in **Section 8.1.2** in this protocol) that is determined to be possibly, probably, or definitely related to the vaccine (as defined in **Section 8.2.4** in this protocol), **OR**
2. One or more subjects experience anaphylaxis that is possibly, probably or definitely related to the vaccine, **OR**
3. One or more subjects experience an objective physical finding of severity Grade 3 that is definitely, probably, or possibly related to vaccine (with the exception of

Grade 3 erythema at the injection site), as defined in **Section 8.2.3.** in this protocol, **OR**

4. One or more subjects experience a Grade 3 laboratory abnormality that is possibly, probably, or definitely related to vaccine. **OR**
5. One or more subjects experience an ANC $\geq$500/mm^3 but <750/mm^3 for >5 days duration[1] **OR**
6. One or more subjects experience an ANC <500/mm^3 for any duration[1] **OR**
7. One or more subjects in a dose cohort experience a vaccine-associated dengue-like syndrome, defined as infection[2] associated with fever **and 2 or more** of the following symptoms:
   a. Grade 2 or greater headache lasting ≥12 hours
   b. Grade 2 or greater photophobia lasting ≥12 hours
   c. Grade 2 or greater generalized myalgia lasting ≥12 hours

[1] These stopping rules resulted from previous discussions between NIAID and the FDA on Phase 1 clinical trials of similar investigational live attenuated dengue virus vaccines (reference BB-INDs 8463, 13730, and 13886).
[2] Infection is defined as recovery of vaccine virus from the blood or serum of a subject and/or seropositivity or seroconversion to any dengue virus.

Study enrollment and vaccine administration will resume if the Sponsor review (and DSMB review recommendations) of the AEs that caused the halt results in a recommendation by the Sponsor to resume the vaccinations. Safety data reports and changes in study status will be submitted to the applicable IRB promptly, in accordance with institutional policy. This constitutes a minimum criterion, and the decision to stop the trial may be made on the basis of any other criteria which, in the judgment of the investigators, FDA, IRB or Sponsor, indicate a potentially serious safety concern.

8.6 Safety Oversight

8.6.1 Transfer of Regulatory Obligations (TORO) and Safety Review and Communications Plan (SRCP)

A Sponsor Medical Monitor (SMM), Dr. Barry Eagel representing the IND Sponsor (RCHSPB), has been appointed for oversight of safety in this clinical study. In addition, the IND Sponsor and the PI have signed a formal TORO/SRCP which documents that the PI has accepted the responsibility for routine data analysis as per §21CFR312.32c(1)(i)(C). The PI will submit a copy of the routine safety data analysis to the SMM in the Sponsor Clinical Safety Office (CSO) within 5 days following the review. If the PI identifies any safety concerns during the routine reviews, the PI will immediately notify the Sponsor CSO.

8.6.2 Data and Safety Monitoring Board (DSMB)

The NIAID Intramural DSMB will review the study prior to initiation and twice a year thereafter. The Board may convene additional reviews as necessary. The Board will review the study data to evaluate the safety, efficacy, study progress, and conduct of the study. All serious adverse events that are possibly, probably or definitely related to the study agent, all unanticipated problems, and all IND Safety Reports will be reported by the PI to the DSMB at the same time they are submitted to the IRB or IND Sponsor. The PI will notify the DSMB of any cases of intentional or unintentional unblinding as soon as possible. The PI will notify the Board at the time stopping criteria are met and obtain a recommendation concerning continuation, modification, or termination of the study. The PI will submit the written DSMB summary reports with recommendations to the IRB(s).

9 Data Collection and Monitoring

9.1 Source Documentation and Data Collection

Complete source documentation (laboratory test reports, hospital or medical records, progress notes, observations, etc.) is required for every study subject for the duration of the study. The subject's study record must record his/her participation in the clinical trial and, after unblinding, the randomization treatment received (with doses and frequency) or other concomitant medications or interventions administered, as well as any adverse reactions experienced during the trial.

Data from source documentation for subjects enrolled in the study will be entered into the Clinical Research Information Management System of the NIAID (CRIMSON) Data System. The data entry is to be completed on an ongoing basis during the study. Data entry into CRIMSON shall be performed by authorized individuals and each individual entering data into CRIMSON shall have a unique user ID and password. Corrections to the data system shall be tracked electronically (password protected) with time, date, individual making the correction, and what was changed.

Corrections to the source document must be made by striking through the incorrect entry with a single line (taking care not to obliterate or render the original entry illegible) and entering the correct information adjacent to the incorrect entry. Corrections must be initialed and dated by the person making the correction whenever possible. Source documentation should support the data collected in CRIMSON**,** and must be signed and dated by the person recording and/or reviewing the data.

The investigator is responsible for the accuracy, completeness and timeliness of the data reported to the Sponsor in the CRIMSON Data System. All data entered into CRIMSON should be reviewed by the investigator/designee and signed as required with written or electronic signature, as appropriate. Data reported in CRIMSON should be consistent with source documents or the discrepancies should be explained. Source documentation will be made available for review or audit by the Sponsor or designee and any applicable Federal authorities.

9.2 Study Documentation

Study-related documentation will be completed as required by the IRBs, the Sponsor, and regulatory authorities. Continuing review documentation will be submitted by the investigator to the IRBs by the anniversary date of initial review as specified by each IRB. An annual report will be submitted by the Sponsor to the FDA according to regulations. These reports will provide a brief description of the progress of the investigation as outlined in 21 *Code of Federal Regulations* 312.33, and will include any revisions of the protocol if not previously submitted.

The PI will maintain adequate records to account for the disposition of the investigational product, including dates of receipt and quantity, current inventory, and dispensation to subjects. If the study is terminated, suspended, or completed, the PI will return all unused supplies of the investigational product to a NIAID approved repository or destroy according to the Sponsor's recommendation.

9.3 Retention of Specimens

All specimens collected as part of this trial will be stored for future research as part of our approved biosample repository for vaccine research. These samples may be used to learn more about flavivirus infection and other diseases. These samples will not be sold or used to make commercial products. All samples stored in the repository will be labeled with the study ID numbers of the subjects that, by themselves, cannot identify study subjects, but are linkable to other research databases (e.g., from questionnaires, clinical assessments, logbooks, etc.) generated by the main study. The repository database will contain only the study subject ID numbers. A master log linking the study subject ID numbers to the names of the subjects will be maintained in a password protected database system with limited access to authorized research team members.

9.4 Retention of Records

The PI is responsible for retaining all essential documents listed in the International Conference on Harmonization (ICH) Good Clinical Practice (GCP) Guideline. Trial-related documents will be maintained by the investigator in a secure storage facility for a period of 2 years after final marketing approval of the vaccine, or if 3 years have elapsed since the formal discontinuation of clinical development of the product. These records are also to be maintained in compliance with IRB, state, and federal medical records retention requirements, whichever is longest. The Sponsor is required to inform the investigator as to when such documents need no longer be retained. Storage of all trial-related documents will be such that confidentiality will be strictly maintained to the extent provided by federal, state, and local law.

It is the PI's responsibility to retain copies of source documents until receipt of written notification to the contrary from the RCHSPB of the NIAID. No study documents should be destroyed without prior written agreement between RCHSPB/NIAID and the PI. Should the PI wish to assign the study records to another party and/or move to another location, the PI must provide written notification of such intent to RCHSPB/NIAID with the name of the person who will accept responsibility for the transferred records and/or their new location.

NIAID must be notified in writing and written permission must be received by the site prior to destruction or relocation of research records.

9.5 Protocol Compliance

The PI will conduct the trial in compliance with the protocol agreed to by the Sponsor. The investigator will not implement any deviation from, or changes of, the protocol without agreement, prior review and documented approval by the Sponsor and the IRB that granted original approval for the study. The DSMB will be made aware of all protocol revisions (other than administrative) and will review any changes to the protocol that involve DSMB oversight or involve changes to the data and safety monitoring plan of the study.

However, the investigator may implement a deviation from, or change in, the protocol to eliminate an immediate hazard(s) to subjects without prior IRB or Sponsor approval or when the change(s) involves only logistical or administrative aspects of the trial (i.e., change of telephone number[s]). In the event of a medical emergency, the PI shall perform any medical procedures that are deemed medically appropriate.

As soon as possible, the implemented deviation or change, the reasons for it, and, if appropriate, the proposed protocol amendment(s) should be submitted to the Sponsor, IRB, DSMB, and to the regulatory authorities.

9.6 Clinical Investigator’s Brochure

Investigators will receive the current version of the Clinical Investigator’s Brochure, which comprehensively describes all the available preclinical experience with the experimental vaccine. If relevant new information becomes available during the course of the trial, the investigators will receive a revised brochure, or an amendment to the current version.

9.7 Study Monitoring

As per ICH-GCP 5.18 and FDA 21 CFR 312.50 clinical protocols are required to be adequately monitored by the Sponsor. This study monitoring will be conducted according to the “NIAID Intramural Clinical Monitoring Guidelines.” The Sponsor will monitor all aspects of the study, with respect to current GCP, for compliance with applicable government regulations. Prior to the start of the study, the investigator will be informed of the frequency of monitoring visits and will be given reasonable notifications prior to each visit. The objectives of a monitoring visit will be to verify the prompt and accurate recording of all monitored data points, and prompt reporting of SAEs; to check the availability of signed Informed Consent forms and documentation of the informed consent process for each monitored subject; to compare CRIMSON reports and line listings with source data for completeness and accuracy; and to help ensure investigators are in compliance with the protocol. The monitors also will inspect the clinical site regulatory files to ensure that regulatory requirements (Office for Human Research Protections-OHRP, FDA) and applicable guidelines (ICH-GCP) are being followed. During the monitoring visit, the investigator (and/or designee) and other study personnel should be available to discuss the study. Study documents must be available for review throughout the course of the study. The Sponsor will retain original copies of the Form FDA 1572 and copies of other study documents as deemed necessary.

10 Statistical Considerations

10.1 General Design

The goal of this Phase 1 vaccine trial is to determine the safety and immunogenicity of TetraVax-DV, a live attenuated tetravalent dengue vaccine candidate in healthy human flavivirus experienced subjects. The purpose of this Phase 1 trial is to evaluate the reactogenicity, immune response to the 4 DENV serotypes, and persistence of antibody following subcutaneous administration of a single dose of Tetra-Vax-DV vaccine.

10.2 Statistical Methods

This study, like other Phase 1 studies, is exploratory rather than confirmatory; its purpose is to estimate event frequencies and patterns of immune responses rather than to test formal statistical hypotheses. Descriptive approaches will be used to meet the protocol objectives as stated in **Section 3** of this protocol, as outlined below. Results will be presented in tabular format, as well as graphically where appropriate.

Primary Objective 1: Determine the safety of TetraVax-DV admixtures TV003 & TV005in flavivirus-experienced healthy adults as assessed by:

- Summarizing the frequency of immediate, systemic, and local AEs following vaccination. AEs will be displayed in tabular format, with line listings of individual clinical and laboratory AEs classified as immediate, systemic, and local events. AEs will be summarized by severity and relationship to vaccine by individuals and each group.

Primary Objective 2: To determine the immunogenicity of TetraVax-DV admixtures TV003 & TV005, as assessed by:

- Determination of the seroconversion rate by $PRNT_{50}$ to DENV-1, DENV-2, DENV-3, and DENV-4 viruses for each flavivirus experienced subject by comparing PRNT at Study Day 0 with PRNT on Study Days 14, 21, 28, 56, 90, and 180 post-each vaccination. Monovalent, bivalent, trivalent, and tetravalent seropositivity and seroconversion rates will be determined at these time points.

Secondary Objective 1: Assess the frequency, quantity, and duration of viremia of each monovalent component of the vaccine after a single vaccine dose in persons with laboratory evidence of pre-existing flavivirus antibody titer and/or documented history of receipt of a flavivirus vaccine. The mean peak viremia, mean day of onset of viremia, and mean duration of viremia of each monovalent component within each dose cohort will be calculated.

Secondary Objective 2: Determine the number of vaccinees infected with DENV-1, DENV-2, DENV-3, and DENV-4. Infection is defined as recovery of vaccine virus from the blood or serum of a subject with preexisting seropositivity that undergoes seroconversion to DEN virus. Seropositivity will be defined as a:

- $PRNT_{50}$ ≥1:10 by Study Day 90 post-vaccination

Seroconversion will be defined as:

- ≥4-fold rise in DENV-1, DENV-2, DENV-3, or DENV-4 neutralizing antibody titers by Day 28 compared with the pre-vaccination antibody titer

Secondary Objective 3: Assess the duration of the antibody response in recipients of the tetravalent vaccine. The $PRNT_{50}$ to DENV-1, DENV-2, DENV-3, and DENV-4 will be determined for all specimens collected at Study Day 180 post vaccination.

Exploratory Objective 1: Exploratory Objective 1: To evaluate the cellular immune response to secondary infection with the TetraVax-DV vaccine.

Exploratory Objective 2: To evaluate B and T cell memory responses following secondary infections with TetraVax-DV vaccine.

Exploratory Objective 3: To evaluate the innate immune response in flavivirus-experienced subjects following vaccination with TV003 orTV005.

10.3 Safety

The primary safety endpoint is the frequency of vaccine-related AEs, as classified by both severity and seriousness, through active and passive surveillance. Separate assessments of systemic and local reactions will be performed.

10.4 Immunogenicity

Dengue virus neutralizing antibody titers will be measured on Study Days 0, 14, 28, 56, 90, 150, and 180. Seropositivity to each serotype will be defined as a $PRNT_{50}$ of ≥1:10 by Study Day 56 compared with Study Day 0 (Day of Vaccination). Seroconversion to each serotype

will be defined as a ≥4-fold in serum neutralizing antibody titer by Study Day 28 compared with Study Day 0 (Day of Vaccination).

11 Protection of Human Subjects

11.1 Institutional Review Boards

The PI will be responsible for obtaining IRB approval for the study. Before the start of the study, the appropriate documents (including, but not limited to, the Protocol, Investigator's Brochure, Informed Consent Form, information sheets, and advertisements) will be submitted to the IRB for approval. A copy of the study approval (including approval of the informed consent form) is to be maintained in the investigator study document binder and a copy will be supplied to the Sponsor. During the study, the investigator is responsible for providing the IRB with all documents subject to review (e.g., Protocol Amendments, informed consent form updates, advertisements, and any written information that may be provided to the subject). Annual reports on the progress of the study will be made to the IRB by the investigator in accordance with IRB guidelines and government regulations.

11.2 Informed Consent

In obtaining and documenting informed consent, the investigator and study staff must comply with the applicable regulatory requirements, GCP guidelines, and ethical principles. The written informed consent form must be approved by the appropriate IRB prior to its use. The subject may withdraw consent at any time during the course of the trial. A copy of the informed consent document will be given to the subject for his or her records. The rights and welfare of the subjects will be protected by emphasizing to them that the quality of their medical care will not be adversely affected if they decline to participate in this study.

11.3 Risks

Risks to the subjects are associated with venipuncture and immunization. These risks are outlined below. Female subjects will be cautioned of the unknown risk of study vaccines to the fetus and will be advised to use effective birth control methods for the duration of the study.

11.3.1 Venipuncture

The total amount of blood to be drawn throughout the 12-month duration of the study is approximately 870 mL.

Risks occasionally associated with venipuncture include excessive bleeding, pain, bruising, or hematoma at the site of venipuncture, lightheadedness, and syncope (rarely). Infection may occur rarely.

11.3.2 Immunization

Possible local vaccine reactions include pain, swelling, or erythema for 2 to 3 days, lymphadenopathy, or pruritus at the injection site. Systemic reactions such as macular papular rash and transient neutropenia have been observed in some subjects infected with other recombinant dengue vaccine candidates. Other potential systemic reactions that may occur include symptoms of dengue such as fever, headache, eye pain, photophobia,

generalized myalgias, arthralgias, elevated ALT, neutropenia, elevated PTT, or decreased platelet count. Immediate hypersensitivity reactions including urticaria, anaphylaxis, or other IgE-mediated responses are possible, as with any vaccine. Subjects who receive the TetraVax-DV vaccine may be at increased risk of more severe disease (DHF/DSS) if they respond poorly to 1 or more of the components of the vaccine and become infected with a DEN virus serotype in the future.

Subjects may be asked to defer routine immunization (such as influenza) until after 42 days following either vaccination. This may increase the risk that the subject will be infected with an influenza virus during this period. As with any investigational vaccine, there is a theoretical possibility of risks about which we have no present knowledge. Subjects will be informed of any such risks should further information become available.

11.4 Benefits

Subjects will not receive any direct benefit from participation in this study. They will receive a physical examination and laboratory screening for HIV infection, Hepatitis B infection, and Hepatitis C infection. They may potentially develop antibodies against 1 or more dengue viruses. It is hoped that information gained in this study will contribute to the development of a safe and effective tetravalent vaccine for the prevention of DEN infection.

11.5 Compensation

Subjects will be compensated up to $75 for screening, up to $100 for each vaccination, and up to $75 for each completed scheduled follow-up visit. They will also receive up to a $450 bonus if all study visits are completed on time. Subjects will be compensated for the screening only if they are ***enrolled*** in the study, which is defined as receipt of vaccine or placebo. Subjects will only be compensated for the visits that they complete. Alternates may receive compensation for screening and vaccination day.

Subjects enrolled in the study will receive a maximum total compensation of up to $2375.

11.6 Confidentiality

All study-related information will be stored securely at the study site. All subject information will be stored in locked file cabinets in areas with access limited to study staff. All laboratory specimens, reports, study data collection, and process and administrative forms will be identified by coded number only to maintain subject confidentiality. Computer entry will be done using a study ID number for each subject and all local databases will be secured with password-protected access systems. Forms, lists, logbooks, appointments books, and any other listings that link subject study ID numbers to other identifying information will be stored in a separate, locked file in an area with limited access. A subject's study information will not be released without the written permission of the subject, except as necessary for monitoring by the Sponsor and/or its contractors and the FDA.

11.7 Biohazard Containment

As the transmission of HIV and other blood-borne pathogens can occur through contact with contaminated needles, blood, and blood products, appropriate blood and secretion precautions will be employed by all personnel during the drawing of blood and shipping and handling of

all specimens for this study, as currently recommended by the Centers for Disease Control and Prevention and the NIH.

All infectious specimens will be transported using packaging mandated in the Code of Federal Regulations, 42 CFR Part 72. Please also refer to individual carrier guidelines (e.g., Federal Express, Airborne Express) for specific instructions.

12 Publication Policy

The International Committee of Medical Journal Editors (ICMJE) member journals have adopted a trials-registration policy as a condition for publication. This policy requires that all clinical trials be registered prior to enrollment of any subject in a public trials registry such as ClinicalTrials.gov, which is sponsored by the National Library of Medicine. The ICMJE defines a clinical trial as any research project that prospectively assigns human subjects to intervention or comparison groups to study the cause-and-effect relationship between a medical intervention and a health outcome. Studies designed for other purposes, such as to study pharmacokinetics or major toxicity, would be exempt from this policy.

13 References

1. **Bellanti, J. A., R. T. A. Bourke, E. C. Rosenzweig, and O. R. Eylar.** 1966. Report of dengue vaccine trial in the Caribbean, 1963: a collaborative study. Bull World Health Organ **35:**93.

2. **Bhamarapravati, N., and Y. Sutee.** 2000. Live attenuated tetravalent dengue vaccine. Vaccine **18:**44-47.

3. **Blaney, J. E., Jr., A. P. Durbin, B. R. Murphy, and S. S. Whitehead.** 2006. Development of a live attenuated dengue virus vaccine using reverse genetics. Viral Immunol **19:**10-32.

4. **Blaney, J. E., Jr., C. T. Hanson, C. Y. Firestone, K. A. Hanley, B. R. Murphy, and S. S. Whitehead.** 2004. Genetically modified, live attenuated dengue virus type 3 vaccine candidates. Am J Trop Med Hyg **71:**811-821.

5. **Blaney, J. E., Jr., N. S. Sathe, L. Goddard, C. T. Hanson, T. A. Romero, K. A. Hanley, B. R. Murphy, and S. S. Whitehead.** 2008. Dengue virus type 3 vaccine candidates generated by introduction of deletions in the 3' untranslated region (3'-UTR) or by exchange of the DENV-3 3'-UTR with that of DENV-4. Vaccine **26:**817-828.

6. **Burke, D. S., A. Nisalak, D. E. Johnson, and R. M. Scott.** 1988. A prospective study of dengue infections in Bangkok. Am J Trop Med Hyg **38:**172-180.

7. **Couvelard, A., P. Marianneau, C. Bedel, M. T. Drouet, F. Vachon, D. Henin, and V. Deubel.** 1999. Report of a fatal case of dengue infection with hepatitis: demonstration of dengue antigens in hepatocytes and liver apoptosis. Hum Pathol **30:**1106-1110.

8. **Durbin, A. P., R. A. Karron, W. Sun, D. W. Vaughn, M. J. Reynolds, J. R. Perreault, B. Thumar, R. Men, C. J. Lai, W. R. Elkins, R. M. Chanock, B. R. Murphy, and S. S. Whitehead.** 2001. Attenuation and immunogenicity in humans of a live dengue virus type-4 vaccine candidate with a 30 nucleotide deletion in its 3'-untranslated region. Am J Trop Med Hyg **65:**405-413.

9. **Durbin, A. P., J. McArthur, J. A. Marron, J. E. Blaney, Jr., B. Thumar, K. Wanionek, B. R. Murphy, and S. S. Whitehead.** 2006. The live attenuated dengue serotype 1 vaccine rDEN1Delta30 is safe and highly immunogenic in healthy adult volunteers. Hum Vaccin **2:**167-173.

10. **Durbin, A. P., J. H. McArthur, J. A. Marron, J. E. Blaney, B. Thumar, K. Wanionek, B. R. Murphy, and S. S. Whitehead.** 2006. rDEN2/4Delta30(ME), A Live Attenuated Chimeric Dengue Serotype 2 Vaccine Is Safe and Highly Immunogenic in Healthy Dengue-Naive Adults. Hum Vaccin **2:**255-260.

11. **Durbin, A. P., A. Schmidt, D. Elwood, K. A. Wanionek, J. Lovchik, B. Thumar, B. R. Murphy, and S. S. Whitehead.** 2011. Heterotypic dengue infection with live attenuated monotypic dengue virus vaccines: implications for vaccination of populations in areas where dengue is endemic. J Infect Dis **203:**327-334.

12. **Durbin, A. P., S. S. Whitehead, J. McArthur, J. R. Perreault, J. E. Blaney, Jr., B. Thumar, B. R. Murphy, and R. A. Karron.** 2005. rDEN4 Delta 30, a Live Attenuated Dengue Virus Type 4 Vaccine Candidate, Is Safe, Immunogenic, and Highly Infectious in Healthy Adult Volunteers. J Infect Dis **191:**710-718.

13. **Durbin, A. P., S. S. Whitehead, D. Shaffer, D. Elwood, K. Wanionek, J. E. Blaney, Jr., B. R. Murphy, and A. C. Schmidt.** 2011. A single dose of the DENV-1 candidate vaccine rDEN1Δ30 is strongly immunogenic and induces resistance to a second dose in a randomized trial. PLoS Negl Trop Dis **5**.

14. **Edelman, R., C. O. Tacket, S. S. Wasserman, D. W. Vaughn, K. H. Eckels, D. R. Dubois, P. L. Summers, and C. H. Hoke.** 1994. A live attenuated dengue-1 vaccine candidate (45AZ5) passaged in primary dog kidney cell culture is attenuated and immunogenic for humans. J Infect Dis **170:**1448-1455.

15. **Edelman, R., S. S. Wasserman, S. A. Bodison, R. J. Putnak, K. H. Eckels, D. Tang, N. Kanesa-Thasan, D. W. Vaughn, B. L. Innis, and W. Sun.** 2003. Phase I trial of 16 formulations of a tetravalent live-attenuated dengue vaccine. Am J Trop Med Hyg **69:**48-60.

16. **Hanley, K. A., L. R. Manlucu, G. G. Manipon, C. T. Hanson, S. S. Whitehead, B. R. Murphy, and J. E. Blaney, Jr.** 2004. Introduction of mutations into the non-structural genes or 3' untranslated region of an attenuated dengue virus type 4 vaccine candidate further decreases replication in rhesus monkeys while retaining protective immunity. Vaccine **22:**3440-3448.

17. **Kalayanarooj, S., D. W. Vaughn, S. Nimmannitya, S. Green, S. Suntayakorn, N. Kunentrasai, W. Viramitrachai, S. Ratanachu-eke, S. Kiatpolpoj, B. L. Innis, A. L. Rothman, A. Nisalak, and F. A. Ennis.** 1997. Early clinical and laboratory indicators of acute dengue illness. J Infect Dis **176:**313-321.

18. **Kanesa-thasan, N., W. Sun, G. Kim-Ahn, S. Van Albert, J. R. Putnak, A. King, B. Raengsakulsrach, H. Christ-Schmidt, K. Gilson, J. M. Zahradnik, D. W. Vaughn, B. L. Innis, J. F. Saluzzo, and C. H. Hoke, Jr.** 2001. Safety and immunogenicity of attenuated dengue virus vaccines (Aventis Pasteur) in human volunteers. Vaccine **19:**3179-3188.

19. **Kuo, C. H., D. I. Tai, C. S. Chang Chien, C. K. Lan, S. S. Chiou, and Y. F. Liaw.** 1992. Liver biochemical tests and dengue fever. Am J Trop Med Hyg **47:**265-270.

20. **Marianneau, P., A. M. Steffan, C. Royer, M. T. Drouet, D. Jaeck, A. Kirn, and V. Deubel.** 1999. Infection of primary cultures of human Kupffer cells by Dengue virus: no viral progeny synthesis, but cytokine production is evident. J Virol **73:**5201-5206.

21. **Monath, T., and F. Heinz.** 1996. Flaviviruses, p. 961-1034. *In* B. N. Fields, D. M. Knipe, P. M. Howley, R. M. Chanock, J. L. Melnick, T. P. Monath, B. Roizman, and S. E. Straus (ed.), Fields Virology, third ed, vol. 1. Lipincott-Raven, Philadelphia.

22. **Murgue, B., C. Roche, E. Chungue, and X. Deparis.** 2000. Prospective study of the duration and magnitude of viraemia in children hospitalised during the 1996-1997 dengue-2 outbreak in French Polynesia. J Med Virol **60:**432-438.

23. **Sabchareon, A., J. Lang, P. Chanthavanich, S. Yoksan, R. Forrat, P. Attanath, C. Sirivichayakul, K. Pengsaa, C. Pojjaroen-Anant, W. Chokejindachai, A. Jagsudee, J. F. Saluzzo, and N. Bhamarapravati.** 2002. Safety and immunogenicity of tetravalent live-attenuated dengue vaccines in Thai adult volunteers: role of serotype concentration, ratio, and multiple doses. Am J Trop Med Hyg **66:**264-272.

24. **Sabin, A.** 1955. Recent advances in our knowledge of Dengue and Sandfly Fever. Amer J Trop Med Hyg **4:**198-207.

25. **Sabin, A. B., and R. W. Schlesinger.** 1945. Production of immunity to dengue with virus modified by propagation in mice. Science **101:**640-642.

26. **Schlesinger, R. W., I. Gordon, J. W. Frankel, J. W. Winter, P. R. Patterson, and W. R. Dorrance.** 1956. Clinical and serologic response of man to immunization with attnuated dengue and yellow fever viruses. J Immunol **77:**352-364.

27. **Sohn, Y. M., J. B. Tandan, S. Yoksan, M. Ji, and H. Ohrr.** 2008. A 5-year follow-up of antibody response in children vaccinated with single dose of live attenuated SA14-14-2 Japanese encephalitis vaccine: immunogenicity and anamnestic responses. Vaccine **26:**1638-1643.

28. **Sun, W., R. Edelman, N. Kanesa-Thasan, K. H. Eckels, J. R. Putnak, A. D. King, H. S. Houng, D. Tang, J. M. Scherer, C. H. Hoke, Jr., and B. L. Innis.** 2003. Vaccination of human volunteers with monovalent and tetravalent live-attenuated dengue vaccine candidates. Am J Trop Med Hyg **69:**24-31.

29. **Troyer, J. M., K. A. Hanley, S. S. Whitehead, D. Strickman, R. A. Karron, A. P. Durbin, and B. R. Murphy.** 2001. A live attenuated recombinant dengue-4 virus vaccine candidate with restricted capacity for dissemination in mosquitoes and lack of transmission from vaccinees to mosquitoes. Am J Trop Med Hyg **65:**414-419.

30. **Vaughn, D. W.** 2000. Presented at the International Conference on Dengue and DHF, Chiang Mai, Thailand, 20-24 November.

31. **Whitehead, S. S., B. Falgout, K. A. Hanley, J. E. Blaney Jr, Jr., L. Markoff, and B. R. Murphy.** 2003. A Live, Attenuated Dengue Virus Type 1 Vaccine Candidate with a 30-Nucleotide Deletion in the 3' Untranslated Region Is Highly Attenuated and Immunogenic in Monkeys. J Virol **77:**1653-1657.

32. **Whitehead, S. S., K. A. Hanley, J. E. Blaney, Jr., L. E. Gilmore, W. R. Elkins, and B. R. Murphy.** 2003. Substitution of the structural genes of dengue virus type 4 with those of type 2 results in chimeric vaccine candidates which are attenuated for mosquitoes, mice, and rhesus monkeys. Vaccine **21:**4307-4316.

33. **WHO.** 1997. Dengue haemorrhagic fever: diagnosis, treatment, prevention and control., 2nd ed. WHO, Geneva.

34. **Wisseman, C. L., Jr., B. H. Sweet, E. C. Rosenzweig, and O. R. Eylar.** 1963. Attenuated Living Type 1 Dengue Vaccines. Am J Trop Med Hyg **12:**620-623.

35. **Wu, S. J., G. Grouard-Vogel, W. Sun, J. R. Mascola, E. Brachtel, R. Putvatana, M. K. Louder, L. Filgueira, M. A. Marovich, H. K. Wong, A. Blauvelt, G. S. Murphy, M. L. Robb, B. L. Innes, D. L. Birx, C. G. Hayes, and S. S. Frankel.** 2000. Human skin Langerhans cells are targets of dengue virus infection [see comments]. Nat Med **6:**816-820.

Appendix 1: Adverse Event Grading Table

**Event**	**Grade I – Mild**	**Grade II – Moderate**	**Grade III - Severe**
**General Severity Grading**	**Event that is easily tolerated, may require 1 dose of medication/treatment**	**Event that interferes with daily activity or requires more than 1 dose of medication/treatment**	**Event that prevents daily activity and requires medical intervention**
Local Reactogenicity	Grade I – Mild	Grade II – Moderate	Grade III - Severe
Injection Site Tenderness	Tenderness that is easily tolerated	Tenderness that interferes with daily activity	Tenderness that prevents daily activity
Injection Site Pain	Pain that is easily tolerated	Pain that interferes with daily activity	Pain that prevents daily activity
Injection Site Pruritus	Pruritus that is easily tolerated	Pruritus that interferes with daily activity	Pruritus that prevents daily activity
Injection Site Induration	>0 - 20 mm	>20 - 50 mm	>50 mm
Injection Site Erythema	>0 - 20 mm	>20 - 50 mm	>50 mm
Injection Site Swelling	>0 - 20 mm	>20 - 50 mm	>50 mm
Systemic Reactogenicity	Grade I – Mild	Grade II – Moderate	Grade III - Severe
Fever (Oral)	100.4° F – 101.4°F	101.5°F – 102.4°F	≥102.5°F
Headache	Headache that is easily tolerated	Headache that interferes with daily activity or requires >1 dose of medication	Headache that prevents daily activity
Retro-Orbital Pain (ROP)	ROP that is easily tolerated	ROP that interferes with daily activity or requires >1 dose of medication	ROP that prevents daily activity
Photophobia	Photophobia that is easily tolerated	Photophobia that interferes with daily activity or requires >1 dose of medication	Photophobia that prevents daily activity
Nausea	Nausea that is easily tolerated	Nausea that interferes with daily activity or requires >1 dose of medication	Nausea that prevents daily activity
Fatigue	Fatigue that is easily tolerated	Fatigue that interferes with daily activity or requires >1 dose of medication	Fatigue that prevents daily activity

**Event**	**Grade I – Mild**	**Grade II – Moderate**	**Grade III - Severe**
Myalgia	Myalgia that is easily tolerated	Myalgia that interferes with daily activity or requires >1 dose of medication	Myalgia that prevents daily activity
Arthralgia	Arthralgia that is easily tolerated	Arthralgia that interferes with daily activity or requires >1 dose of medication	Arthralgia that prevents daily activity
Dengue Rash	Rash is present but asymptomatic	Rash is symptomatic (pruritus/pain) but does not interfere with function	Rash is symptomatic and interferes with function
Solicited Lab AEs	Grade I – Mild	Grade II – Moderate	Grade III - Severe
Hemoglobin (female)	9.5 - 10.7 gm/dL	8.0 - 9.4 gm/dL	$\leq$7.9 gm/dL
Hemoglobin (male)	11.0 - 12.5 gm/dL	9.0 – 10.9 gm/dL	$\leq$8.9 gm/dL
Neutropenia (Reduced ANC)[1]	≥750-999/mm^3	≥500-749/mm3	$\leq$500/mm^3
Leukocytosis (Increased WBCs)	11,500 - 13,000/mm^3	13,001 - 15,000/mm^3	$\geq$15,000 or <1,000/mm^3
Thrombocytopenia (Reduced Platelets)	100,000 – 120,000/mm^3	75,000 - 99,999/ mm^3	$\leq$74,999/mm^3
PT	>1.0 - 1.25 x ULN	>1.25 - 1.5 x ULN	>1.5 x ULN
PTT	>1.0 - 1.66 x ULN	>1.66 - 2.33 x ULN	>2.33 x ULN
ALT	>1.25 - 2.5 x ULN	>2.5 - 5.0 x ULN	>5.0 x ULN
Creatinine	1.5 - 1.7 mg/dL	>1.7 – 2.0 mg/dL	>2.0 mg/dL
Other Laboratory Values	Grade I – Mild	Grade II – Moderate	Grade III - Severe
Fibrinogen, Decreased	100 mg/dL to < LLN	50 - 99 mg/dL	<50 mg/dL, or associated with gross bleeding, or associated with disseminated coagulation
Fibrinogen, Increased	>ULN to 600 mg/dL	>600 mg/dL	N/A
CPK	$\geq$4 x ULN- 6 x ULN	>6 x ULN- 10 x ULN	>10 x ULN
Sodium: Hyponatremia	130 – 134 mEq/L	123 – 129 mEq/L	<122 mEq/L
Sodium: Hypernatremia	145 – 150 mEq/L	151 – 157 mEq/L	>158 mEq/L

**Event**	**Grade I – Mild**	**Grade II – Moderate**	**Grade III - Severe**
Potassium: Hypokalemia	3.1 – 3.2 mEq/L	2.9 – 3.0 mEq/L	<2.8 mEq/L
Potassium: Hyperkalemia	5.2 – 5.5 mEq/L	5.6 – 6.0 mEq/L	>6.1 mEq/L
Phosphate: Hypophosphatemia	2.0 – 2.2 mg/dL	1.5 – 1.9 mg/dL	<1.4 mg/dL
Calcium (Corrected For Albumin): Hypocalcemia	1.95 – 2.04 mmol/L	1.75 – 1.94 mmol/L	<1.74 mmol/L
Calcium (Corrected For Albumin): Hypercalcemia	2.51 – 2.88 mmol/L	2.89 – 3.13 mmol/L	>3.14 mmol/L
Magnesium: Hypomagnesemia	0.60 – 0.74 mmol/L	0.45 – 0.59 mmol/L	<0.44 mmol/L
Bilirubin (hyperbilirubinemia)	>1.0 – 1.5 x ULN	>1.5 – 2.5 x ULN	>2.5 ULN
Glucose: Hypoglycemia (Nonfasting, No Prior Diabetes)	55 – 69 mg/dL	40 – 54 mg/dL	<39 mg/dL
Glucose: Hyperglycemia (Nonfasting, No Prior Diabetes)	116 – 160 mg/dL	161 – 250 mg/dL	>251mg/dL
Triglycerides	—	400 – 750 mg/dL	>751 mg/dL
Liver Transaminase (LFTs)<br>GGT<br>AST<br>Alk Phos	 >1.25 – 2.5 x ULN<br>>1.25 – 2.5 x ULN<br>>1.25 – 2.5 x ULN	 >2.5 – 5.0 x ULN<br>>2.5 – 5.0 x ULN<br>>2.5 – 5.0 x ULN	 >5.0 x ULN<br>>5.0 x ULN<br>>5.0 x ULN
Pancreatic Amylase<br>Lipase	>1.0 – 1.5 x ULN<br>>1.0 – 1.5 x ULN	>1.5 – 2.0 x ULN<br>>1.5 – 2.0 x ULN	>2.0 x ULN<br>>2.0 x ULN
Other Cardiovascular	Grade I – Mild	Grade II – Moderate	Grade III - Severe
Cardiac Arrhythmia	Asymptomatic; transiendysrhythia, no therapy required	Recurrent/persistent dysrhythmia; symptomatic therapy required	unstable dysrhythmia, hospitalization and therapy required

**Event**	**Grade I – Mild**	**Grade II – Moderate**	**Grade III - Severe**
Hypertension	Transient, increase >20 mm Hg diastolic BP; no therapy required	Recurrent; chronic increase >20 mm Hg diastolic BP; therapy req.	acute therapy required outpatient or hospitalization possible
Hypotension	Transient orthostatic hypotension with heart rate increased by 20 beats/min or decreased by <10 mm Hg systolic BP, no therapy required	Symptoms or BP decreased by <20 mm Hg systolic, correctable with oral fluid therapy	Mean arterial pressure <60 mm Hg, IV fluids required, or hospitalization
Pericarditis	Mild/moderate asymptomatic effusion, no therapy	Symptomatic effusion, pain, EKG changes	Tamponade or pericardiocentesis or surgery required
Hemorrhage, Blood Loss	Mildly symptomatic, no therapy required	Gross blood loss or 1 – 2 units transfused	Massive blood loss or >2 units transfused
Other Gastrointestinal	Grade I – Mild	Grade II – Moderate	Grade III - Severe
Vomiting	Mild or transient; 2 – 3 episodes per day or mild vomiting lasting <1 week	Moderate or persistent; 4 – 5 episodes per day; or vomiting lasting ≥1 week, therapy required	Severe vomiting of all food/fluids in 24 hours or orthostatic hypotension or IV therapy required
Diarrhea	Mild or transient; 3 - 4 loose stools per day or mild diarrhea lasting less than 1 week	Moderate or persistent; 5 - 10 loose stools per day or diarrhea lasting ≥1 week, therapy required	>10 loose stools/day bloody diarrhea; or orthostatic hypotension or electrolyte imbalance, >2 L IV fluid required
Oral Discomfort/Dysphagia	Difficulty swallowing but able to eat and drink	Unable to swallow solids	Unable to drink fluids; IV fluids required
Constipation	Constipation less than 78 hours and requires medication for relief	Moderate abdominal pain 78 hours with impaction, requiring therapy	Requiring disimpaction or hospital treatment
Other Respiratory	Grade I – Mild	Grade II – Moderate	Grade III - Severe

Event	Grade I – Mild	Grade II – Moderate	Grade III - Severe
Bronchospasm Acute	Transient; no therapy; FEV1 or peak flow reduced to 70- <80%	Therapy required; normalizes with bronchodilator; FEV1 or peak flow 50 - 69%	No normalization with bronchodilator; FEV1 or peak flow 25 – 49%, retractions
Dyspnea	Dyspnea on exertion	Dyspnea with normal activity	Dyspnea at rest
Other Neurologic	Grade I – Mild	Grade II – Moderate	Grade III - Severe
Nuchal Rigidity	--	--	Presence of Nuchal rigidity
Neuropsychological	Mild confusion or cognitive impairment	Moderate confusion or cognitive impairment	Severe confusion or cognitive impairment
Neurocerebellar	Slight incoordination or dysdiadochokinesia	Intention tremor, dysmetria, slurred speech, or nystagmus	Ataxia requiring assistance to walk or arm incoordination interfering with ADLs
Neuromotor	Mild weakness in muscle of feet, but able to walk; and/or mild increase in reflexes	Moderate weakness in feet or legs, e.g. unable to perform deep knee bend, mild weakness in hands, loss of previously present reflex or development of hyperreflexia.	Marked distal weakness
Neurosensory	Mild impairment (decreased sensation in focal area or symmetrical distribution)	Moderate symmetrical impairment, mild impairment that is not symmetrical	Severe impairment (decreased or loss of sensation to knees or wrists) or loss of sensation of at least moderate degree in multiple areas or functions
Paresthesia (Burning, Tingling, etc.)	Mild discomfort; no therapy required	Moderate discomfort; non-narcotic analgesia required	Severe discomfort; or narcotic analgesia required with symptomatic improvement
Other Dermatologic	Grade I – Mild	Grade II – Moderate	Grade III - Severe

Event	Grade I – Mild	Grade II – Moderate	Grade III - Severe
Dermatitis	Rash is present but asymptomatic	Rash is symptomatic (pruritus/pain) but does not interfere with function	Rash is symptomatic and interferes with function
Other Urinalysis	Grade I – Mild	Grade II – Moderate	Grade III – Severe
Proteinuria: Random Urine	1+	2+ - 3+	4+
Proteinuria: 24 Hour Urine	200 mg – 1 g loss/day or <0.3% or <3 g/L	>1 – 2 g loss/day or 0.3% - 1.0% or 3 – 10 g/L	>2 g loss/day or >1.0% or >10 g/L
Hematuria (In The Absence Of Vaginal Bleeding)	Microscopic only, 6-10 rbc/hpf	>10 rbc/hpf	Gross, with or without clots; or RBC casts
Other Miscellaneous	Grade I – Mild	Grade II – Moderate	Grade III - Severe
Malaise	Malaise that is easily tolerated	Malaise that interferes with daily activity	Malaise that prevents daily activity

1. These values for neutropenia are values that have been used in other live attenuated dengue vaccine trials

14 Schedule of Procedures Post Each Vaccination

Day post vaccination	0	3	8	10	12	14	16	21	28	56	90	150[1]	180
Vaccinate	X												X[2]
CBC with differential	X	X	X	X	X	X	X						X[2]
ALT	X		X	X	X	X	X						X[2]
Creatinine	X				X								X[2]
PT/PTT	X		X		X		X						X[2]
Virology	X		X	X	X	X	X	(X)[3]					X[2]
Immunology	X	X	X		X			X	X	X	X	X	X
Pregnancy test	X							X	X	X	X	X	X
Physical examination	X	X	X	X	X	X	X	X	X		X	X	X
HIV test												X	
HCV test												X	
Hep B Surface Ag												X	

1. Occurs only post-vaccination #1
2. This procedure will occur on Day 180 post-vaccination #1 only
3. Virology will be done only if virus was recovered from the Day 16 sample
