# Supplementary material for: In a randomized trial, the live attenuated tetravalent dengue vaccine TV003 is well-tolerated and highly immunogenic in subjects with flavivirus exposure prior to vaccination
Source: PLoS Negl Trop Dis. 2017 May 8;11(5):e0005584. doi: 10.1371/journal.pntd.0005584 (PMC5436874; doi:10.1371/journal.pntd.0005584)

**Figure S1.** Mean peak neutralizing antibody titer to DENV-1 (A), DENV-2 (B), DENV-3 (C), and DENV-4 (C) after one dose of TV003 to flavivirus-exposed subjects. Groups: Single (n = 35), ≥ 2 (n = 6), YF-doc (n = 12), YF-sero (n = 17), DENV-doc (n = 8), DENV-sero (n = 3).

No differences were observed by one-way ANOVA.


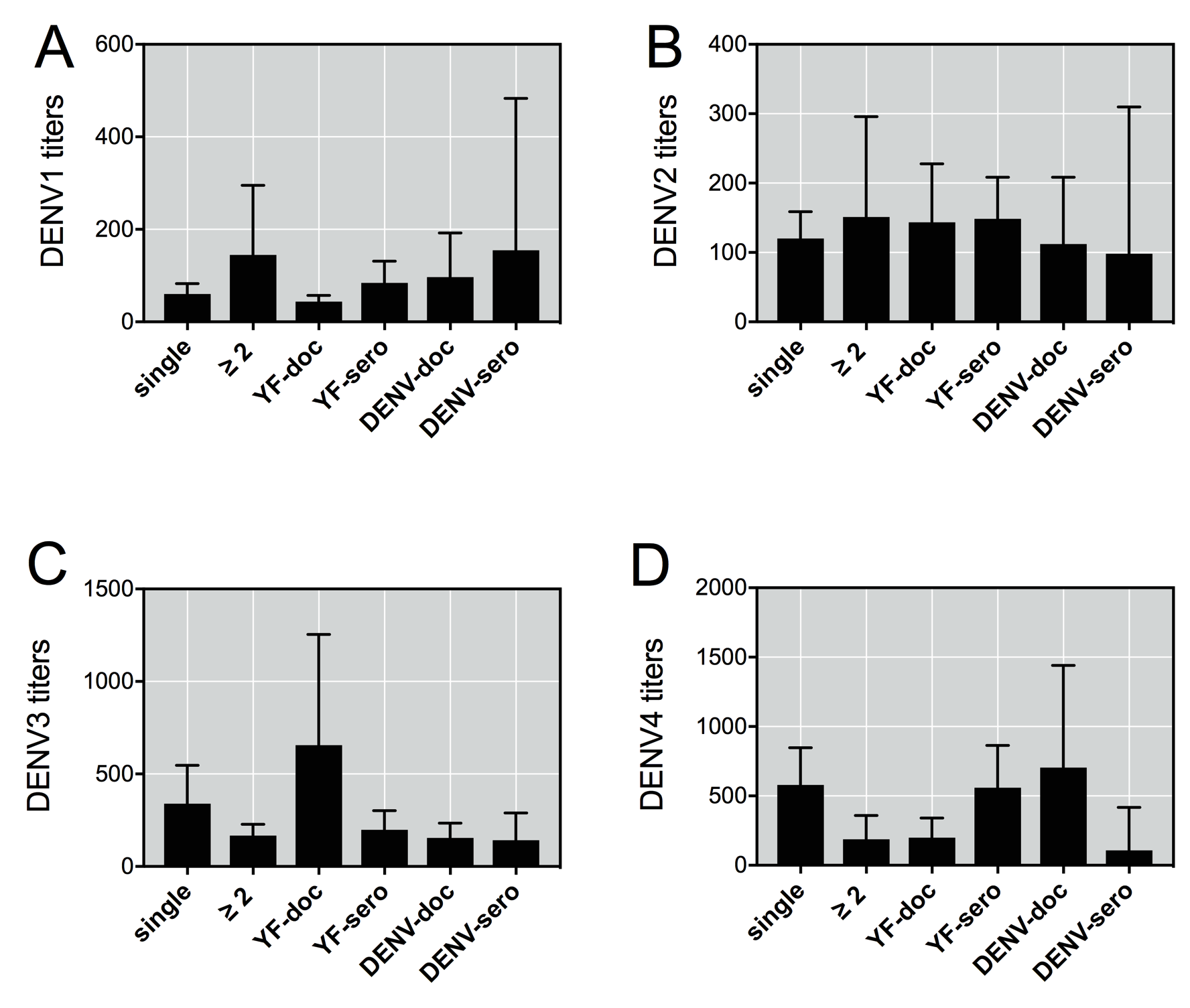

Supplement: S1 Fig — Mean peak neutralizing antibody titer to DENV-1 (A), -2 (B), -3 (C), and -4 (D) after one dose of TV003 to flavivirus-exposed subjects. (DOCX) [file pntd.0005584.s007.docx]
